# Excess glucocorticoids inhibit murine bone turnover via modulating the immunometabolism of the skeletal microenvironment

**DOI:** 10.1172/JCI166795

**Published:** 2024-03-21

**Authors:** Xu Li, Tongzhou Liang, Bingyang Dai, Liang Chang, Yuan Zhang, Shiwen Hu, Jiaxin Guo, Shunxiang Xu, Lizhen Zheng, Hao Yao, Hong Lian, Yu Nie, Ye Li, Xuan He, Zhi Yao, Wenxue Tong, Xinluan Wang, Dick Ho Kiu Chow, Jiankun Xu, Ling Qin

**Affiliations:** 1 Musculoskeletal Research Laboratory, Department of Orthopedics and Traumatology, Faculty of Medicine,; 2 Innovative Orthopedic Biomaterial and Drug Translational Research Laboratory, Li Ka Shing Institute of Health Sciences, and; 3 School of Biomedical Sciences, Faculty of Medicine, The Chinese University of Hong Kong, Hong Kong, China.; 4 Beijing Key Laboratory of Preclinical Research and Evaluation for Cardiovascular Implant Materials, Animal Experimental Centre, and; 5 State Key Laboratory of Cardiovascular Disease, Fuwai Hospital, National Center for Cardiovascular Diseases, Chinese Academy of Medical Sciences and Peking Union Medical College, Beijing, China.; 6 Centre for Translational Medicine Research and Development, Shenzhen Institute of Advanced Technology, Chinese Academy of Sciences, Shenzhen, China.

**Keywords:** Bone biology, Bone disease, Fatty acid oxidation, Osteoporosis

## Abstract

Elevated bone resorption and diminished bone formation have been recognized as the primary features of glucocorticoid-associated skeletal disorders. However, the direct effects of excess glucocorticoids on bone turnover remain unclear. Here, we explored the outcomes of exogenous glucocorticoid treatment on bone loss and delayed fracture healing in mice and found that reduced bone turnover was a dominant feature, resulting in a net loss of bone mass. The primary effect of glucocorticoids on osteogenic differentiation was not inhibitory; instead, they cooperated with macrophages to facilitate osteogenesis. Impaired local nutrient status — notably, obstructed fatty acid transportation — was a key factor contributing to glucocorticoid-induced impairment of bone turnover in vivo. Furthermore, fatty acid oxidation in macrophages fueled the ability of glucocorticoid-liganded receptors to enter the nucleus and then promoted the expression of *BMP2*, a key cytokine that facilitates osteogenesis. Metabolic reprogramming by localized fatty acid delivery partly rescued glucocorticoid-induced pathology by restoring a healthier immune-metabolic milieu. These data provide insights into the multifactorial metabolic mechanisms by which glucocorticoids generate skeletal disorders, thus suggesting possible therapeutic avenues.

## Abstract 2

Excess glucocorticoids impair local nutrient status and reduce bone turnover, leading to a net loss of bone mass and delayed fracture healing in mice.

## Graphical abstract

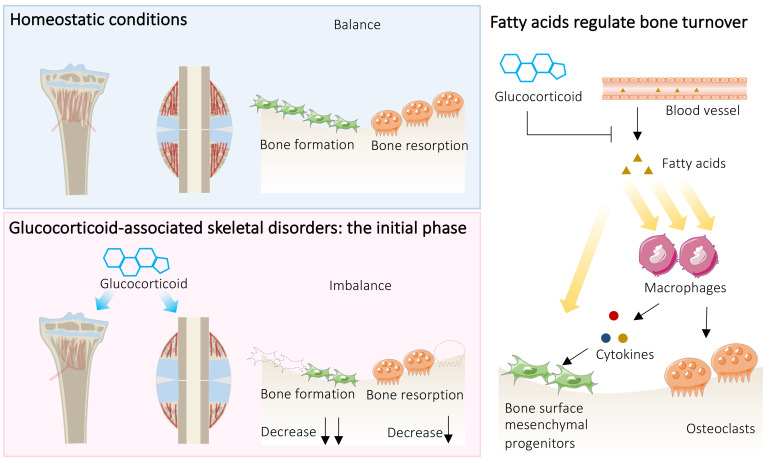

## Introduction

Synthetic glucocorticoids (GCs), acting as antiinflammatory and immunomodulatory agents, are the mainstays in the treatment of immune-related disorders (e.g., rheumatoid arthritis and Graves’ disease). GCs are also prescribed to patients after organ transplantation and to those hospitalized with severe coronavirus diseases like COVID-19 (1–5). However, high-dose and chronic administration of GCs can lead to bone loss in about 40% of patients, increasing the risk of fracture (6), making GC-induced osteoporosis the most common form of secondary osteoporosis (7).

A formidable number of studies have concluded that activated bone resorption and inhibition of bone formation are the primary features of GC-induced bone loss (7–10). Even so, uncertainties about their mechanism of action in this regard still exist. Clinical evidence shows that indices of bone formation are rapidly decreased upon treatment with GCs, while indices of bone resorption may also be downregulated (11, 12). Notably, a randomized and open-label clinical trial showed that the bone-forming agent teriparatide significantly increases spinal bone mineral density, microstructure, and finite element–derived strength compared with the antiresorptive agent risedronate in men with GC-induced bone loss (13). These results suggest that GC-induced bone loss may be more reliant on reduced bone anabolism rather than elevation of bone catabolism. Further, while it has been reported that GCs directly enhance osteoclastogenesis in vitro (14, 15), others have proposed that they impair bone resorption in vivo (16–18). Consequently, the effects of GCs on bone turnover remain controversial. GCs act primarily as ligands for the GC receptor (GR), a member of the nuclear receptor superfamily of transcription factors, to execute various functions, including influencing cell metabolism (19, 20). As nearly all nucleated cells express GR (1), one possible explanation is that the in vivo outcomes of GC action reflect the combined effects of reciprocal interconnectivity of receptor signaling in different cell types. In vivo, microenvironmental constraints may also indirectly mediate cellular metabolism, which may also influence the impact of GC signaling on skeletal homeostasis. Less well studied is the underlying regulation of the GC-associated nutritional milieu during bone turnover, although local blood flow deficiency in osteonecrosis has been postulated as a pathogenic mechanism (21–23). A recent study has reported that the nutrient availability from the vasculature determines skeletal progenitor cell fate (24). In addition, the effect of changes in skeletal immune function on defects in bone turnover is also understudied.

Here, we investigated bone turnover during GC treatment using a well-established bone loss model as well as a fracture model. We found that GCs unexpectedly inhibited bone turnover, which revises the previous understanding that elevations in bone turnover driven by increased bone resorption are the primary means by which GCs induce bone loss. We also found that this inhibition of bone loss is due to deficiencies in fatty acid transport in the bone microenvironment, leading to an impaired immune-metabolic milieu and dysregulated bone formation. Targeting this deficiency by supplying fatty acids directly to the callus via nanoparticle delivery improved fracture healing. Based on these results, we propose that GC-induced bone loss is attributable to reduced bone turnover and diminished bone formation. In other words, the defect is attributable to an indirect effect on the rate of bone anabolism as a deficient energy supply affects the local immune system, rather than due to direct effects on bone catabolism.

## Results

### GCs inhibit trabecular bone turnover in male mice.

**Figure 1:**
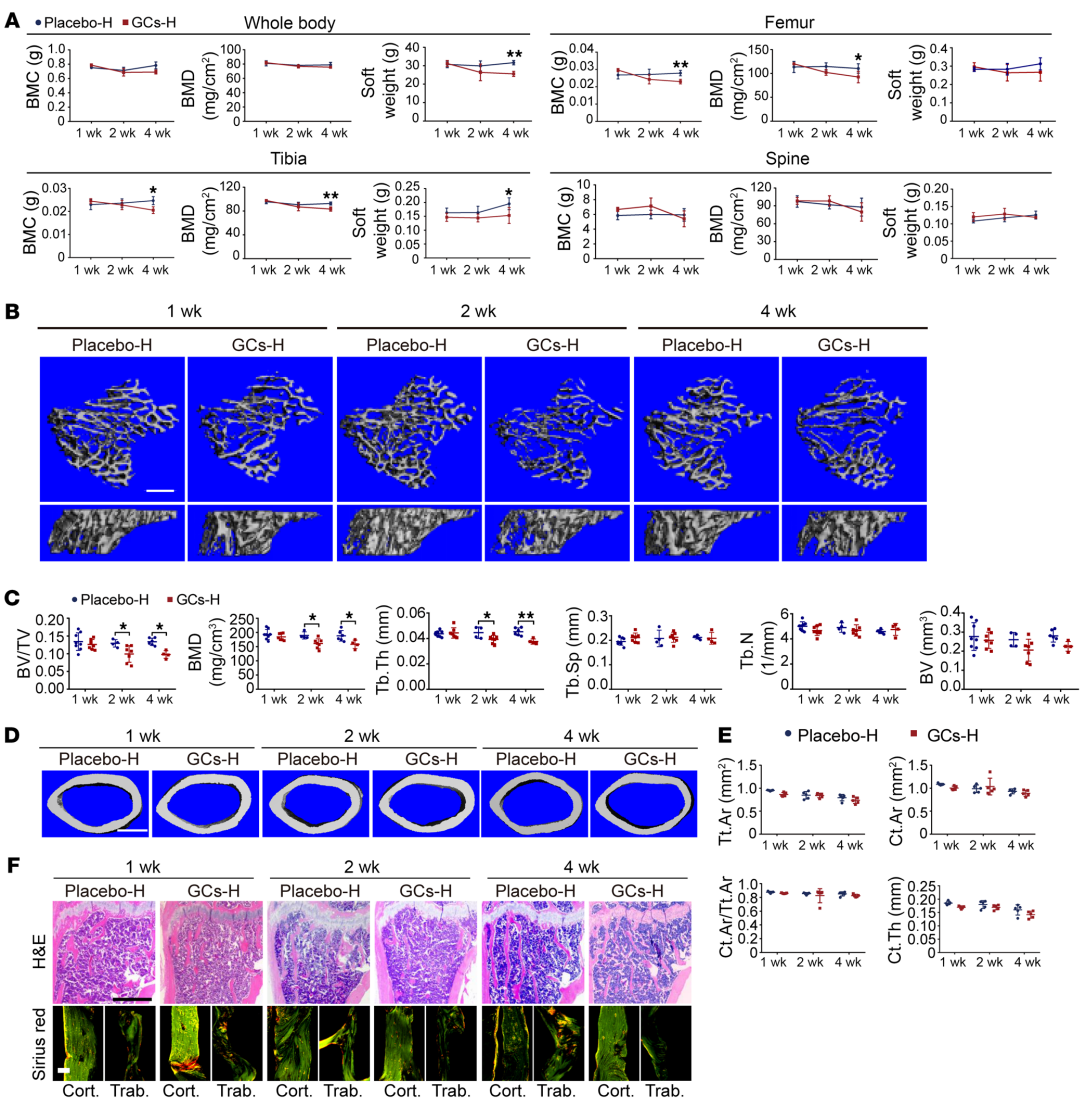
Excess GC induces rapid trabecular bone loss in male mice. (**A**–**F**) Bone phenotypes of mice treated with placebo (Placebo-H) or high-dose (6.25 mg/kg/d) prednisolone (GCs-H). (**A**) Bone mass and soft tissue weight of the body, tibia, spine, and femur as measured by dual-energy x-ray absorptiometry (*n* = 3–7 per time point). BMC, bone mineral content; BMD, bone mineral density. (**B** and **C**) Representative micro-CT images (**B**) and quantification (**C**) of bone volume fraction (BV/TV), bone mineral density (BMD), trabecular thickness (Tb.Th), trabecular separation (Tb.Sp), trabecular number (Tb.N), and bone volume (BV) in trabecula (*n* = 4–8 per time point; scale bar: 500 μm). (**D** and **E**) Representative micro-CT images (**D**) and quantification (**E**) of the periosteal envelope (Tt.Ar), cortical bone area (Ct.Ar), cortical area fraction (Ct.Ar/Tt.Ar), and average cortical thickness (Ct.Th) in cortical bone (*n* = 5 per time point; scale bar: 500 μm). (**F**) Representative H&E (scale bar: 500 μm) and sirius red (scale bar: 50 μm) staining of tibia. Data are mean ± SD. *P < 0.05, **P < 0.01 by 2-way ANOVA (**A**, **C**, and **E**) with Bonferroni’s post hoc test.

The subcutaneous implantation of a slow-release prednisolone pellet is the general approach for creating a model of GC-induced bone loss, with doses typically ranging from 1.4 mg/kg/d to 11 mg/kg/d (25, 26). Based on our preliminary study, we selected doses of 2.1 mg/kg/d (low dose) and 6.25 mg/kg/d (high dose) in the subsequent experiments (Supplemental Figure 1A; supplemental material available online with this article; https://doi.org/10.1172/JCI166795DS1). We observed a dose- and time-dependent bone loss effect in male mice. Continuous delivery of prednisolone at the low dose did not show any significant changes in cortical and trabecular bone quality parameters, as assessed by micro-CT (Supplemental Figure 1, B and C) and histomorphometric analysis (Supplemental Figure 1D). In contrast, by dual-energy x-ray absorptiometry analysis, we found that changes in bone quality were more sensitive when subjected to the high-dose prednisolone treatment (Figure 1A). Further, by micro-CT analysis and histological staining, we observed a decrease in trabecular bone mass from week 1 to week 2 after treatment (Figure 1, B, C, and F). Trabecular bone was more sensitive to the high dose of prednisolone, while the cortical bone mass did not show measurable changes (Figure 1, D–F).

**Figure 2:**
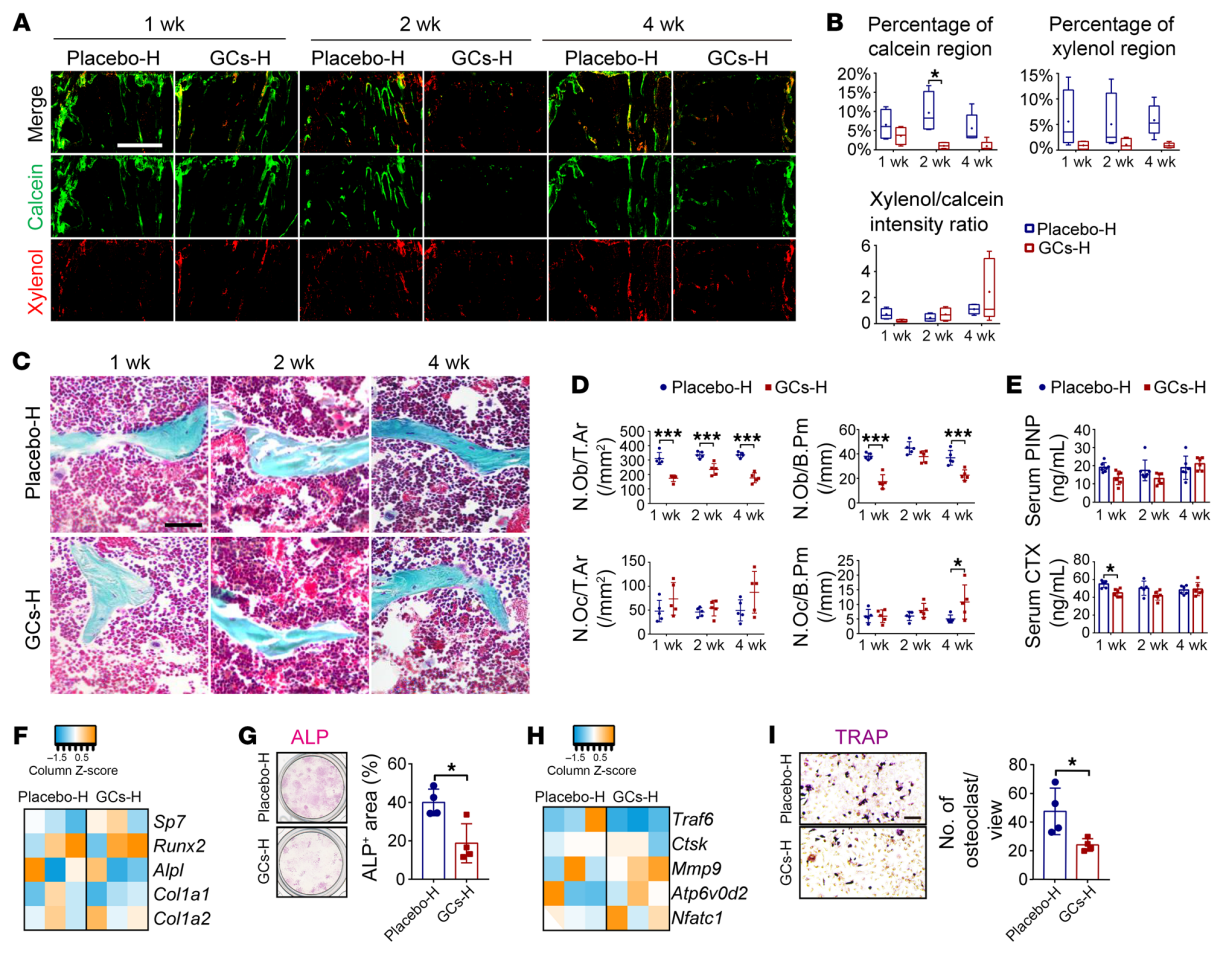
GC-induced bone loss coincides with reduced bone turnover. (**A** and **B**) Representative images of calcein/xylenol double labeling (**A**) and assessment of bone remodeling (**B**) (*n* = 4–5; scale bar: 500 μm). (**C** and **D**) Representative Goldner trichrome staining (**C**) (scale bar: 50 μm) and quantification of the number of osteoblasts per trabecular area (N.Ob/T.Ar), number of osteoblasts per bone perimeter (N.Ob/B.Pm), number of osteoclasts per trabecular area (N.Oc/T.Ar), and number of osteoclasts per trabecular area (N.Oc/B.Pm) (**D**) (*n* = 5). (**E**) Serum concentration of PINP and CTX1 in the circulation (*n* = 5–7 per time point). (**F** and **G**) Ex vivo osteogenic induction, transcription levels (**F**, at day 3, *n* = 3), and alkaline phosphatase (ALP) staining (**G**, at day 5, *n* = 4) of bone surface adherent cells. (**H** and **I**) Ex vivo osteoclastogenic induction, transcription levels (**H**, at day 3, *n* = 3), and TRAP staining (**I**, at day 5, *n* = 4) of bone surface cells (scale bar: 50 μm). Data are mean ± SD. *P < 0.05, ***P < 0.001 by 2-way ANOVA (**B**, **D**, and **E**) with Bonferroni’s post hoc test or 2-tailed Student’s *t* test (**G** and **I**).

Calcein/xylenol double labeling revealed inhibition of new bone formation from week 1 to week 4 after high-dose prednisolone treatment (Figure 2, A and B). During the period of rapid bone loss (weeks 1–2), the number of bone surface osteoblasts decreased, and the number of osteoclasts was not altered (Figure 1, C and D), while immunohistochemical staining revealed a slightly decreased cathepsin K–positive (Ctsk-positive) area in trabecular bone (Supplemental Figure 2) and serum carboxy-terminal collagen cross-links (CTX) revealed a decline in bone resorption activities after high-dose prednisolone treatment (Figure 2E). Furthermore, although we observed no significant change at the transcriptional levels of osteogenesis- and osteoclastogenesis-related markers (Figure 2, F and H), bone surface cells isolated from mice treated with high-dose prednisolone for 2 weeks exhibited a diminished capacity for osteogenesis and osteoclastogenesis during ex vivo culture, as evidenced by alkaline phosphatase (ALP) and tartrate-resistant acid phosphatase (TRAP) staining (Figure 2, G and I).

These findings collectively demonstrate that GC administration hinders both bone formation and resorption, resulting in a net effect of bone mass reduction during the initial phase of exposure.

### GCs inhibit bone turnover of male mice during callus formation.

**Figure 3:**
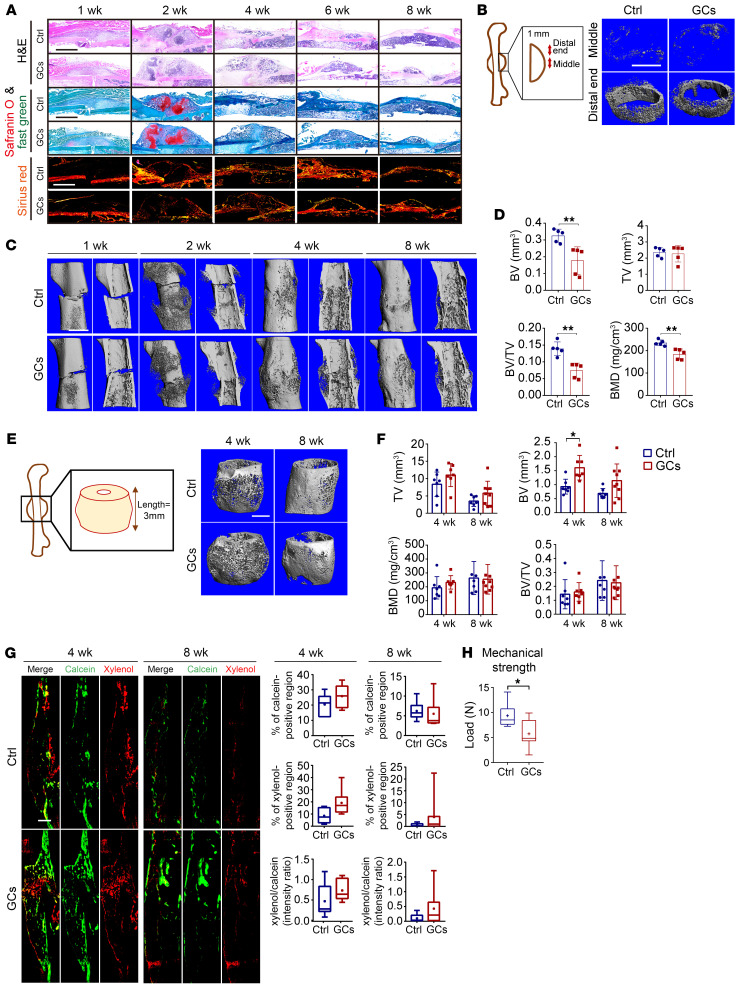
GCs inhibit callus formation and delay fracture healing. (**A**) Representative H&E, Safranin O and fast green, and sirius red staining of callus (scale bars: 1 mm). (**B**) Representative micro-CT images in callus (scale bar: 500 μm). (**C** and **D**) Representative micro-CT images (**C**) and quantification (**D**) of BV, total volume (TV), BV/TV, and BMD in distal end calluses (*n* = 5; scale bar: 500 μm) at week 2 post-fracture. (**E** and **F**) Representative micro-CT images (**E**) and quantification (**F**) of BV, TV, BV/TV, and BMD (*n* = 7–9 per time point; scale bar: 500 μm) in calluses of weeks 4 and 8 post-fracture. (**G**) Representative images of calcein/xylenol double labeling and assessment of bone remodeling (*n* = 6–8 per time point; scale bar: 200 μm). (**H**) Mechanical strength of femora at week 4 (*n* = 7). Data are mean ± SD. *P < 0.05, **P < 0.01 by 2-tailed Student’s *t* test (**D**, **G**, and **H**) or 2-way ANOVA (**F**) with Bonferroni’s post hoc test.

Next, we established a GC-associated fracture healing model in male mice (Supplemental Figure 3A). Boney callus formed gradually from week 2 to week 4 post-fracture, as revealed by x-ray images, and its size decreased during remodeling from week 4 to week 8 (Supplemental Figure 3, B and C). Daily injection of prednisolone at the same high dose as above (6.25 mg/kg/d) did not significantly alter the callus size. In the initial phase of callus formation (week 2 post-fracture), we found a weak alignment of collagen fibers as determined by sirius red staining in the prednisolone-treated group (Figure 3A). Furthermore, via micro-CT analysis, we found a decreased formation of bony tissue, as revealed by decreased bone quality parameters in the distal-end callus (Figure 3, B–D). During the callus remodeling phase from week 4 to week 8 post-fracture, the prednisolone group displayed a larger bone volume (BV) of bony callus compared with the control group (Figure 3, E and F). Calcein/xylenol double labeling suggested that, in conjunction with ongoing remodeling, there was a gradual decline in calcium deposition from week 4 to week 8 post-fracture (Figure 3G). A biomechanical test confirmed a lower maximum compressive load at week 4 post-fracture in the prednisolone-treated group than in the control group (Figure 3H). Together, these data show that prednisolone exposure delays the process of callus mineralization, leading to poorer healing and weaker bones (at least during a later phase of fracture healing) (Supplemental Figure 3D).

**Figure 4:**
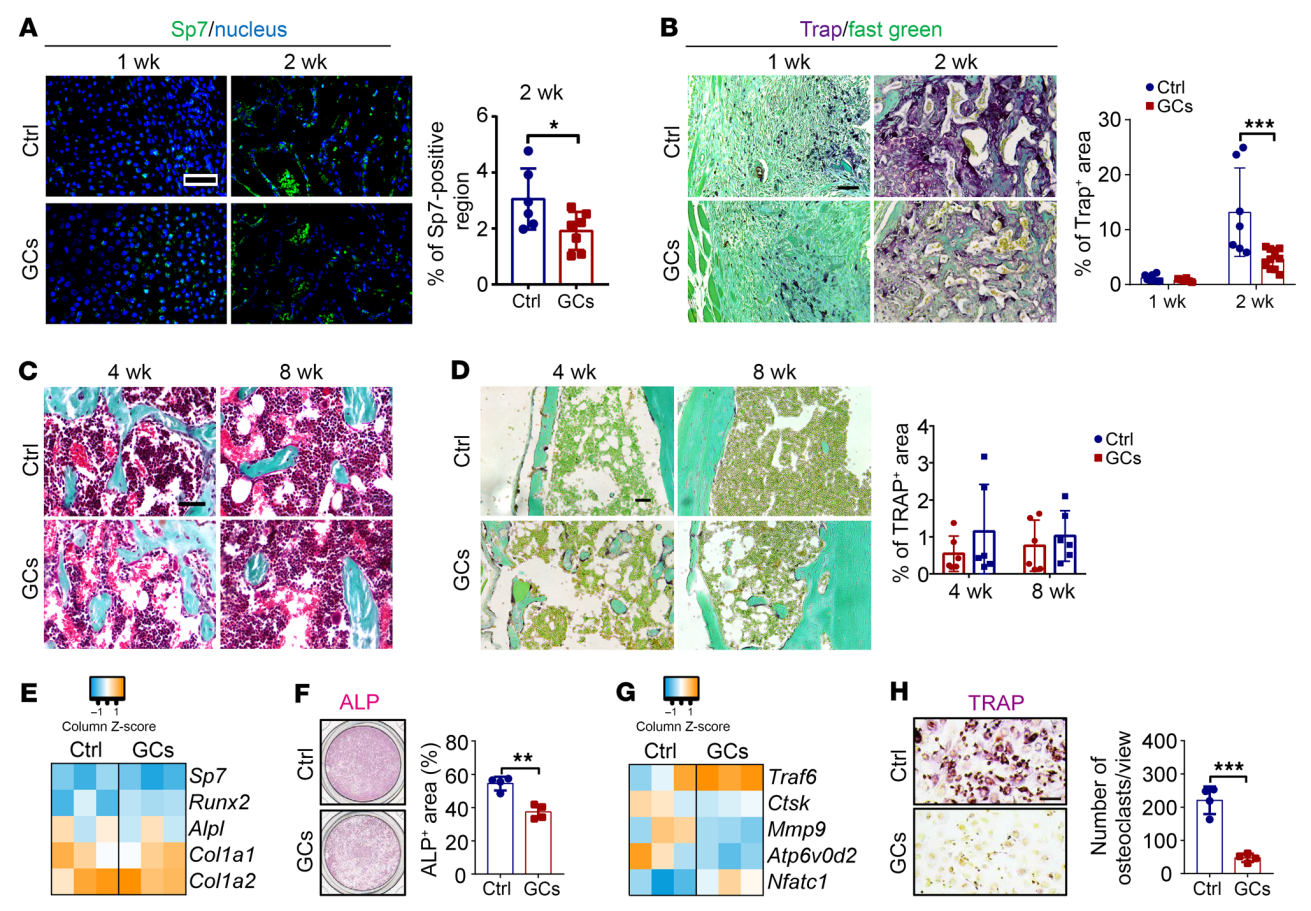
GCs impede bone formation and resorption during the rapid phase of callus formation. (**A**) Representative immunofluorescence images of Sp7 (scale bar: 50 μm) and quantification of positive region (*n* = 6–7). (**B**) Representative TRAP staining images (scale bar: 100 μm) and quantification of positive region (*n* = 6–12 per time point). (**C**) Representative Goldner trichrome from weeks 4 and 8 post-fracture calluses (scale bar: 50 μm). (**D**) TRAP staining from weeks 4 and 8 post-fracture calluses (scale bar: 50 μm) and quantification of positive region (*n* = 6 per time point). (**E** and **F**) Ex vivo osteogenic induction, transcription levels (**E**, at day 3, *n* = 3), and representative ALP staining images (**F**, at day 5, *n* = 4) of week 2 callus cells. (**G** and **H**) Ex vivo osteoclastogenic induction, transcription levels (**G**, at day 3, *n* = 3), and representative TRAP staining images (**H**, at day 5, *n* = 4) of week 2 callus cells (scale bar: 50 μm). Ctrl, placebo group; GCs, prednisolone-treated group. Data are mean ± SD. *P < 0.05, **P < 0.01, ***P < 0.001 by 2-tailed Student’s *t* test (**A**, **F**, and **H**) or 2-way ANOVA (**B** and **D**) with Bonferroni’s post hoc test.

We also found decreased Sp7^+^ bone-forming area and TRAP^+^ bone-resorbing area in the prednisolone-treated mice at week 2 post-fracture compared with the control group (Figure 4, A and B). Upon callus remodeling over time, the differences in bone formation and resorption between the control group and prednisolone group gradually became less apparent (Figure 4, C and D). Strikingly, callus cells of the prednisolone-treated mice at 2 weeks post-fracture displayed a significantly lower capability of undergoing osteogenesis and osteoclastogenesis during ex vivo culture compared with callus cells of the control group (Figure 4, E–H).

These data suggest that GC administration impedes bone formation and resorption during the rapid phase of callus formation, thus suppressing overall bone turnover and resulting in a net effect of delayed fracture healing.

### GCs also suppress bone turnover in female mice.

We validated the impact of GCs on bone turnover in female mice to determine whether it aligns with the effects observed in male mice. Before affecting cortical bone (evidenced by micro-CT; Supplemental Figure 4A), prednisolone administration in female mice primarily resulted in loss of trabecular bone volume (Supplemental Figure 4, B–D) from week 2 to week 4 after treatment. Histologically, prednisolone led to a decrease in the number of osteoblasts on the bone surface at week 2 (Supplemental Figure 4C), with no significant increase in the number of osteoclasts (Supplemental Figure 4E). We also examined the direct effects of prednisolone on bone formation and resorption during the early stage of callus formation (week 2). Consistent with the results in male mice, although the overall size of the callus in female mice did not show a significant change (Supplemental Figure 5A), there was a significant reduction in BV (Supplemental Figure 5B) and bone area (Supplemental Figure 5C) within the hard callus at week 2 post-fracture. Notably, we observed significantly smaller areas of Sp7^+^ bone-forming cells and TRAP^+^ bone-resorbing cells in the prednisolone-treated group compared with the control group (Supplemental Figure 5, D and E). These findings suggest that GCs affect bone turnover in a sex-independent manner.

### Direct effects of GCs on osteogenesis.

We next tested whether the reduced bone turnover upon GC treatment was attributable to a direct inhibition of the intrinsic skeletogenic potential. Resident bone-forming progenitors (i.e., bone surface mesenchymal progenitors [BSMPs]) play a vital role in physiological bone formation, and central bone marrow mesenchymal progenitors (CMPs) (27) were cultured under three GC dosing conditions: GC deprivation (0 M dexamethasone [Dex]), standard dosing (10^–8^ M Dex, which is regarded as a normal dose for inducing osteogenic differentiation) (28, 29), and high dosing (10^–6^ M Dex). Dex exposure with a standard or high dose did not alter the transcription levels of osteogenic markers in CMPs and only slightly increased them in BSMPs on day 3 after osteogenic differentiation (Supplemental Figure 6, A–D). As shown by ALP and alizarin red S (ARS) staining, the addition of Dex did not alter the early osteogenic differentiation on day 5, but it promoted calcium nodule formation on day 10 (Supplemental Figure 6, E and F). These data suggest that the direct inhibitory effect of GC exposure on osteogenesis is not prominent in comparison with the control group. We then speculated that the discrepancy between the in vivo and in vitro results might result from effects on the local environment in vivo that is not replicated in vitro.

### GCs modulate bone formation by regulating macrophage function.

**Figure 5:**
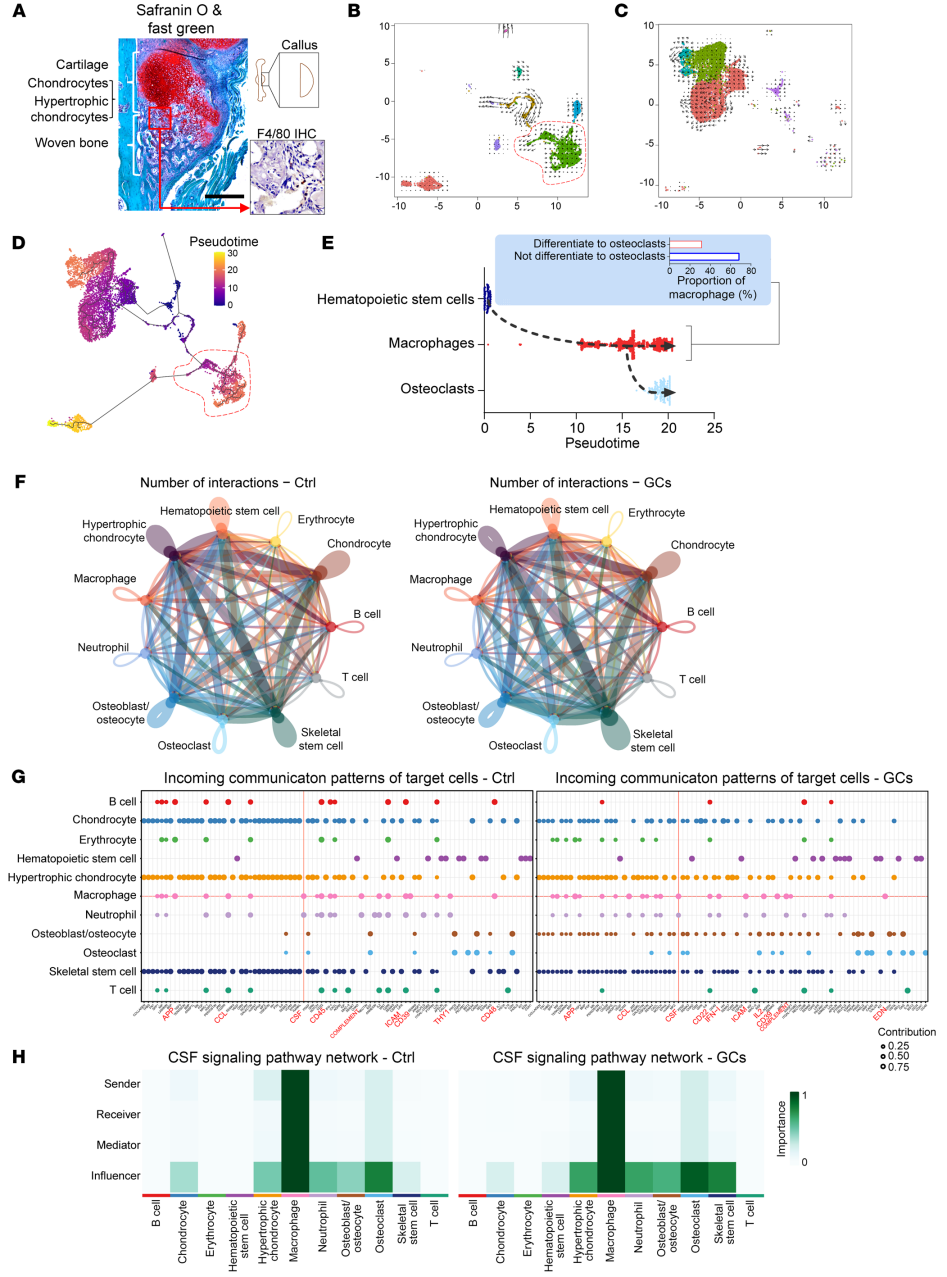
Intercellular communications of the macrophage-associated immune milieu within the callus. (**A**) Representative histological characterization of F4/80-positive macrophage pool within the callus (scale bar: 500 μm). (**B**–**H**) Single-cell RNA sequencing analysis of cell populations and communications. (**B** and **C**) RNA velocity field onto the *t*-distributed stochastic neighbor embedding plot of hematopoietic cells (**B**) and osteolineage (**C**). (**D**) Pseudotime ordering of hematopoietic and bone formatting cell lineages. (**E**) Pseudotime ordering of hematopoietic stem cells, macrophages, and osteoclasts. (**F**) Interaction patterns of callus cells (classified by numbers). (**G**) Incoming communication patterns of target cells. (**H**) CSF signaling pathway network of callus cells. Ctrl, placebo group; GCs, prednisolone-treated group.

We next used single-cell RNA sequencing (scRNA-Seq; 10× Genomics) to investigate the principal cell types involved that modulate bone turnover during GC exposure. In this manner we identified a small percentage of osteolineage cells (skeletal stem cells, less than 3%) and a large proportion of hematopoietic cells (over 97%, including ~75% neutrophil and ~9% macrophage), suggesting that a small population of skeletal stem cells are sufficient to support the slow bone turnover (Supplemental Figure 7, A–G). De novo formation of skeletal tissue during fracture healing was broadly activated by bone-forming lineages. We identified 16 clusters, including 6 osteolineage cell clusters (~65%) and 10 hematopoietic-lineage cell clusters (~35%) (Supplemental Figure 7, H–K). Notably, while the percentage of neutrophils (~4%) dropped dramatically in the callus, macrophages (~18%) became a major proportion of the hematopoietic-lineage cell clusters (Supplemental Figure 7, J–M). Using immunohistochemistry, we also found that bone macrophages were distributed in the space among hypertrophic cartilage and newly formed bone (Figure 5A). Pseudotime trajectory and RNA velocity analysis suggested that most macrophages (~70%) were not directly involved in osteoclast differentiation (Figure 5, B–E). Cell communication analysis suggested that macrophages exhibited a greater number of interactions with osteolineages, particularly with skeletal stem cells, as compared with osteoclasts (Figure 5F). Notably, Gene Ontology (GO) term pathway analyses of macrophages between control and the GC-treated groups showed enrichment in pathways in connective tissue development, cartilage development, skeletal system morphogenesis, and bone development (Supplemental Figure 7, N and O). These data suggested an active involvement of the macrophage pool during the rapid callus formation. Among the main signaling pathways involved in macrophage communication, the contributions of signals (e.g., App, Ccl, and Csf) were enriched (Figure 5G). Further analysis revealed that Csf signaling exhibited remarkable specificity in regulating macrophage communication (Figure 5H).

**Figure 6:**
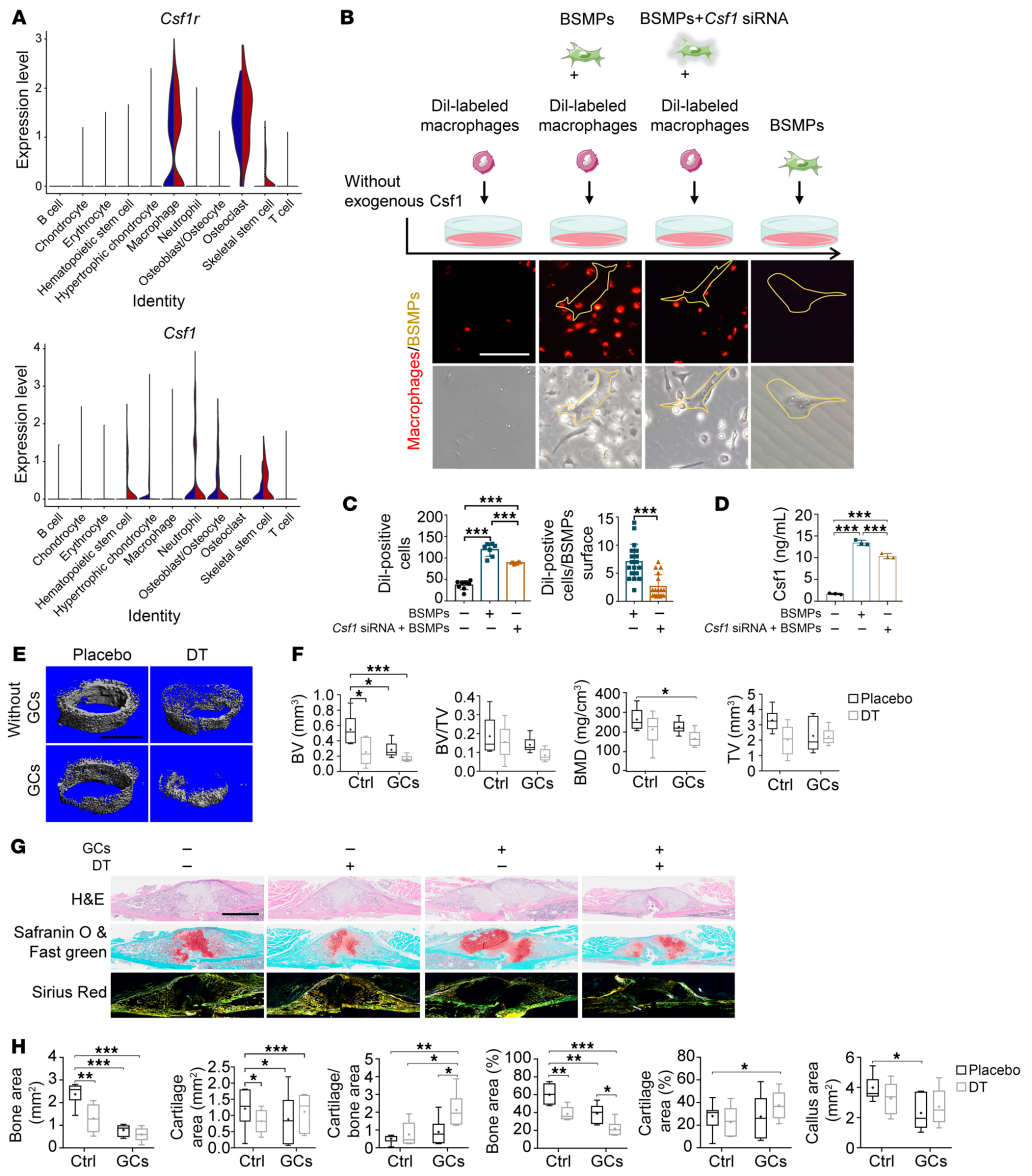
GCs alter the macrophage-associated immune milieu to affect bone formation. (**A**) Violin plots of normalized expression of *Csf1r* and *Csf1* in the callus. (**B** and **C**) Representative micrographs (**B**) and quantifications (**C**) of DiI-labeled macrophages (red) with the coculture of BSMPs or *Csf1* siRNA–transfected BSMPs (scale bar: 50 μm). (**D**) Supernatant concentration of Csf1 from BSMPs or *Csf1* siRNA–transfected BSMPs (*n* = 3). (**E** and **F**) Representative micro-CT images (**E**) and quantification (**F**) of BMD, BV/TV, and BV in the callus with or without macrophage depletion (*n* = 6; scale bar: 500 μm). DT, diphtheria toxin. (**G** and **H**) Representative H&E, Safranin O and fast green, and sirius red staining (**G**) and quantification (**H**) of week 2 calluses with or without macrophage depletion in CD11b-DTR mice (*n* = 5–7; scale bar: 1 mm). Data are mean ± SD. *P < 0.05, **P < 0.01, ***P < 0.001 by 2-tailed Student’s *t* test (**C**) or 1-way ANOVA (**C** and **D**) or 2-way ANOVA (**F** and **H**) with Bonferroni’s post hoc test.

Given that macrophage proliferation, migration, and differentiation are mainly controlled by macrophage colony-stimulating factor (Csf1) (30), we performed transcriptional analysis of the gene encoding Csf1 receptor (Csf1r) and found that it was predominantly expressed by macrophages (Figure 6A and Supplemental Figure 8A). Conversely, osteolineages, particularly skeletal stem cells, were identified as the primary source of Csf1.

In the absence of Csf1, macrophages displayed poor migration capacity and showed low adherence during in vitro culturing (Figure 6B and Supplemental Figure 8B). Conversely, the presence of BSMPs induced the migration of macrophages and increased the number of adherent macrophages (Figure 6, B and C). Enzyme-linked immunosorbent assay (ELISA) validated the production and secretion of Csf1 by BSMPs (Figure 6D). Suppressing Csf1 production from BSMPs using *Csf1* siRNA counteracted the recruitment of macrophages, as observed in direct coculture and Transwell migration assays (Figure 6, B–D, and Supplemental Figure 8B). Furthermore, GC-pretreated BSMPs exhibited elevated Csf1 production and enhanced macrophage recruitment (Supplemental Figure 8, C–E). These findings further validate the scRNA-Seq data, pinpointing the ability of BSMPs to support the local accumulation of macrophages through Csf1 secretion.

To investigate the role of macrophages in bone formation in vivo, we used CD11b-DTR mice to systemically deplete macrophages during the early phase of callus formation (week 1 to week 2 post-fracture). Additionally, in wild-type mice, we administered clodronate liposomes locally to deplete macrophages within the same phase of callus formation (validated by immunofluorescent staining of F4/80) (Supplemental Figure 9A). The depletion of macrophages either systemically or locally resulted in a further reduction in bone area (as observed histologically) and bone quality parameters (as validated by micro-CT) in both the control and GC-treated groups (Figure 6, E–H, and Supplemental Figure 9, B–E). In contrast, the delivery of Csf1 increased the number of macrophages, as shown by immunofluorescent staining of F4/80-positive area (Supplemental Figure 9F), leading to an increase in total bone area without changing the bone quality parameters (bone mineral density and bone volume fraction [BV/TV]), especially in the control groups (Supplemental Figure 9, G–J). These data show the role of the macrophage pool in promoting bone formation under conditions with or without excess GC exposure.

Next, we validated the involvement of macrophages in osteogenesis in vitro. In the presence of macrophages, treatment with Dex significantly upregulated the transcription levels of osteogenic genes in BSMPs (e.g., *Sp7*) and CMPs on day 3 and increased the ALP-positive area on day 5 (Supplemental Figure 10, A and B). Consistent with previous findings that GCs induce polarization of macrophages into an M2-like phenotype (1, 31), we also identified increased expression of *Cd163*, *Cd204*, *Arg1*, *IL10*, *Tgfb*, *IL4*, and *BMP2*, and decreased expression of *IL6*, *IL1*, and *Tnfa* (Supplemental Figure 10C). Conditioned medium from macrophages pretreated with high-dose Dex significantly upregulated the transcription of osteogenic genes (Supplemental Figure 10D). Among the upregulated cytokines in the polarized macrophages (Supplemental Figure 10E), BMP2 is known to directly promote osteogenesis (32). Conditioned medium derived from macrophages with BMP2 knockdown exhibited a counteractive effect on the osteogenic properties of the macrophage secretome induced by Dex (Supplemental Figure 10F).

### GC-associated nutritional niche affects bone turnover.

A decreased blood volume was detected in both the bone loss and fracture models (Supplemental Figure 11, A–C). In contrast to GC-induced promotion of osteogenesis, GC exposure for 24 hours disturbed the angiogenesis of endothelial cells in vitro (33–35), as suggested by diminished tube formation, an altered transcriptional pattern, suppression of nitric oxide production through a suppressive GC response element, and weakened expression of ZO1 and VE-cadherin, but increased permeability (Supplemental Figure 11, D–N). Notably, neither the cell cycle nor apoptosis of the endothelial cells was affected (Supplemental Figure 11, F and G).

Given that the obstruction of vascular invasion suppresses bone formation by affecting nutrient transport (i.e., glucose, amino acids, and lipids) (24), these results inspired us to investigate the possibility that disruption of nutrient transport might synchronously lower bone formation and resorption levels. To test this hypothesis, we measured local nutrient transport in vivo in our GC-induced bone loss and associated fracture healing models. It is known that reciprocal metabolism of fatty acids, glucose, and amino acids supports skeletal energy homeostasis (24, 36–38). Local marrow glucose was not decreased in the bone loss and delayed fracture healing models (Supplemental Figure 12, A and B). In contrast, although GC exposure did not lower circulating fatty acids, the local marrow fatty acid levels decreased significantly at week 2 (Supplemental Figure 12, C and D), implying a deficiency in their localized transport in the bone niche.

**Figure 7:**
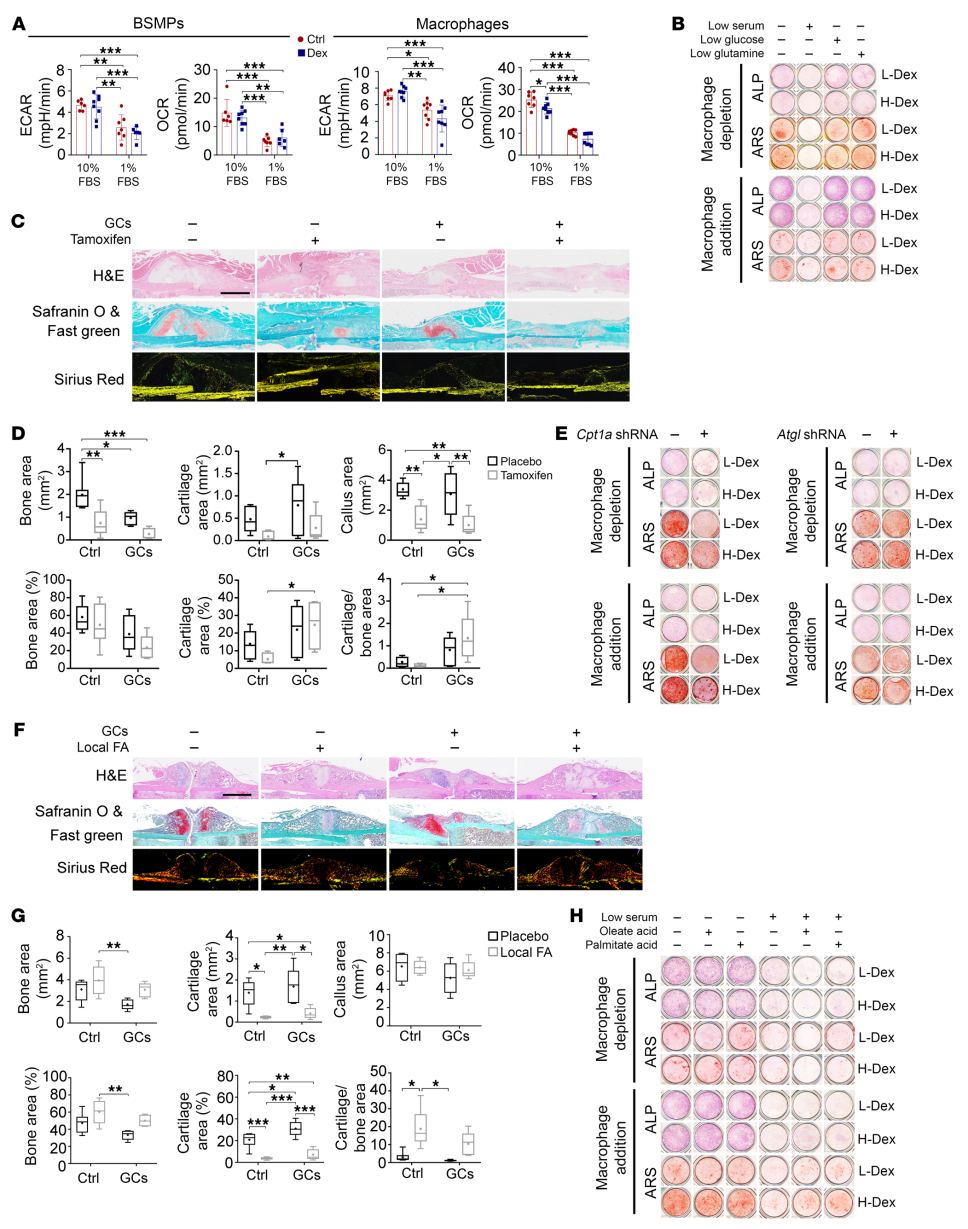
Reduced fatty acid metabolism inhibits bone formation. (**A**) Extracellular acidification rate (ECAR) and oxygen consumption rate (OCR) tracings from BSMPs and macrophages under normal-serum (10% FBS) or low-serum (1%) culture condition (*n* = 6–8). (**B**) Osteogenic differentiation of BSMPs (with or without macrophage addition) exposed to control or different nutritional stresses, assessed by ALP or ARS staining. (**C** and **D**) Representative H&E, Safranin O and fast green, and sirius red staining (**C**) and quantification (**D**) of week 2 calluses with or without depletion of Cpt1a (through tamoxifen) in *Ubc-Cre-ERT2 Cpt1a^fl/fl^* mice (*n* = 5–7; scale bar: 1 mm). (**E**) Representative ALP or ARS staining images of BSMPs (with or without macrophage addition) transfected with vehicle, *Cpt1a* shRNA, or *Atgl* shRNA during osteogenic differentiation. (**F** and **G**) Representative H&E, Safranin O and fast green, and sirius red staining (**F**) and quantification (**G**) of week 2 calluses with or without local injection of fatty acids (FA) in wild-type mice (*n* = 5–6; scale bar: 1 mm). (**H**) Representative ALP or ARS staining images of BSMPs (with or without addition of macrophages) exposed to normal-serum or low-serum condition, with or without the addition of fatty acids (oleate acid and palmitate acid) during osteogenic differentiation. Data are mean ± SD. *P < 0.05, **P < 0.01, ***P < 0.001 by 2-way ANOVA (**A**, **D**, and **G**) with Bonferroni’s post hoc test.

We then explored whether fatty acid deficiency attenuated bone turnover. Cpt1a (a key transferase of fatty acids) and Glut1 (a transporter of glucose) were more highly expressed in bone-forming areas than in cartilage areas, suggesting the requirement of fatty acids and glucose oxidation during bone formation (Supplemental Figure 12E). Serum (i.e., FBS) is the source of fatty acids in culture that provides 2%–3% of the fatty acid (39). We also measured the concentrations of fatty acids in FBS (Supplemental Table 1). Serum deprivation (1% FBS) remarkably decreased the energy metabolism, as revealed by reductions in the oxygen consumption rate and the extracellular acidification rate of both BSMPs and bone marrow–derived macrophages (Figure 7A). Compared with glucose and glutamine deprivation, serum deprivation had a more pronounced effect on cell viability and osteogenesis of BSMPs, regardless of whether they were cocultured with bone marrow–derived macrophages (Figure 7B and Supplemental Figure 13, A and B). Additionally, serum deprivation inhibited osteoclastogenesis (Supplemental Figure 13C).

To achieve time-specific deletion of *Cpt1a* expression during callus formation, we generated *Ubc-Cre-ERT2* *Cpt1a^fl/fl^* mice and administrated tamoxifen at specific time points (week 1 to week 2 post-fracture), mimicking the status of fatty acid metabolism deficiency. The inhibition of fatty acid metabolism in vivo resulted in a significant reduction in callus size and bony area (Figure 7, C and D), as well as a decrease in BV in newly formed calluses (Supplemental Figure 13D). Such effects were observed in both control and GC groups. Similarly, transfection with shRNA to knock down *Cpt1a* expression in culture also inhibited both osteogenesis of BSMPs and osteoclastogenesis (Figure 7E and Supplemental Figure 13E). The inhibition of endogenous fatty acid synthesis by transfection of *Atgl* (adipose triglyceride lipase) shRNA did not obviously alter the osteogenesis capacity of BSMPs but slightly attenuated osteoclastogenesis (Figure 7E and Supplemental Figure 13F). Apart from suppressing the osteogenic differentiation of BSMPs, knockdown of *Cpt1a* expression resulted in reduced viability of BSMPs, regardless of whether they were cocultured with bone marrow–derived macrophages (Supplemental Figure 13, G and H). Conversely, knockdown of *Atgl* expression had negligible effects on the viability of BSMPs (Supplemental Figure 13, G and H). Therefore, bone formation and resorption primarily depend on the uptake of fatty acids from external sources. Deficiency in fatty acid oxidation impairs both osteogenesis and osteoclastogenesis.

We also tested whether the joint effects of GCs and serum deprivation were able to explain the phenotypes of osteogenesis in vivo. Except for the role in the supplementation of energy substrates, from a transcriptional perspective serum deprivation slightly upregulated the expression of some osteogenic genes during osteogenic induction in vitro (Supplemental Figure 14A). As the actions of GCs on the expression of osteogenic genes (with or without macrophage coculturing) were also selectively upregulated (Supplemental Figure 6D and Supplemental Figure 10B), the combined effects on the expression of osteogenic genes in vivo should theoretically be selectively upregulated. Indeed, the expression profile confirmed the in vivo upregulation of osteogenic genes in bone-forming cells (Supplemental Figure 14B).

To evaluate whether exogenous fatty acids could rescue the GC-reduced callus formation, we locally injected fatty acids into the fracture sites. Fatty acid delivery increased bone area and the ratio of woven bone to cartilage area in vivo (Figure 7, F and G, and Supplemental Figure 13A). We conducted in vitro cell interventions using palmitic acid (the predominant saturated fatty acid in the bloodstream) and oleic acid (the predominant unsaturated fatty acid in the bloodstream), respectively. Extra fatty acids did not rescue the impaired osteogenesis and cell survival of pure BSMPs (Figure 7H and Supplemental Figure 15B). Notably, supplying fatty acids (particularly palmitic acid) to the BSMPs containing macrophages partially restored the impaired osteogenesis, especially in the presence of GCs, without affecting cell viability (Figure 7H and Supplemental Figure 15C). As fatty acids also function as signaling molecules that activate free fatty acid receptors (FFARs) (40), to test the involvement of FFAR signaling, we activated FFAR1 by using GW9508. However, this approach did not rescue the reduced osteogenesis and cell viability under conditions of serum deprivation, implying the predominant role of fatty acids as an energy source during bone turnover (Supplemental Figure 15, D–F). These results suggest that availability of fatty acids affects bone turnover. Although a decline in fatty acid metabolism impairs osteogenesis, the addition of exogenous fatty acid cannot directly and rapidly rescue the inhibition of osteogenesis of pure BSMPs.

### Nutrient uptake patterns of BSMPs and the local macrophage pool.

**Figure 8:**
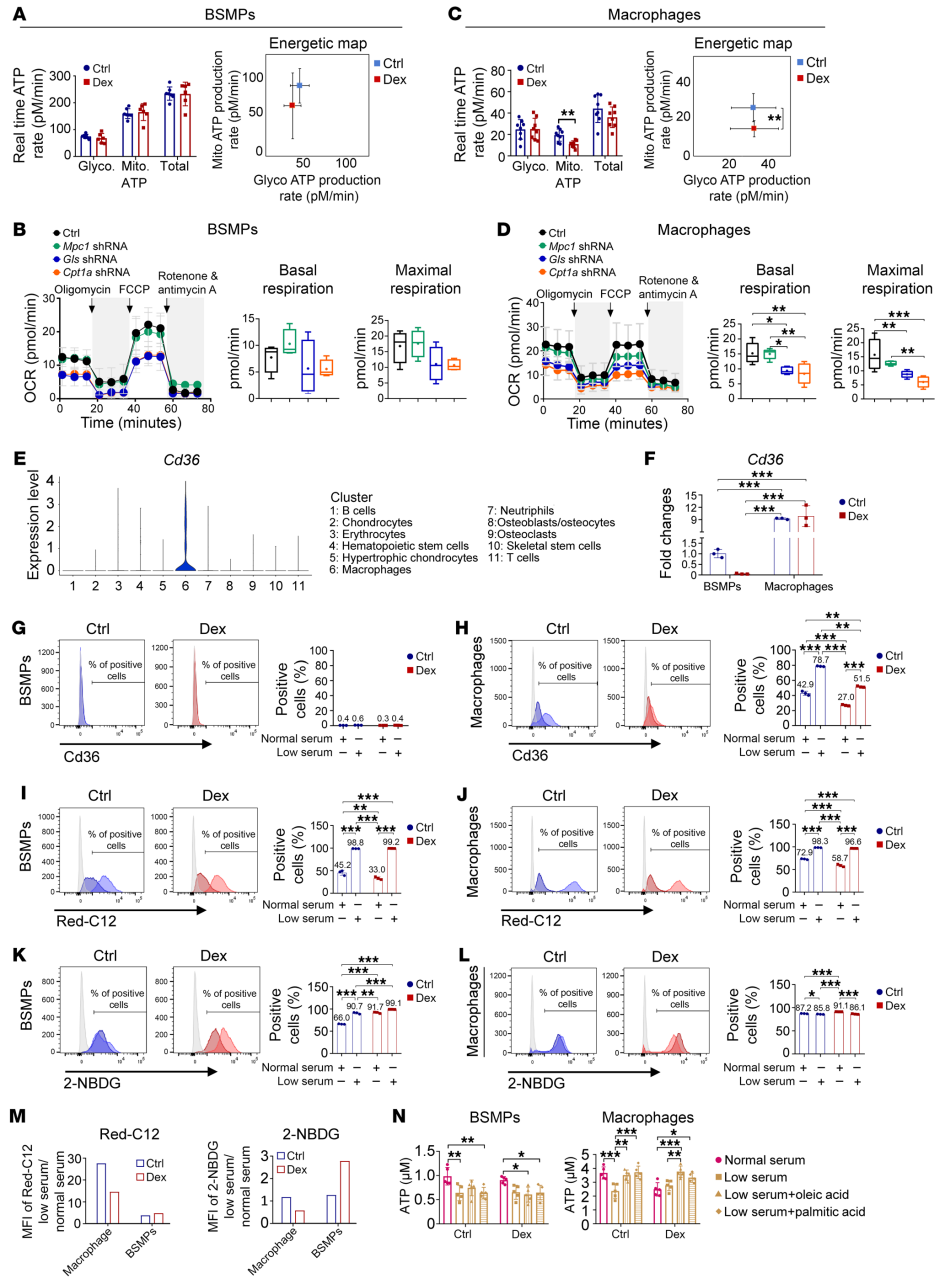
Macrophages uptake fatty acid more rapidly than bone-forming cells. (**A** and **C**) Real-time ATP production assays of BSMPs (**A**) and macrophages (**C**) with or without Dex exposure (*n* = 6–8). (**B** and **D**) Cellular substrate oxidation measurement in BSMPs (**B**) and macrophages (**D**) transfected with vehicle (Ctrl), *Mpc1* shRNA, *Gls* shRNA, or *Cpt1a* shRNA (*n* = 4–6). (**E**) Violin plots of normalized expression of *Cd36*. (**F**) Gene expression of *Cd36* on BSMPs and macrophages in culture (*n* = 3). (**G** and **H**) Flow cytometry assessment of Cd36 expression on BSMPs (**G**) and macrophages (**H**) treated under the culture of different serum conditions (*n* = 3). (**I** and **J**) Flow cytometry assessment of fatty acid (Red C12) uptake by BSMPs (**I**) (*n* = 3) and macrophages (**J**) (*n* = 3). (**K** and **L**) Flow cytometry assessment of glucose (2-NBDG) uptake by BSMPs (**K**) and macrophages (**L**) (*n* = 3). (**M**) Uptake patterns of glucose and fatty acids by BSMPs and macrophages in normal- and low-serum conditions, quantified by flow cytometry. (**N**) Intracellular ATP in BSMPs and macrophages, exposed to normal-serum or low-serum condition, with or without the addition of fatty acids (oleate acid and palmitate acid) (*n* = 5). Dex, 10^–6^ M dexamethasone. Data are mean ± SD. *P < 0.05, **P < 0.01, ***P < 0.001 by 1-way ANOVA (**B** and **D**) or 2-way ANOVA (**A**, **C**, **E**, **F**, **H**–**L**, and **N**) with Bonferroni’s post hoc test.

Next, we analyzed the energy metabolism profile of BSMPs and bone marrow–derived macrophages. We detected elevated transcription levels of fatty acid oxidation–associated genes in macrophages and bone-forming cells (skeletal stem cells/osteoblasts/osteocytes) in vivo (Supplemental Figure 16, A and B). The energy metabolism of BSMPs relied more on mitochondrial ATP than glycolytic ATP (Figure 8A). GC treatment did not significantly alter the basal real-time production rate of glycolytic and mitochondrial ATP (Figure 8A) and the metabolism gene signatures of BSMPs (Supplemental Figure 16C). To investigate the reliance on various forms of mitochondrial oxidation, we used *Mpc1*, *Gls*, and *Cpt1a* shRNA transfection to knock down the corresponding transporters responsible for glucose, glutamine, and fatty acid oxidation, respectively, in BSMPs. The results demonstrated that knockdown of *Cpt1a* expression inhibited both basal respiration and maximal respiration in BSMPs, confirming the contribution of fatty acid oxidation to mitochondrial ATP production (Figure 8B and Supplemental Figure 16D). Although enhanced mitochondrial oxidation is involved in IL-4–activated M2a macrophages (41), GC-activated M2c macrophages showed decreased mitochondrial oxidation without changes in glycolysis (Figure 8C). Compared with BSMPs, macrophages presented a higher degree of fatty acid oxidation (Figure 8D and Supplemental Figure 16F), and GC exposure modestly upregulated the transcription of genes related to fatty acid metabolism (Supplemental Figure 16E).

As fatty acids play a dominant role in fueling BSMPs and their related macrophage pools, we hypothesized that the efficiency of exogenous fatty acids to rescue serum deprivation–inhibited osteogenesis might be associated with the uptake rate of fatty acids. Endocytosis mediated by Cd36 (a fatty acid translocase) is a vital process supporting the intracellular transport of fatty acids (42). The transcription levels of Cd36 in macrophages were significantly higher than those in bone-forming cells (Figure 8, E and F). By flow cytometry analysis we found that a substantial frequency of macrophages expressed the surface transporter Cd36, while no surface expression was detected in BSMPs (Figure 8, G and H).

We next analyzed the nutrient uptake patterns of BSMPs and their associated macrophage pool. Compared with BSMPs, macrophages took up more fluorescent fatty acids and glucose over 24 hours (Figure 8, I–L). GC reduced fatty acid uptake while increasing glucose uptake in both BSMPs and macrophages (Figure 8, I–L). Serum deprivation stimulated fatty acid uptake in both macrophages and BSMPs, with macrophages showing a more notable increase (Figure 8, I–M). Concomitantly, delivery of an adequate dose of fatty acids ultimately heightened intracellular ATP in macrophages but not in BSMPs under serum deprivation conditions (Figure 8N). Differential uptake patterns of fatty acids suggest that macrophages have higher reactivity to exogenous fatty acids than BSMPs. The proper distribution and uptake of fatty acid by BSMPs and macrophages may depend on the optimal energy transport microenvironment in the body, which is challenging to achieve by directly delivering fatty acids.

### Exogenous fatty acids shape M2c macrophage polarization to affect osteogenesis.

We then sought to determine whether and how fatty acid oxidation influences the phenotypes of GC-treated bone marrow–derived macrophages. Suppression of fatty acid endocytosis using the Cd36 inhibitor sulfo-*N*-succinimidyl oleate (SSO) or fatty acid transport from the cytosol into mitochondria using *Cpt1a* shRNA diminished GC-induced M2c phenotypes and reduced the production of BMP2 (Supplemental Figure 17, A–D). The β-oxidation of fatty acid metabolism relies on the transfer of electron-reducing equivalents to the mitochondrial electron transport chain (ETC) to generate ATP (43). The addition of antimycin A (inhibitor of ETC complex III) and oligomycin (inhibitor of ETC complex V) abolished the upregulation of M2c-associated genes and the production of BMP2 (Supplemental Figure 17, E and F), suggesting the requirement of an intact ETC. Notably, deficiency of fatty acid oxidation was not a generalized defect for cytokine production, as macrophages under transfection with *Cpt1a* shRNA were competent to produce IL-6 (Supplemental Figure 17, G and H). In contrast to the effect on BMP2 production, activated oxidative phosphorylation using FCCP diminished IL-6 production, while blockade of complex V using oligomycin promoted it (Supplemental Figure 17, I and J), indicating exquisite specificity of fatty acid oxidation for promotion of the M2c phenotype and BMP2 production.

**Figure 9:**
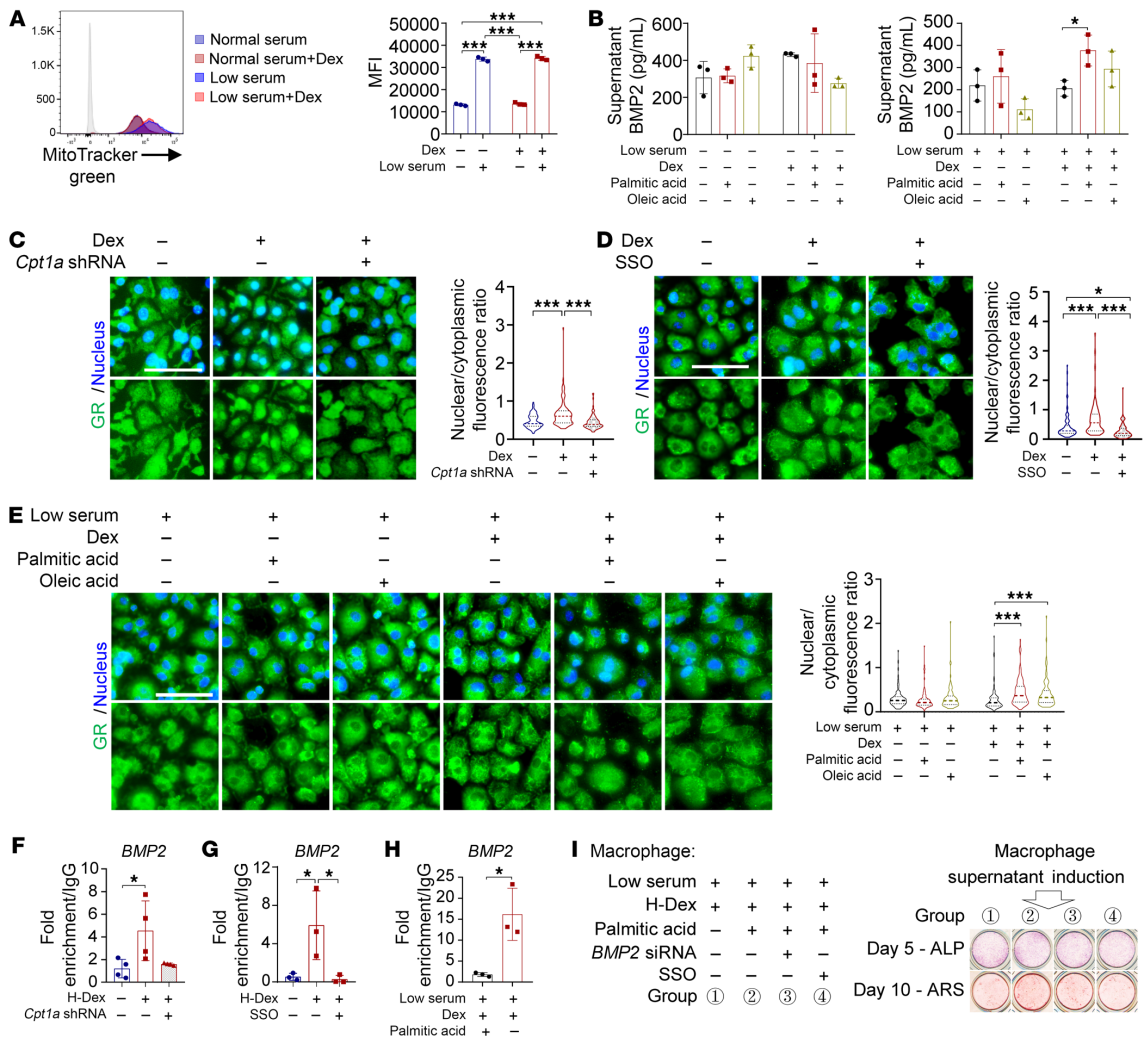
Fatty acids fuel macrophages to promote osteogenesis. (**A**) Mitochondrial content in macrophages under different culture conditions, as measured by MitoTracker Green (Thermo Fisher Scientific) (*n* = 3). (**B**) BMP2 production from macrophages exposed to palmitic acid and/or oleic acid (*n* = 3). (**C**–**E**) Representative images of macrophages stained for GR and quantification of nuclear localization (*n* = 100; scale bars: 50 μm). (**C**) Cells were transfected with vehicle or *Cpt1a* shRNA (*n* = 100). (**D**) Cells were treated with SSO (*n* = 100). (**E**) Cells were treated with palmitic acid and/or oleic acid (*n* = 100). (**F**–**H**) Occupancy of GR at the *BMP2* promoter of macrophages exposed to different culture conditions. (**F**) Cells were transfected with *Cpt1a* shRNA (*n* = 4). (**G**) Cells were treated with SSO (*n* = 3). (**H**) Cells were treated with palmitic acid and/or oleic acid (*n* = 3). (**I**) ALP (at day 5) and ARS (at day 10) staining of BSMPs cultured with supernatants from low serum– and H-Dex–pretreated macrophages with the addition of palmitic acid, SSO, and/or *BMP2* siRNA. Low serum, 1% FBS culture condition; Dex, 10^–6^ M dexamethasone. Data are mean ± SD. *P < 0.05, **P < 0.01, ***P < 0.001 by 2-way ANOVA (**A**, **B**, and **E**), 1-way ANOVA (**C**, **D**, **F**, and **G**) with Bonferroni’s post hoc test, or 2-tailed Student’s *t* test (**H**).

GC exposure did not change the mitochondrial content of bone marrow–derived macrophages (Figure 9A). However, serum deprivation resulted in a compensatory increase in mitochondrial content and upregulated surface expression of Cd36 (Figure 8H and Figure 9A), providing the structural basis for the reactivity of exogenous fatty acids. Supplementation with appropriate concentrations of exogenous fatty acids (e.g., palmitic acid) and GCs in a low-serum culture medium increased the expression of M2c markers and BMP2 production in comparison with normal serum (Figure 9B and Supplemental Figure 17, K–N). The elevated expression of these markers was abolished by the Cd36 inhibitor SSO (Supplemental Figure 17, K and L).

Besides participating in mitochondrial oxidative phosphorylation, fatty acids are also involved in antiinflammatory processes regulated by membrane FFARs (40). But the antiinflammatory effect of activation of membrane receptor signal by GW9508 was weak (Supplemental Figure 17, O–R), supporting the dominant role of mitochondrial pathways fueling M2c phenotypes. The expression of antiinflammatory factors was not upregulated in macrophages in vivo, consistent with the phenotypes of macrophages in low–fatty acid oxidation conditions (Supplemental Figure 17, S–U).

We next investigated the mechanism by which GC signaling is regulated by fatty acid oxidation. Upon binding of GCs to the GR in the cytoplasm, GR translocates to the nucleus, where it regulates gene expression by binding to GC response elements within the promoters of various genes (44). Taking BMP2 as an example, both *Cpt1a* shRNA and SSO exposure, inhibiting fatty acid transport and oxidation, respectively, decreased the nuclear translocation of GR (Figure 9, C and D) and its binding to the *BMP2* promoter in macrophages (Figure 9, F and G). Under serum deprivation and Dex exposure, exogenous fatty acids increased the nuclear translocation of GR (Figure 9E) and its binding to the *BMP2* promoter (Figure 9H). Further, the treatment of conditional medium from *BMP2* siRNA– or SSO-pretreated macrophages abolished the upregulation of osteogenesis in BSMPs (Figure 9I). These findings suggest that GCs and fatty acid metabolism cooperatively program M2c polarization of macrophages, leading to BMP2 secretion, which in turn modulates osteogenesis.

### Targeted delivery of fatty acid–containing nanoparticles improves fracture healing.

**Figure 10:**
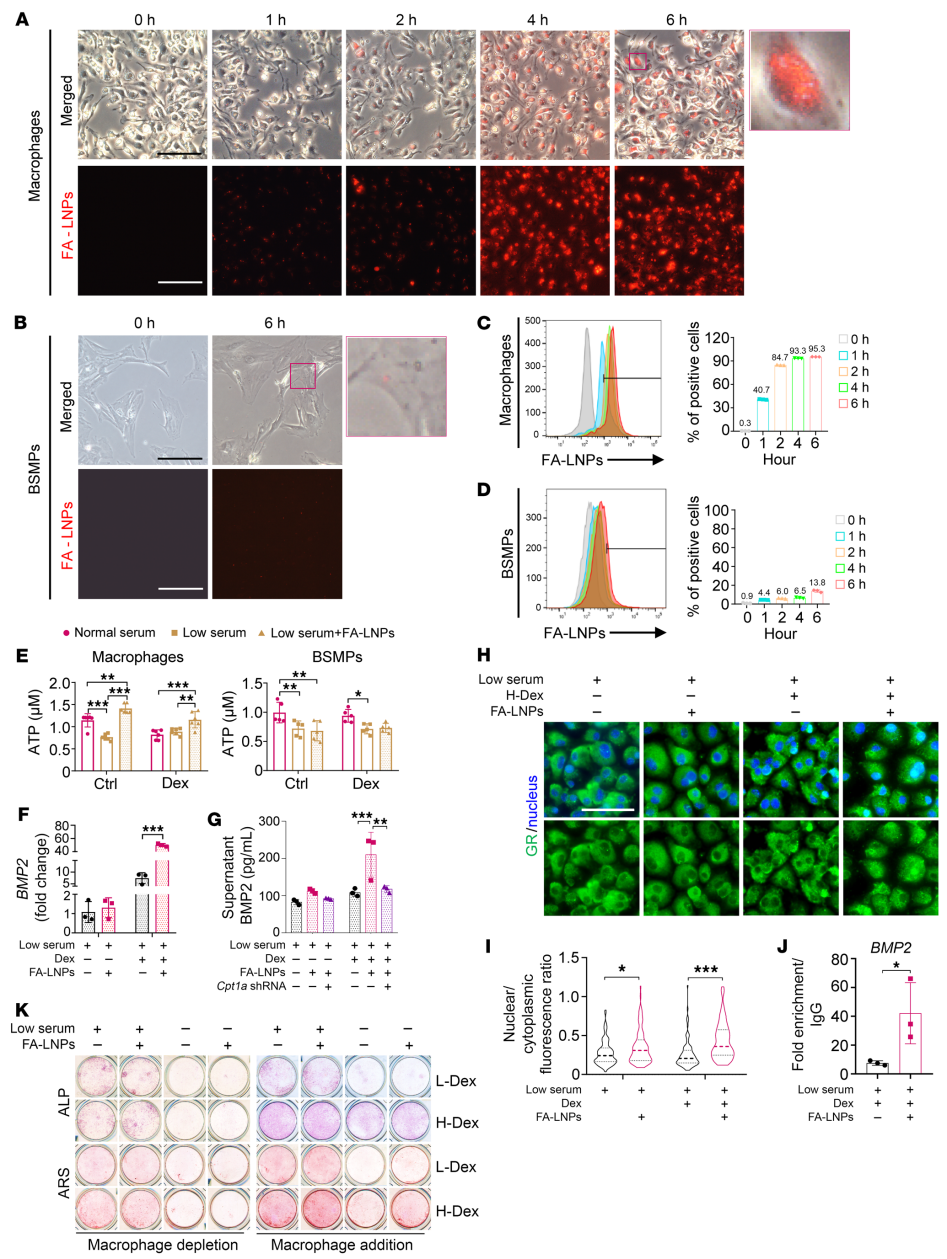
The fabricated fatty acid–containing nanoparticles reprogram macrophage metabolism. (**A** and **B**) Representative microscopy of FA-LNP (red) uptake by macrophages (**A**) or BSMPs (**B**) at specific time points (scale bars: 100 μm). (**C** and **D**) Flow cytometry quantification of FA-LNP uptake by macrophages (**C**) or BSMPs (**D**) (*n* = 3). (**E**) Intracellular ATP in macrophages and BSMPs, exposed to normal-serum or low-serum condition, with or without the addition of FA-LNPs (*n* = 5–6). (**F**) Under low-serum condition (1% FBS), transcription levels of *BMP2* in macrophages exposed to Dex and/or FA-LNPs (*n* = 3). (**G**) Under low-serum condition (1% FBS), BMP2 production from macrophages exposed to Dex and/or FA-LNPs (*n* = 3) with transfection of vehicle or *Cpt1a* shRNA. (**H** and **I**) Representative microscopy of macrophages (after exposure to different serum conditions, Dex, and/or FA-LNPs) stained for GR (**H**) and quantification of nuclear localization (**I**) (*n* = 100). Scale bar: 50 μm. (**J**) Occupancy of GR at the *BMP2* promoter of macrophages (after exposure to low-serum conditions, H-Dex, and/or FA-LNPs) (*n* = 3). (**K**) Osteogenic differentiation of BSMPs (with or without addition of macrophages) exposed to normal-serum or low-serum condition, with or without the addition of FA-LNPs, assessed by ALP or ARS staining. Dex, 10^–6^ M dexamethasone. *P < 0.05, **P < 0.01, ***P < 0.001 by 2-way ANOVA (**E**–**G** and **I**) with Bonferroni’s post hoc test or 2-tailed Student’s *t* test (**J**).

We sought to establish a systemic therapeutic strategy to tackle substantial clinical problems. Unexpectedly, a high-fat diet (45%) during callus formation (week 1 to week 2 post-fracture) did not rescue the bone quality deterioration upon GC exposure (Supplemental Figure 18, A and B). Next, to ensure local repletion of fatty acids and to avoid the risk of metabolic abnormalities caused by systematic lipid delivery, we specifically tailored macrophage-targeted fatty acid–containing lipid nanoparticles (FA-LNPs; 100 nm, 0 mV; Supplemental Figure 18C) for macrophage reprogramming and immune-osteogenic modulation. In the current study, FA-LNPs were effectively endocytosed by macrophages (95.3%) within 6 hours, while just 13.8% of BSMPs endocytosed FA-LNPs (Figure 10, A–D). FA-LNP delivery under serum deprivation significantly increased intracellular ATP in macrophages but not BSMP at 24 hours (Figure 10, E and F). FA-LNPs also increased nuclear translocation of GR and the binding to the promoter of *BMP2*, leading to an increased expression of M2c phenotypic genes and *BMP2* with the combined effects of GC exposure (Figure 10, F–J, and Supplemental Figure 18D). *Cpt1a* shRNA transfection counteracted FA-LNP–induced BMP2 production (Figure 8H). Although the cell viability was not altered (Supplemental Figure 18, E and F), elevated osteogenesis of BSMPs cocultured with macrophages was detected upon FA-LNP exposure (Figure 10K).

**Figure 11:**
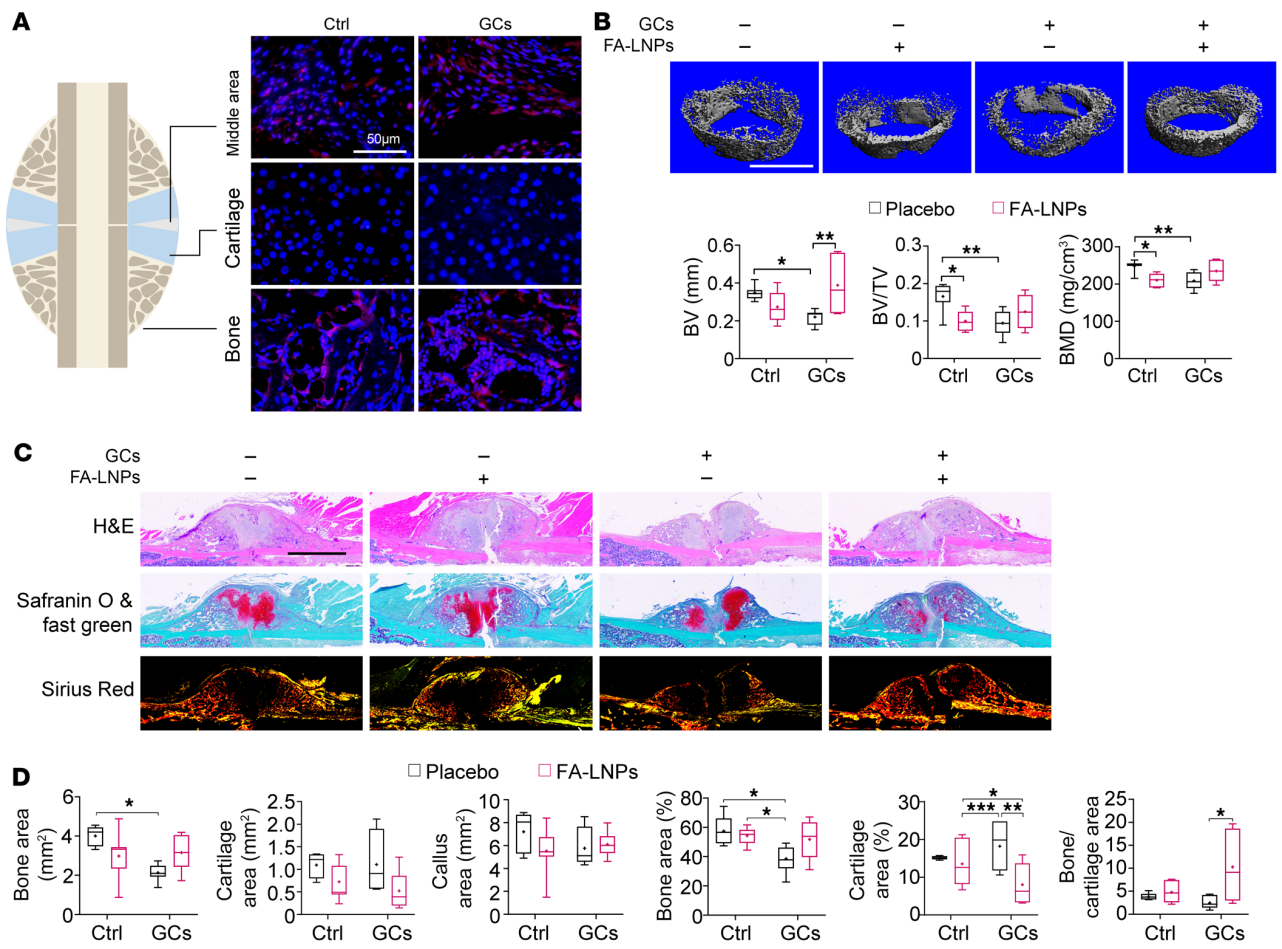
Macrophage-targeted metabolic reprogramming improves healing in the GC-associated fracture model. (**A**) Distribution of FA-LNPs (red) in week 2 callus revealed by red fluorescence (*n* = 5–8; scale bar: 50 μm). (**B**) Representative micro-CT images and quantification (distal ends) of BMD, BV/TV, and BV in the callus from mice treated with vehicle control (Ctrl) or 6.25 mg/kg/d of prednisolone (GCs) with or without FA-LNPs (*n* = 5–8; scale bar: 500 μm). (**C**) Representative H&E, Safranin O and fast green, and sirius red staining and quantification of callus at week 2 from mice with or without FA-LNPs (*n* = 5–8; scale bar: 1 mm). Data are mean ± SD. *P < 0.05, **P < 0.01, ***P < 0.001 by 2-way ANOVA (**B** and **C**) with Bonferroni’s post hoc test.

We next questioned whether FA-LNP injection could enhance hard callus formation in vivo. Once delivered to the fracture site, FA-LNPs were mainly endocytosed by cells from inflammatory and osteogenic areas, not cartilage areas (Figure 11A). The delivery of FA-LNPs significantly increased the BV of GC-treated group, as well as the bone/cartilage area ratio (Figure 11, B and C).

## Discussion

**Figure 12:**
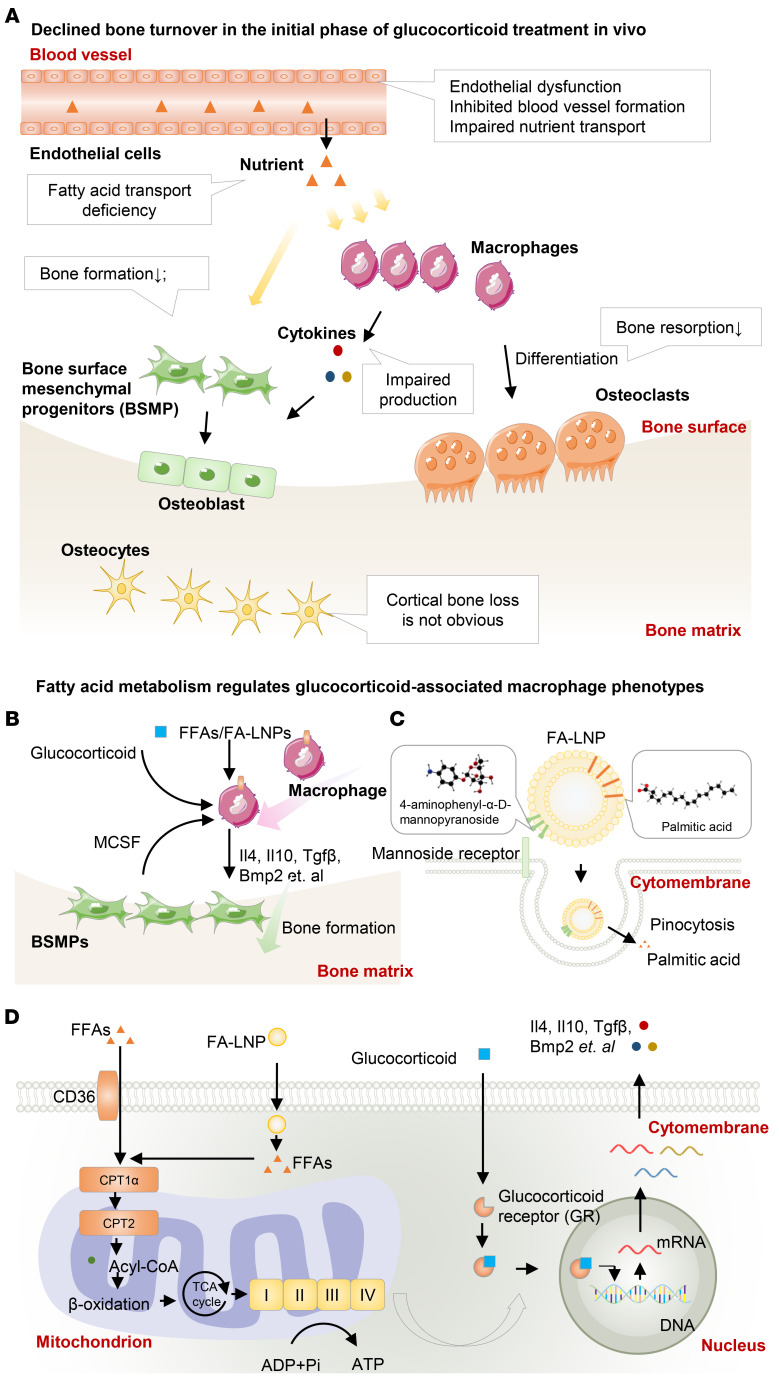
Schematic overview of the main findings. (**A**) Excess GC levels cause rapid bone loss and delayed fracture healing. The main feature is reduced bone turnover. Disruption of vascularization impairs fatty acid delivery, leading to a simultaneous decrease in bone formation and resorption, but with a net bone loss. (**B**) Bone formation–associated macrophages are more likely to take up exogenous fatty acids. Fatty acid oxidation regulates the secretory phenotypes of M2c macrophages and promotes osteogenesis. (**C**) We developed a macrophage-targeted fatty acid delivery system (FA-LNPs) to increase delivery efficiency while reducing the frequency of local injections. (**D**) After entering the cytoplasm of the macrophage, fatty acids participate in mitochondrial oxidative phosphorylation, which fuels GC receptors and drives them into the nucleus to regulate the production of cytokines.

The etiology of GC-associated skeletal disorders is multifactorial, while the systemic effects regulating this process are less clear. Here, using established bone loss and fracture models, we show that excess GC exposure leads to reduced bone turnover, with a particular reduction in bone formation, while identifying the spatiotemporal progression and the multifaceted pathologies (Figure 12). Notably, mechanistically there are defects in the immunometabolism that result in altered osteogenesis of bone-forming cells. Accordingly, we synthesized nanoparticle-encapsulated lipids to reprogram the skeletal macrophages and thus improved fracture healing. Together, the present study provides new insights into the underlying etiology of excess GC-induced bone loss, allowing for potential new avenues in developing targeted therapy for this medical condition.

According to a dose conversion guide (45), the mouse doses of 2.1 mg/kg/d and 6.25 mg/kg/d of prednisolone are approximately equivalent to human doses of 10 mg/d and 30 mg/d, respectively (based on a 60 kg body weight). These doses correspond to moderate intensity in clinical treatment (low intensity < 7.5 mg/d; between 7.5 mg and 30 mg is moderate intensity; between 30 mg and 100 mg is high intensity; and exceeding 250 mg is regarded as pulse therapy) (46). The effect of GC-induced bone loss in mice is influenced by factors such as the formulation of the GC, the mouse strain, age, sex, and route of administration. Therefore, the optimal safe dose for mouse models remains to be further explored. Different formulations have varying potencies (e.g., Dex > methylprednisolone > prednisolone/prednisone > hydrocortisone/cortisone) (25, 47), but their mechanisms of action are consistent. We chose the subcutaneous implantation of a sustained-release prednisolone pellet as the induction method, which ensures stable GC delivery and reduces the need for repeated injections, minimizing stress on the animals. Furthermore, the literature suggests that FVB/N mice are more sensitive to GC-induced bone loss than Swiss Webster and C57BL/6 mice (48). Similarly, in humans, the safe dosage and intervention thresholds for GC use vary across different national guidelines. Generally, all patients starting or requiring long-term use of GCs are advised to improve nutrition, prevent falls, and consider lifestyle interventions (7).

Excess use of GCs causes both rapid and sustained loss of trabecular bone mass (7, 12). We identified reduced bone turnover as the primary feature of the rapid bone loss, consistent with clinical studies that showed a virtual lack of osteoblastic activity with reduced or unchanged bone resorption occurring during the rapid bone loss phase (11, 12). Previously, the spatiotemporal patterns of GC-associated fracture healing were less investigated. In this study, we found that during the early stage of callus formation (week 2 post-fracture), excess use of GCs amplified the inhibition of bone formation and resorption. In the later stages (4 and 8 weeks post-fracture), the delayed remodeling led to a relatively larger callus but with insufficient quality. The bone loss and fracture models used in this study were given GCs by continuous or once-daily dosing regimens, and the results consistently supported the conclusion that GCs reduce bone turnover in vivo. Thus, the choice of dosing regimen is not a notable factor influencing the main findings under the experimental conditions of this study.

The exogenous actions of GCs on osteogenesis have been suggested to be stimulatory (29, 49–51). The discrepancy between in vivo and in vitro results regarding the effects of GCs on bone formation (i.e., inhibition in vivo and promotion in vitro) urged us to explore a role for cellular crosstalk in vivo that was not replicated in vitro. In the present study, we addressed the regulation between macrophages and osteogenic progenitor cells, as inspired by previous studies (52, 53). Firstly, we detected a large proportion of macrophages (the major hematopoietic lineage) during rapid callus formation. Secondly, we found that Csf1, a well-established cytokine that regulates macrophage survival, is mainly produced by the bone-forming cells. Thirdly, we showed that macrophages are the primary target cells for GC-mediated immune regulation. Together, our data show that GCs and macrophages cooperate to promote osteogenic differentiation, ruling out immune regulation of macrophages as the cause of the discrepancy between in vivo and in vitro results.

We confirmed that excess GCs inhibited vascularization in vitro and in vivo. In the process of fracture healing, blocking of blood vessel ingrowth impedes vascularization and reduces local nutrient delivery, ultimately resulting in smaller bony callus (24). In an ex vivo model, collagen gel was used to simulate avascular tissue, and the permeation of nutrients was evaluated. Fatty acids exhibited weaker permeation compared with glucose, suggesting that fatty acids may be a limiting nutrient following vascularization imbalance (24). We speculated that fatty acid deficiency may be the culprit for the reduced bone turnover induced by excess GCs. Firstly, fatty acid levels rather than glucose are reduced in the bone marrow and callus after GC exposure. Consistent with previous studies showing that the skeleton is the second organ exerting high demand on circulating fatty acids (36, 54), our study proved that fatty acid oxidation is a dominant fuel source for cellular homeostasis of osteogenic precursors. In addition, we found that fatty acid deficiency leads to inhibition of both osteogenesis and osteoclastogenesis of progenitor cells. In addition to causing vascular defects, GCs can impact systemic lipid metabolism and, consequently, affect fatty acid transport. For instance, prior studies suggest that short-term GC exposure can promote lipolysis (55, 56), while chronic use does not result in further lipolysis (57, 58). Changes in weight and appetite can also exert an influence on fatty acid transport. Furthermore, the saturation and chain length of fatty acids may exert differing degrees of influence on bone turnover. These intricate mechanisms warrant further studies.

Our study also provides a framework for the nutritional distribution of fatty acids among bone units. Exogenous fatty acids after direct delivery are preferentially taken up by macrophages, not bone-forming cells, implying the demand on an effective delivery system targeting bone-forming cells. Fatty acid delivery shapes macrophage Mc2 polarization, which further supports osteogenesis. We demonstrated how fatty acids affect GC signaling to produce BMP2 — an example of cytokines. Interestingly, FCCP and oligomycin exhibit contrasting effects on the production of BMP2 and IL-6. It is noteworthy that both FCCP and oligomycin inhibit the function of complex V, but the regulation of BMP and IL-6 expression may not be solely attributed to the loss of mitochondrial ATP production. Furthermore, oligomycin hyperpolarizes the mitochondrial membrane, while FCCP depolarizes it, suggesting that the production of BMP2 and IL-6 may be also influenced by changes in mitochondrial membrane polarization. Notably, the pro-osteogenic effect of Mc2 macrophages is regulated by multiple cytokines. For example, fatty acid oxidation fuels the production of BMP7, IL-4, and IL-10 (41, 59, 60), but whether fatty acid oxidation facilitates the binding of GR to the promoters of these genes and their exact roles in osteogenesis are still elusive. We did show that targeting skeletal macrophages via nanoparticle-delivered fatty acids effectively promoted callus formation in vivo.

Energy metabolic patterns on pathological progression of bone loss and fracture models in mice are reported here. Given that the bone biology of mice has a striking similarity with that of humans (61, 62), we expect similar changes on bone turnover upon GC exposure in human cases, warranting future study. In addition, future target therapy should also be developed to completely reconstruct the nutrient transport network to restore the energy metabolism levels of various cells.

Excessive exogenous GCs may lead to adrenal atrophy and suppression of the hypothalamic-pituitary-adrenal axis, resulting in reduced endogenous GC levels (63). Our study also detected a decrease in endogenous GC levels during the exposure of exogenous GCs (results not shown). It remains an important question for future research to explore whether bone remodeling abnormalities can be restored after discontinuation of GC treatment and to investigate the effects of changes in endogenous GC levels on bone turnover.

In conclusion, reduced bone turnover and bone formation is the primary feature of both rapid bone loss and delayed bone healing under conditions of excess GC exposure. These effects are dependent on various processes, including direct actions on bone progenitor cells, but also on intercellular interactions between these cells and macrophages that become defective as a result of alterations in the vasculature, leading to nutrient deficiency and thus altered immunometabolic activity. Targeted repletion of the missing energy source rescued defects in bone healing. Thus, this study highlights a potential new therapeutic approach targeting immunometabolism of the skeletal microenvironment that may have clinical significance for tackling the side effects of excess GC exposure on the skeletons.

## Methods

### Sex as a biological variable.

Our study examined male and female animals, and similar findings are reported for both sexes.

### Mice.

C57BL/6J male and female mouse strains were provided by the Laboratory Animal Centre of the Chinese University of Hong Kong and Shenzhen Institute of Advanced Technology, Chinese Academy of Sciences.

The CD11b-DTR mouse strain was purchased from The Jackson Laboratory (JAX stock 006000). The Ubc-Cre-ERT2 mouse strain was purchased from The Jackson Laboratory (JAX stock 007001). The Cpt1a-Flox (NM-CKO-0056) mouse strain was purchased from Shanghai Model Organisms Center Inc. Ubc-Cre-ERT2 mice were crossed with Cpt1a-Flox mice. The offspring were intercrossed to generate *Ubc-Cre-ERT2 Cpt1a^fl/fl^* mice. Mice were housed in microisolator cages (18°C–23°C, 40%–60% humidity) under 12-hour light/12-hour dark cycles with free access to water and food according to experimental design.

Other methods and materials are presented in Supplemental Methods and Supplemental Tables 1 and 2.

### Statistics.

Mice were assigned to groups randomly. Experiments were done in a non-blinded fashion. Sample size was determined based on the pilot study of the bone loss model, which found that using 4 or more mice yielded *P* values less than 0.05 with a 90% probability of bone loss upon GC treatment for 4 weeks. All numerical data are reported as mean ± SD. Experiments were performed repeatedly. For the analysis of statistical significance, a 2-tailed Student’s *t* test or 1-way or 2-way ANOVA with Bonferroni’s post hoc test was used (information is provided in the figure legends). *P* values less than 0.05 were considered statistically significant. *P < 0.05, **P < 0.01, ***P < 0.001, ****P < 0.0001. Sample sizes (*n*) are provided in the figure legends.

### Study approval.

All animal operations were approved by the Chinese University of Hong Kong Animal Experiment Ethics Committee (19-069-MIS; 20-106-MIS; 21-152-MIS; 22-069-MIS; 22-070-MIS) and the Institutional Animal Care and Use Committee (IACUC) of the Shenzhen Institute of Advanced Technology, Chinese Academy of Sciences (SIAT-IACUC-230327-YGS-WXL-A2261).

### Data availability.

The Gene Expression Omnibus (GEO) accession number for the 10× Genomics scRNA-Seq is GSE261072. All supporting data are provided in the Supporting Data Values file.

## Author contributions

LQ, JX, and XL designed research studies. XL, JX, TL, BD, LC, YZ, SH, JG, SX, LZ, HY, YL, XH, ZY, WT, and DHKC conducted experiments. LQ, JX, YN, HL, and XW provided reagents. LQ, JX, and XL wrote the manuscript.

## Supplementary Material

Supplemental data

Unedited blot and gel images

Supporting data values

## Version 1 03/21/2024

In-Press Preview

## Version 2 05/15/2024

Electronic publication
