# Supplementary material for: Excess glucocorticoids inhibit murine bone turnover via modulating the immunometabolism of the skeletal microenvironment
Source: J Clin Invest. 2024 Mar 21;134(10):e166795. doi: 10.1172/JCI166795 (PMC11093612; doi:10.1172/JCI166795)
Supplement: Supplemental data [file jci-134-166795-s232.pdf]

Supplemental information

Excess glucocorticoids inhibit murine bone turnover via modulating the immunometabolism of the skeletal microenvironment

Supplementary information of methods

Genotyping

Genotyping was conducted by PCR. The primer sequences used for genotyping of CD11b-DTR transgene were 5'-GGGACCATGAAG CTGCTGCCG-3' (forward) and 5'-TCAGTGGGAATTAGTCATGCC-3' (reverse). The primer sequences used for genotyping of Ubc-cre-ERT2 transgene were 5'-GCG GTCTGGCAGTAAAAACTATC-3' (forward) and 5'-GTGAAACAGCATTGCTGTCACTT-3' (reverse). The primer sequences used for genotyping of Cpt1a-Flox transgene were 5'-GCAAGGGTAGTTTGGGAGC-3' (forward) and 5'-TCAGGCAGCAGGGAGAAG-3' (reverse).

Glucocorticoid-induced bone loss model

20-week-old C57BL/6J mice (of both genders) were used. All the mice were anesthetized, and prednisolone slow-release pellets (or placebo control) were applied by subcutaneous implantation (2.1 mg/kg/d and 6.25 mg/kg/d for male; 6.25 mg/kg/d for female) based on the protocol established in previous study (1). Serum CTX and PINA were assayed with mouse CTX-1 ELISA Kit (Abclonal) and mouse PINP / N-terminal propeptide of Collagen alpha-1(I) chain ELISA Kit (Abclonal) according to the manufacturer's protocols. Mice were euthanized and samples were collected at 1, 2, 3, and 4 weeks (afternoon session, 2:00pm-3:00pm) for further analysis.

Glucocorticoid-associated fracture healing model

The mice were injected daily intraperitoneally (IP) with prednisolone at a dose of 6.25 mg/kg/d (or the vehicle as control) for two consecutive weeks. Closed femur fractures were created on both prednisolone- and vehicle-injected mice as described previously (2). Anesthetized mice were prepared for surgery by shaving the fur around the knee area and disinfecting the skin. An incision was made to dislocate the patella, and the intramedullary canal was accessed by creating a hole in the distal end of the femur. The femur fracture was made by cutting the shaft with a wire saw. Then the femur was stabilized with a 21-gauge stainless steel intramedullary needle, and the wound was sutured and closed. Daily intraperitoneal (IP) injections of prednisolone (or the vehicle as control) were continued until the mice were killed for analysis. Due to significant fluctuations (increase) of endogenous glucocorticoid levels in mice during light-on and light-off transitions, while remaining relatively stable (low level) during the daytime (3, 4), the prednisolone treatment was administered around noon session and all the mouse samples were collected during a unified afternoon session (2:00pm-3:00pm).

For systemic macrophage depletion experiments, DT (25 ng/g i.p.) was injected every other day (saline as control) of CD11b-DTR mice from day 7 to 14 post-fracture during callus formation. For local macrophage depletion experiments, clodronate liposomes were injected daily at the fracture site of C57BL/6J male mice (PBS liposomes as control).

For macrophage colony induction, Csf1 (1.5 μg/day) was injected daily at the fracture site of C57BL/6J male mice from day 7 to 14 post-fracture. To evaluate the role of fatty acids in fracture healing, 20 μL corn oil (Sigma) was injected daily at the fracture site of C57BL/6J male mice from day 7 to 14, as previously described (5). Control group received saline injection without further specification.

For systemic inhibition of fatty acid oxidation, tamoxifen (0.15mg/g i.p.) was injected every other day to *Ubc-Cre-ERT2;Cpt1a*$^{fl/fl}$ mice from day 7 to 14 post-fracture to knockout the gene encoding carnitine palmitoyltransferase-1a (Cpt1a).

For systemic fatty acid delivery, C57BL/6J male mice were fed with a high-fat diet (45%) (Research Diets INC.; D12451) or a normal chow diet from 1-week post-surgery. For FA-LNPs delivery, 30 μL of FA-LNPs or saline was injected locally at the fracture site of C57BL/6J male mice every other day from day 7 to 14 post-fracture.

X-ray and Dual-energy X-ray absorptiometry (DXA) imaging

For the glucocorticoid-induced bone loss model, mice were anesthetized, and DXA imaging was performed at one, two, and four weeks. The whole body, femur, tibia, and spine were measured. The analysis parameters included BMD, BMC (Bone mineral content), and soft tissue weight. For the glucocorticoid-associated fracture healing model, mice were anesthetized, and X-ray imaging was performed weekly to monitor the changes in the callus. Callus length, width, and area were measured.

Micro-computed tomography (micro-CT) imaging

Bone samples were isolated and fixed overnight in 4% paraformaldehyde (PFA), then immersed in 70% ethanol before micro-CT scanning. In the glucocorticoid-induced bone loss model, the tibia and femur of mice were scanned for the measurement of trabecular and cortical bone, respectively. The high-resolution μCT40 (Scanco Medical, Brüttisellen, Switzerland) was used. The spatial resolution was 15 μm. 1 mm depths of trabecular bone under the growth plate of the proximal tibia (100 slices) were measured. Parameters were analyzed, including BV, TV, BMD, BV/TV, Tb.Th, Tb.Sp, and Tb.N.

Quantitative measurements were conducted at Gauss Sigma 0.8, Gauss Support 1, Lower Threshold 220, Upper Threshold 1000.

In the glucocorticoid-associated fracture healing model, the intramedullary needle was removed after separating the fractured femur, and the muscle tissue was dissected. At week 2 after surgery, calluses were analyzed by selecting 100 sections at both distal ends of the calluses. The average value of parameters at both ends was selected for statistical analysis. The middle region was also analyzed by selecting 100 sections from the center of the fracture site. At week 4 and 8 after surgery, 150 slices in both directions of the fracture gap (a total of 300 slides) were selected for analysis. Parameters were analyzed, including BV, BMD, and BV/TV. Quantitative measurements were conducted at Gauss Sigma 0.8, Gauss Support 1, Lower Threshold 220, Upper Threshold 1000.

For the Micro-CT-based angiography, as previously reported (6), mice were killed, and the thoracic cavity was opened at week 2 after surgery. Saline containing heparin sodium (100 U/ml) was injected to flush the vasculature from the left ventricle, and 4% PFA was injected to fix the specimens. MicroFil® (MV-122, Flow-Tech; Carver, MA, USA) was injected to fill the vasculature. After perfusion, samples were kept at 4°C for 24 hours; then, fractured femora were decalcified in 10% EDTA-PBS solution (pH 7.2) for 14 days. The vasculature was scanned and visualized using the high-resolution μCT40 (Scanco Medical, Brüttisellen, Switzerland), and the spatial resolution was 15 μm. Quantitative measurements were conducted at Gauss Sigma 0.8, Gauss Support 1, Lower Threshold 150, Upper Threshold 1000.

Mechanical strength testing

At week 4 post-fracture, soft tissues were removed thoroughly, and the femur was used for mechanical

strength testing. Four-point Bending Test was employed for the analysis of structural properties of hard callus by using a mechanical testing machine (H25KS Hounsfield Test Equipment, Redhill, Surrey, UK). The upper and lower spans were set to 8 mm and 16 mm, respectively. The load was applied at a rate of 0.005 mm/s until failure to test the maximum load (N).

Dynamic histomorphometric analysis

For the glucocorticoid-induced bone loss model, mice were IP injected with 5 mg/kg calcein green (first injection) and 90 mg/kg xylenol orange (second injection) at 7 and 3 days, respectively, before they were euthanized for analysis, as previously described (7). Non-decalcified tibiae were collected for methylmethacrylate (MMA) embedding for sectioning (thickness of 5 μm). Under a fluorescence microscope (ZEISS AxioPlan2, Germany), the area below the tibial growth plate was selected as a region of interest for assessing the bone formation rate. Sections were also stained with Goldner Trichrome staining for quantification of bone surface osteoblasts and osteoclasts.

For the glucocorticoid-associated fracture healing model, mice were intraperitoneally injected with 5 mg/kg calcein green (first injection) and 90 mg/kg xylenol orange (second injection) at 10 and 3 days, respectively, before sacrificed for analysis. The non-decalcified femur was collected for MMA embedment and sectioned into 5μm sections. Under a fluorescence microscope (ZEISS AxioPlan2, Germany), the area of hard callus was selected as a region of interest for assessing bone formation rate. Sections were also stained with Goldner Trichrome staining to visualize callus cells.

Histology

The fixed femora or tibiae were decalcified in 10% EDTA-PBS solution (pH 7.2) for two weeks. The

metatarsals were dissected from E16.5 embryos and were fixed in 4% PFA. The fixed metatarsals were decalcified in 10% EDTA-PBS solution (pH 7.2) for one week. During the period, the EDTA-PBS solution was replaced every three days. Then, the decalcified tissue was dehydrated, embedded in paraffin, and sectioned into 5 μm sections. The sections were stained with H&E, Sirius red, and Safranin O & Fast green. Images were taken by a fluorescence microscope (ZEISS AxioPlan2, Germany). ImageJ quantified the woven bone area, percentage of woven bone area, cartilage area, percentage of cartilage area, the ratio of bone to cartilage area, and total callus area.

For immunohistochemistry staining, paraffin sections, after deparaffinization and rehydration, were incubated in antigenic retrieval solution at 100°C for 5 minutes and incubated in 3% hydrogen peroxide at room temperature for 15 minutes. Then, sections were permeabilized with 0.5% Triton-X100 in PBS and blocked with PBS blocking solution (containing 5% BSA). Next, sections were incubated with primary antibodies (Anti-Sp7, Abcam, 1:200; Anti-Glut1, Abclonal, 1:200; Anti-Cpt1a, Abclonal, 1:200; Anti-IL1, Abclonal, 1:200; Anti-IL6, Abcam, 1:200; Anti-TNFα, Abcam, 1:200; IL10, Abclonal, 1:200; Inos, Santa Cruz, 1:100; Arg1, Abclonal, 1:200; Cd31, R&D system, 10μg/m; F4/80, Abcam, 1:200) at 4°C overnight. After washing with PBS, sections were incubated with secondary antibodies (Goat Anti-Mouse IgG H&L, Abcam, 1:400; Goat Anti-Rabbit IgG H&L, Abcam, 1:400; Donkey Anti-Goat IgG H&L, Abcam, 1:400) for 2 hours, washed, mounted with DAPI, and visualized under a fluorescence microscope (ZEISS AxioPlan2, Germany).

Measurements of fatty acid and glucose

Mice serum fatty acids were assayed with a Free Fatty Acid Assay Kit (Abcam) according to the manufacturer's protocol. Mice serum glucose was assayed with a Glucose Assay Kit (Beyotime) with

O-toluidin, according to the manufacturer's protocol.

For the measurement of marrow fatty acid and glucose, unilateral femur and tibia were harvested, and both ends of bone tissue outside the growth plate were removed. The remaining bone tissue was inserted into 1.5 mL microcentrifuge tubes. Bone marrow was separated from tubular bone by centrifugation (13,000 × *g,* 9 seconds) and resuspended in 500 µl RBC Lysis Buffer (1X). After another centrifugation (500 × *g*, 3 minutes), the supernatant was collected for fatty acid and glucose measurement using the above assay kits.

For the measurement of local fatty acid and glucose, the callus along with the femur was harvested, and both ends of bone tissue outside the growth plate were removed. The remaining bone tissue was cut into pieces and inserted into 1.5 mL microcentrifuge tubes. Substrates were separated by centrifugation (13,000 × *g,* 9 seconds) and resuspended in a 500 µL 1X RBC Lysis Buffer. After another centrifugation (500 × *g*, 3 minutes), the supernatant was collected for fatty acid and glucose measurement using the above assay kits.

For the measurement of fatty acid from FBS (Gibco®), metabolites extraction was abstained for Gas Chromatography-Mass Spectrometer (GC-MS) Analysis in Biotree Company (Shanghai) according to the manufacturer's protocol. The concentrations of fatty acids were provided in Table S2.

Isolation of primary cells

Central bone marrow mesenchymal progenitors (CMPs) were isolated from the long bones of four to six-week-old C67BL/6J mice. Briefly, after the bilateral femora and tibiae were dissected under sterile conditions, bone marrow was flushed out of the bone using a 23-gauge needle and passed through a cell strainer (Falcon® 70 µm). The flushed cells were cultured in an α-MEM (Gibco®) culture medium

supplied with 10% FBS (Gibco®) and 1% Penicillin-Streptomycin-Neomycin Antibiotic Mixture (PSN, Gibco®) in a 37 °C incubator with 5% CO_2. After cells were adherent to the wall, clodronate liposomes (1 µL liposome to 1 mL medium) were added to eliminate phagocytes (mainly macrophages and osteoclasts lineages) for 6 hours. Cells (tested without mycoplasma infections) from passages 1 to 3 were used for the experiments.

Bone surface mesenchymal progenitors (BSMPs) were isolated from the long bones of four to six-week-old C67BL/6J male mice. Briefly, after the bilateral femora and tibiae were dissected under sterile conditions, bone marrow was flushed out of the bone using a 23-gauge needle. Both ends of bone tissue outside the growth plate (mainly subchondral bone and cartilage) were removed. Then, the bone tissue was longitudinally cut into two halves, as previously described (8), and digested with a collagenase A solution (2 mg/ml) for 60 minutes. Next, cells within the supernatant were passed through a cell strainer (Falcon® 70 µm), washed, and cultured in an α-MEM (Gibco®) culture medium supplied with 10% FBS (Gibco®) and 1% Penicillin-Streptomycin-Neomycin Antibiotic Mixture (PSN, Gibco®) in a 37 °C incubator with 5% CO_2. After cells were adherent to the wall, clodronate liposomes (1 µL liposome to 1 mL medium) were added to eliminate phagocytes (mainly macrophages and osteoclasts lineages) for 6 hours. Cells from passages 1 to 3 were used for the experiments.

Bone marrow macrophages were isolated from the long bones of 4–6-week-old C67BL/6J male mice. Briefly, after the bilateral femora and tibiae were dissected under sterile conditions, bone marrow was flushed out of the bone using a 23-gauge needle and passed through a cell strainer (Falcon® 70 µm). Red cells were removed by using 1X RBC Lysis Buffer (Invitrogen, no. 00-4333) and cultured in an α-MEM (Gibco®) culture medium supplied with 10% FBS (Gibco®) and 1% Penicillin-Streptomycin-Neomycin Antibiotic Mixture (PSN, Gibco®) in a 37 °C incubator with 5% CO_2 for 4-

6 hours. Supernatants were collected, centrifuged, resuspended, and cultured in an α-MEM (Gibco®) culture medium supplied with 10% FBS (Gibco®), 1% Penicillin-Streptomycin-Neomycin Antibiotic Mixture (PSN, Gibco®) and 30 ng/mL Csf-1 (MCE) in a 37 °C incubator with 5% CO_2.

Bone surface cells were isolated from C67BL/6J male mice for *ex vivo* culture after two weeks of implantation of prednisolone or placebo control pellets, as adapted from a previously described protocol (3). Briefly, after the bilateral femora and tibiae were dissected under sterile conditions, bone marrow was flushed out of the bone using a 23-gauge needle. Both ends of bone tissue outside the growth plate (mainly subchondral bone and cartilage) were removed. Next, periosteum and periosteal progenitors were stripped using sharp forceps and digested with a protease solution (2 mg/ml collagenase A and 2.5 mg/ml trypsin) for 20 minutes to obliterate periosteal compositions. Then, the bone tissue was longitudinally cut into two halves and digested with a collagenase A solution (2 mg/ml) for 60 minutes. Next, cells within the supernatant were passed through a cell strainer (Falcon® 70 μm), washed, and cultured in an α-MEM (Gibco®) culture medium supplied with 10% FBS (Gibco®) and 1% Penicillin-Streptomycin-Neomycin Antibiotic Mixture (PSN, Gibco®) in a 37 °C incubator with 5% CO_2. After cells were adherent to the wall, osteogenic induction medium (αMEM, 1% PSN, 10% FBS, 10 nM Dex, 10 mM sodium β-glycerophosphate, and 0.05 mM ascorbic acid-2-phosphate) or osteoclastogenic induction medium (αMEM, 1% PSN, 10% FBS, recombinant mouse M-CSF (30ng/ml; Thermo Fisher) and Rankl (30 ng/ml; R&D systems)) were replaced. Five days after osteogenic or osteoclastogenic induction, ALP or TRAP staining was performed according to the manufacturer's guidelines.

Callus cells were isolated from C67BL/6J male mice at week 2 post-fracture surgery. Briefly, the callus was dissected from fractured femora under sterile conditions. Then, callus was minced and

digested with a collagenase A solution (2 mg/ml) for 1 hour. Next, 10% FBS were added to the collagenase A solution. After another 2 hours of digestion, cells within the supernatant were passed through a cell strainer (Falcon® 70 μm), washed, and cultured in an α-MEM (Gibco®) culture medium supplied with 10% FBS (Gibco®) and 1% Penicillin-Streptomycin-Neomycin Antibiotic Mixture (PSN, Gibco®) in a 37 °C incubator with 5% CO_2. After cells were adherent to the wall, osteogenic induction medium (mentioned above) or osteoclastogenic induction medium (as described above) were replaced. Five days after osteogenic or osteoclastogenic induction, ALP or TRAP staining (Sigma) was performed according to the manufacturer's guidelines.

10X Genomics single-cell RNA-seq and data analysis

Bone surface and callus cells were isolated freshly for 10X Genomics single-cell RNA-seq. Cells were lysed using red cell lysis buffer (Gibco®) prior to sequencing. Cells from 5 mice in each group were pooled together before quality control, reverse transcription, and library construction using Chromium Single Cell 3' Reagent Kits. Samples (3000 to 6000 cells in each group) were sequenced on the mgiseq2000 platform yielding an average of 40,000-70,000 reads per cell and a median of 1300-1700 genes per cell. Cells expressed genes <200, and mitochondrial DNA >15% were excluded.

All sequencing data were loaded using 'Seurat 4.1.0' in 'Rstudio 2021.09.0' and proceeded with quality control. Cells with too few or too many counts/features or cells highly express mitochondrial genes were all removed. Count data were normalized and scaled using SCTransform. The cell cycle effect was removed by using Cell Cycle Scoring. The top 3000 variable genes were selected for PCA analysis. Canonical correlation analysis (CCA) was performed to integrate data from different groups. Clusters were identified by FindClusters, using the top 30 principal components at a resolution of 0.5.

The UMAP algorithm was applied to visualize cells in a two-dimensional space. The top 10 conserved markers for each cell cluster were identified by Find Conserved Markers based on the Wilcoxon Rank Sum test with default parameters (min. pct = 0.1, logfc. threshold = 0.25). Each cluster's specific cell type was then confirmed using 'SingleR 1.9.2' and verified with the mouse cell atlas database (MCA2.0). The Gene Ontology (GO) and Kyoto Encyclopedia of Genes and Genomes (KEGG) analyses were performed by 'clusterProfiler 4.2.2'. Pathways with a p_adj value less than 0.05 were considered significantly enriched. 'velocyto.py 0.17.17' and 'velocyto.R 0.6' were used to perform RNA velocity analyses. 'monocle3 1.0.0' was used to perform trajectory analysis and depict a pseudotime diagram. The cell-cell communication was analyzed by using CellChat (version 1.5.0).

Cell treatment

Direct co-culture and transwell cell migration experiments were performed for the assay of macrophages recruitment by bone surface mesenchymal progenitors. For the direct co-culture experiments, bone surface mesenchymal progenitors (transfected with or without *Csf1* siRNA) were cultured in a 24-well plate, and cell suspension containing 10^5 Dil labeled macrophages was added. At the end of the culture, cells were washed with PBS to remove non-adherent cells. The number of Dil positive adherent cells was quantified. For the transwell cell migration experiments, bone surface mesenchymal progenitors (transfected with or without *Csf1* siRNA; pretreated with vehicle or Dex) were seeded onto the surface of the lower chamber with 600 μl complete αMEM medium and macrophages were planted in the upper chamber in 100 μl of serum-free αMEM medium. After culture for 24 hours, cells (after the migration through the filter) on the lower surface of the member were fixed by 4% PFA, stained with 0.1% crystal violet solution, and photographed.

For osteogenic differentiation induction, bone marrow adherent cells or bone surface mesenchymal progenitors were cultured in an osteogenic induction medium (including αMEM, 1% PSN, 10% FBS, 10 mM sodium β-glycerophosphate, and 0.05 mM ascorbic acid-2-phosphate) in the presence or absence of Dex. For the osteogenic induction with the addition of macrophages, BSMPs or CMPs were mixed with macrophages at a ratio of 1:5 before seeding on the surface of 24 well plates and cultured in an osteogenic induction medium (including αMEM, 1% PSN, 10% FBS, 10 mM sodium β-glycerophosphate, and 0.05 mM ascorbic acid-2-phosphate) in the presence or absence of Dex. Differentiation capacity was evaluated by q-RT PCR, ALP, or ARS staining at indicated time points.

For osteoclastogenic differentiation induction, bone marrow macrophages were cultured in an osteoclastogenic induction medium (as described above) in the presence or absence of Dex. Differentiation capacity was evaluated by TRAP staining at indicated time points.

For nutrient-deprivation assays, bone marrow adherent cells or bone surface mesenchymal progenitors were cultured in the presence or absence of bone marrow macrophages. Nest, cells were induced for osteogenic differentiation under culture of control medium (αMEM, 1% PSN, 10% FBS, 10 mM sodium β-glycerophosphate, 0.05 mM ascorbic acid-2-phosphate, 5 mM D -(+) glucose, 2 mM L-glutamine, 1 mM sodium pyruvate), low serum medium (αMEM, 1% PSN, 1% FBS, 10 mM sodium β-glycerophosphate, 0.05 mM ascorbic acid-2-phosphate, 5 mM D -(+) glucose, 2 mM L-glutamine, 1 mM sodium pyruvate), low glucose medium (αMEM, 1% PSN, 10% FBS, 10 mM sodium β-glycerophosphate, 0.05 mM ascorbic acid-2-phosphate, 0.5 mM D -(+) glucose, 2 mM L-glutamine, 1 mM sodium pyruvate) or low glutamine medium (αMEM, 1% PSN, 10% FBS, 10 mM sodium β-glycerophosphate, 0.05 mM ascorbic acid-2-phosphate, 5 mM D -(+) glucose, 0.2 mM L-glutamine, 1

mM sodium pyruvate) in the presence or absence of Dex. Differentiation capacity was evaluated by ALP or alizarin red staining at indicated time points. Bone marrow macrophages were induced for osteoclastogenic differentiation under culture of control medium (αMEM, 1% PSN, 30 ng/ml RANKL, 30ng/ml MCSF, 10% FBS, 5 mM D -(+) glucose, 2 mM L-glutamine, 1 mM sodium pyruvate), low serum medium (αMEM, 1%PSN, 30 ng/ml RANKL, 30 ng/ml MCSF, 1% FBS, 5 mM D -(+) glucose, 2 mM L-glutamine, 1 mM sodium pyruvate), low glucose medium (αMEM, 1% PSN, 30 ng/ml RANKL, 30ng/ml MCSF, 10% FBS, 0.5 mM D -(+) glucose, 2 mM L-glutamine, 1 mM sodium pyruvate) or low glutamine medium (αMEM, 1%PSN, 30 ng/ml RANKL, 30 ng/ml MCSF, 10% FBS, 5 mM D -(+) glucose, 0.2 mM L-glutamine, 1 mM sodium pyruvate) in the presence or absence of Dex. Differentiation capacity was evaluated by TRAP staining at indicated time points.

For interventions targeting fatty acid metabolism, bone marrow adherent cells or bone surface mesenchymal progenitors were cultured in the presence or absence of bone marrow macrophages. Next, cells were induced for osteogenic differentiation culturing with control medium (as described above), low serum medium (as described above), and in the presence or absence of Dex, oleate acid, palmitate acid, GW9508, and FA-LNPs. For the knockdown of *Cpt1a* and *Atgl*, cells were transfected with vehicle, *Cpt1a* shRNA or *Atgl* shRNA by Lipofectamine 3000, respectively, before osteogenic induction. Differentiation capacity was evaluated by ALP or ARS staining at indicated time points.

Bone marrow macrophages were induced for osteoclastogenic differentiation by culturing with control medium (as described above) and different serum conditions as described above. Additionally, macrophages were transfected with vehicle, *Cpt1a* shRNA or *Atgl* shRNA before osteoclastogenic induction. Differentiation capacity was evaluated by TRAP staining at indicated time points.

For the evaluation of fatty acid metabolism on macrophages phenotypes, macrophages were

cultured in control medium (as described above) or low serum medium (as described above) in the presence or absence of Dex, oleate acid, palmitate acid, GW9508, SSO, oligomycin, antimycin, and FA-LNPs. Additionally, cells were transfected with vehicle, *Cpt1a* shRNA or *Atgl* shRNA by Lipofectamine 3000. After the culture for 24 hours, cells were used for RT–qPCR analysis, and cellular supernatant was used for BMP2 (Abcam) or IL-6 (Abclonal) ELISA analysis, according to the manufacturer's protocol.

For the evaluation of macrophage phenotypes on osteogenesis, macrophages were cultured in the presence or absence of low serum, Dex, palmitic acid, *BMP2* siRNA, and SSO for one day, then cultured in low serum for another day. Cellular supernatants were collected and prepared as osteogenic induction medium by adding 10% FBS, 50 μg/ml ascorbic acid, and 10 mM β-glycerophosphate. BSMPs were treated with these cellular supernatants for osteogenic induction. Differentiation capacity was evaluated by ALP or ARS staining at indicated time points.

Cell viability assay by cell counting kit-8 (CCK8)

To assess the cell viability of BSMPs, we cultured them in two different media: control medium (as described above) and low serum medium (as described above). The cultures were supplemented with Dex, oleate acid, palmitate acid, GW9508, SSO, oligomycin, antimycin, and FA-LNPs. Additionally, for the knockdown of *Cpt1a* and *Atgl*, cells were transfected with mock-vehicle, *Cpt1a* shRNA or *Atgl* shRNA using Lipofectamine 3000. After culture for 24 hours, we determined cell viability using CCK8, following the manufacturer's protocol.

To investigate cell viability of BSMPs in the presence of macrophages, we employed a transwell culture system. The BSMPs were cultured in the lower chamber, while macrophages were cultured in

the upper chambers. Following the aforementioned culture conditions, we removed the upper chambers and assessed the cell viability of BSMPs using CCK8.

Cell lines

The MS-1 endothelial cell line was used and cultured in an α-MEM (Gibco®) culture medium supplemented with 10% FBS (Gibco®) and 1% Penicillin-Streptomycin-Neomycin Antibiotic Mixture (PSN, Gibco®) in a 37 °C incubator with 5% CO_2.

Tube formation assay

1×10^5 MS-1 endothelial cells were seeded in a Matrigel-coated 24-well plate in the presence of a different dose of Dex. Images were captured after 6 hours of exposure to assay the number of branches and total branch lengths.

Scratch test

MS-1 endothelial cells were seeded in 6 well plates in an α-MEM culture medium. When cells reached 80% confluence, serum starvation α-MEM culture medium was replaced for 12 hours of culture. A sterile p20 tip was used to create scratches following a PBS wash to remove detached cells. Serum starvation α-MEM culture medium was added in the presence of different doses of Dex. The wound images were captured at 0 and 24 hours, and wound areas were calculated.

Endothelial permeability assay

The endothelial permeability assay was conducted as described (9). MS-1 endothelial cells were

seeded in the upper chamber of collagen (15 mg/ml) and fibronectin (30 mg/L) coated transwell polyester membranes (0.4μm pore). When cells reach confluence, 500 ml α-MEM culture medium containing 4.4 kDa TRITC-dextran (2 mg/ml) or 70 kDa FITC-dextran (2 mg/ml) (Sigma-Aldrich) were added to the upper chamber. At 1-6 hours, 30 μl medium was collected from the lower chamber to measure fluorescence intensity.

Cellular nitric oxide and ROS level measurements

Nitric oxide measurement was conducted using a DAF-2 DA probe. MS-1 endothelial cells were seeded in 96 well black plates in α-MEM culture medium in different doses of Dex. At 24 hours, the DAF-2 DA probe was added to a final concentration of 5 μM following a 20 minutes incubation at 37°C. Then, cells were washed with PBS before the measurement of fluorescence intensity.

ROS assay was conducted by using an H2DCFDA (DCFH-DA) probe. MS-1 endothelial cells were seeded in 96 well black plates in α-MEM culture medium in different doses of Dex. At 24 hours, serum-free α-MEM culture dissolved H2DCFDA (DCFH-DA) (10 μM) probe was added following a 20 minutes incubation at 37°C. Then, cells were washed with PBS before the measurement of fluorescence intensity.

Immunofluorescent staining in cells

Cells (i.e., MS-1 endothelial cells and macrophages) were seeded on coverslips. MS-1 endothelial cells and macrophages, after one day's specific treatment, were fixed with 4% PFA, permeabilized with 0.5% Triton-X100 in PBS, and blocked with PBS blocking solution (containing 5% BSA). Next, coverslips were incubated with primary antibodies (Anti-GR, Abclonal, 1:200; Anti-VE-Cadherin, Abclonal,

1:200; Anti-ZO-1, 1:200, Abclonal) at 4°C overnight. After washing with PBS, sections were incubated with secondary antibodies (Goat Anti-Rabbit IgG H&L, Abcam, 1:400) for 2 hours, washed, mounted with DAPI, and visualized under a fluorescence microscope (ZEISS AxioPlan2, Germany).

Flow cytometry

For cell cycle assay, MS-1 endothelial cells were harvested and fixed in 70% ethanol. Then, cells were washed, stained by propidium iodide (PI) (Biolegend) staining solution (with 0.2 mg/mL RNase A) following the manufacturer's instructions, and analyzed by using a BD FACS flow cytometer. For cell apoptosis assay, MS-1 endothelial cells were harvested and stained by Annexin V Apoptosis Detection Kit (eBioscience) following the manufacturer's instructions and analyzed by using a flow cytometer. For the analysis of Cd36 expression, macrophages or BSMPs were collected harvested and fixed in 70% ethanol. Then, cells were washed and incubated with antibody (Alexa Fluor® 488 anti-mouse CD36, Biolegend, 1:400). After washing with PBS, cells were harvested and analyzed by using a flow cytometer. For mitochondrial mass quantification, macrophages, after specific treatment, were added with mitotracker green solution (final working concentration 100 nM) in a culture medium and incubated for 40 minutes at 37°C. Then, cells were harvested and analyzed by using a flow cytometer. For glucose and fatty acids uptake assay, macrophages were incubated with the fluorescent fatty acid analog BODIPY 558/568 C12 (Red-C12; Thermo Fisher Scientific) (final working concentration 1μM) or fluorescent glucose analog, 2-NBDG (Thermo Fisher Scientific) (final working concentration 300μM) in culture medium for 24 hours. Then, cells were harvested and analyzed by using a flow cytometer. For FA-LNPs uptake assay, macrophages were incubated with Dil labeled FA-LNPs in a culture medium. Cells were harvested at specific time points and fixed in 70% ethanol before flow

cytometry analysis.

Cell metabolic analysis

Macrophages were seeded onto 96 well plates (about 20,000 cells/100μl medium/well) with the selective treatment of low serum (1% FBS), Dex, oleic acid (25 μM) and palmitic acid (25 μM). ATP concentrations were assayed after 24 hours of culture with Luminescent ATP Detection Assay Kit (Abcam) following the manufacturer’s protocol.

Real-time ATP rate assays were conducted using the Seahorse XF platform (Agilent, Seahorse Bioscience). The assays enabled the estimation of ATP synthesis rates from both glycolysis and mitochondria based on measurements of oxygen consumption and pH changes. Cells were seeded onto wells of XF96 plate for Dex exposure. Osteoclasts were induced for five days in the presence of MCSF (30 ng/ml) and RANKL (30 ng/ml). Macrophage and BSMPs were cultured in the presence or absence of Dex for one day before the assay with the XF96 Extracellular Flux Analyzer (Agilent, Seahorse Bioscience).

To assess the oxidation of fatty acids, glucose, and glutamine in macrophages and BSMPs, we utilized Seahorse XF Cell Mito Stress Test Kits, following the manufacturer's guidelines. Cells were seeded onto wells of the XF96 plate and transfected with vehicle, *Cpt1a* shRNA (to knock down fatty acid oxidation), *Mpc1* shRNA (to knock down glutamine oxidation), or *Gls* shRNA (to knock down glucose oxidation). Subsequently, the cells were cultured in the presence or absence of Dex for one day before conducting the assay using the XF96 Extracellular Flux Analyzer (Agilent, Seahorse Bioscience).

Plasmids and RNA interference

For shRNA experiments, oligonucleotides targeting mouse *Cpt1a* (5'-GCAAAGATCAATCGGACCCTA-3'), *Gls* (5'- AGAAAGTGGAGATCGAAATTT-3'), *Mpc1* (5'-ATCAGTGGGCGGATGACTTTC-3'), *Atgl* (5'- CATCTCCCTGACTCGTGTTTC-3') were annealed and cloned into the pSLenti-U6-shRNA-CMV-EGFP-F2A-Puro-WPRE vector (OBiO Scientific Service; Shanghai). Non-specific oligonucleotides (5′-CCTAAGGTTAAGTCGCCCTCG-3′) were used as non-silencing controls in all experiments.

Western blotting

Proteins were extracted from MS-1 endothelial cells by using RIPA buffer supplemented with a protease inhibitor cocktail (MCE). Proteins were separated by SDS–PAGE and transferred to a PVDF membrane. Membranes were blocked with 5% dry milk (in Tris-buffered saline with 0.1% Tween-20) and probed with anti-Nos3 (ABclonal, 1:1000) and anti-Gapdh (Invitrogen:1:5000). Then, western blot images were scanned after the reaction with HRP-conjugated secondary antibodies.

Total RNA extraction and RT–qPCR analysis

Total RNA was extracted using the TRIzol reagent (Invitrogen). According to the manufacturer's protocol, RNA was converted to cDNA using the PrimeScript RT Reagent Kit (Takara). Gene expression was assayed with TB Green Premix Ex Taq kit (Takara) on a Real-Time PCR System (Thermo Fisher). Data were normalized with the $\Delta\Delta C(t)$ method and presented as fold changes. Primers used are listed in **Table S2**.

Liposome synthesis

FA-LNPs were prepared by the lipid thin-film hydration method (10). L-α-phosphatidylcholine, Cholesterol, Dihexadecyl phosphate, and 4-aminophenyl α-D-mannopyranoside were dissolved in 10 mL chloroform in a molar ratio of 7 mM/2 mM/1 mM/1 mM. The palmitic acid was added to ensure a final concentration of 1.25 mM. The mixture was evaporated to obtain a thin film, followed by hydration using 0.9% saline solution. Then, the liposomes were obtained by extruding the solution through polycarbonate filters with a pore size of 100 nm. The particle size distribution (~100 nm) and zeta potential (~0) of liposomes (Supplemental Figure 18C) were measured by Zetasizer ZS90.

ChIP–qPCR

After specific treatment for 24 hours, macrophages or MS-1 endothelial cells were cross-linked with 1% (w/v) formaldehyde for 15 minutes and quenched with 125 mM glycine. Then cells were harvested and incubated in RIPA Lysis and Extraction Buffer (Thermo Scientific) on ice for 15 minutes. Then cells were sonicated, and the chromatin was sheared into 200-1000 bp fragments. Samples were centrifuged, and the supernatant was incubated with an anti-GR antibody (Ablonal, 1ug:500mL cell lysate) or the Anti IgG antibody (as control; Santa Cruz, 1μg:500mL cell lysate). After precipitation using Pierce Protein A/G Magnetic Beads, DNA was purified and analyzed using qPCR. Specific primers for the *BMP2* promoter region (5′- ATTAAACAAGATTTGATGGCTGTCT-3′ (forward), 5′-TGGGTGGAGTGATTTGTAGGC-3′ (reverse)). Specific primers for the *Nos3* promoter region (5′-GGTTTTAGAGCCTCCCTGCC′ (forward), 5′- CTCTCAGATGCTGGCCTTCG-3′ (reverse)).

Sources of antibodies used in the experiments listed in the supplementary document

ANTIBODIES	SOURCE	CATALOG NUMBER
Glucocorticoid Receptor (GR) Rabbit pAb	Abclonal	Cat# A2164
Normal rabbit IgG	Santa Cruz	Cat# sc2027
Rabbit polyclonal to Sp7 / Osterix	Abcam	Cat#Ab22552
GLUT1/SLC2A1 Rabbit pAb	Abclonal	Cat# A6982
CPT1A Rabbit pAb	Abclonal	Cat# A5307
IL1β Rabbit mAb	Abclonal	Cat# A19635
Rabbit monoclonal [EPR23819-103] to IL-6	Abcam	Cat# ab290735
IL10 Rabbit mAb	Abclonal	Cat# A12255
Rabbit monoclonal [EPR20972] to TNF alpha	Abcam	Cat# ab215188
NOS2 Antibody (C-11)	Santa Cruz	Cat# sc-7271
CD31/PECAM-1 Antibody (Polyclonal Goat IgG)	R&D system	Cat# AF3628
Arginase 1 (ARG1) Rabbit mAb	Abclonal	Cat# A4923
eNOS Rabbit pAb	Abclonal	Cat# A1548
VE Cadherin Rabbit pAb	Abclonal	Cat# A12416
GAPDH Monoclonal Antibody	Invitrogen	Cat# MA1-16757
ZO-1 Rabbit pAb	Abclonal	Cat# A11417
Alexa Fluor® 488 anti-mouse CD36 Antibody	Biolegend	Cat#102607
Goat Anti-Mouse IgG H&L (Alexa Fluor® 594)	Abcam	Cat#ab150116
Goat Anti-Rabbit IgG H&L (Alexa Fluor® 488)	Abcam	Cat# ab150077
Donkey Anti-Goat IgG H&L (Alexa Fluor® 594)	Abcam	Cat# ab150132
Goat Anti-Rabbit IgG H&L (Alexa Fluor® 594)	Abcam	Cat# ab150080

References

1. Thiele S, Baschant U, Rauch A, and Rauner M. Instructions for producing a mouse model of glucocorticoid-induced osteoporosis. *Bonekey Rep.* 2014;3:552-.
2. Hu B, Lv X, Chen H, Xue P, Gao B, Wang X, et al. Sensory nerves regulate mesenchymal stromal cell lineage commitment by tuning sympathetic tones. *The Journal of clinical investigation.* 2020;130(7):3483-98.
3. Van der Mierden S, Leenaars CHC, Boyle EC, Ripoli FL, Gass P, Durst M, et al. Measuring endogenous corticosterone in laboratory mice - a mapping review, meta-analysis, and open source database. *Altex.* 2021;38(1):111-22.
4. Gong S, Miao YL, Jiao GZ, Sun MJ, Li H, Lin J, et al. Dynamics and correlation of serum cortisol and corticosterone under different physiological or stressful conditions in mice. *PloS one.* 2015;10(2):e0117503.
5. van Gastel N, Stegen S, Eelen G, Schoors S, Carlier A, Daniëls VW, et al. Lipid availability determines fate of skeletal progenitor cells via SOX9. *Nature.* 2020;579(7797):111-7.
6. Duvall CL, Taylor WR, Weiss D, and Guldberg RE. Quantitative microcomputed tomography analysis of collateral vessel development after ischemic injury. *American journal of physiology Heart and circulatory physiology.* 2004;287(1):H302-10.
7. Li X, Dai B, Guo J, Zhu Y, Xu J, Xu S, et al. Biosynthesized Bandages Carrying Magnesium Oxide Nanoparticles Induce Cortical Bone Formation by Modulating Endogenous Periosteal Cells. *ACS nano.* 2022.
8. Siclari VA, Zhu J, Akiyama K, Liu F, Zhang X, Chandra A, et al. Mesenchymal progenitors residing close to the bone surface are functionally distinct from those in the central bone marrow. *Bone.* 2013;53(2):575-86.
9. Shi Y, Zhang L, Pu H, Mao L, Hu X, Jiang X, et al. Rapid endothelial cytoskeletal reorganization enables

early blood-brain barrier disruption and long-term ischaemic reperfusion brain injury. *Nature communications*. 2016;7:10523.

10. Huang L, Wang X, Cao H, Li L, Chow DH, Tian L, et al. A bone-targeting delivery system carrying osteogenic phytomolecule icaritin prevents osteoporosis in mice. *Biomaterials*. 2018;182:58-71.

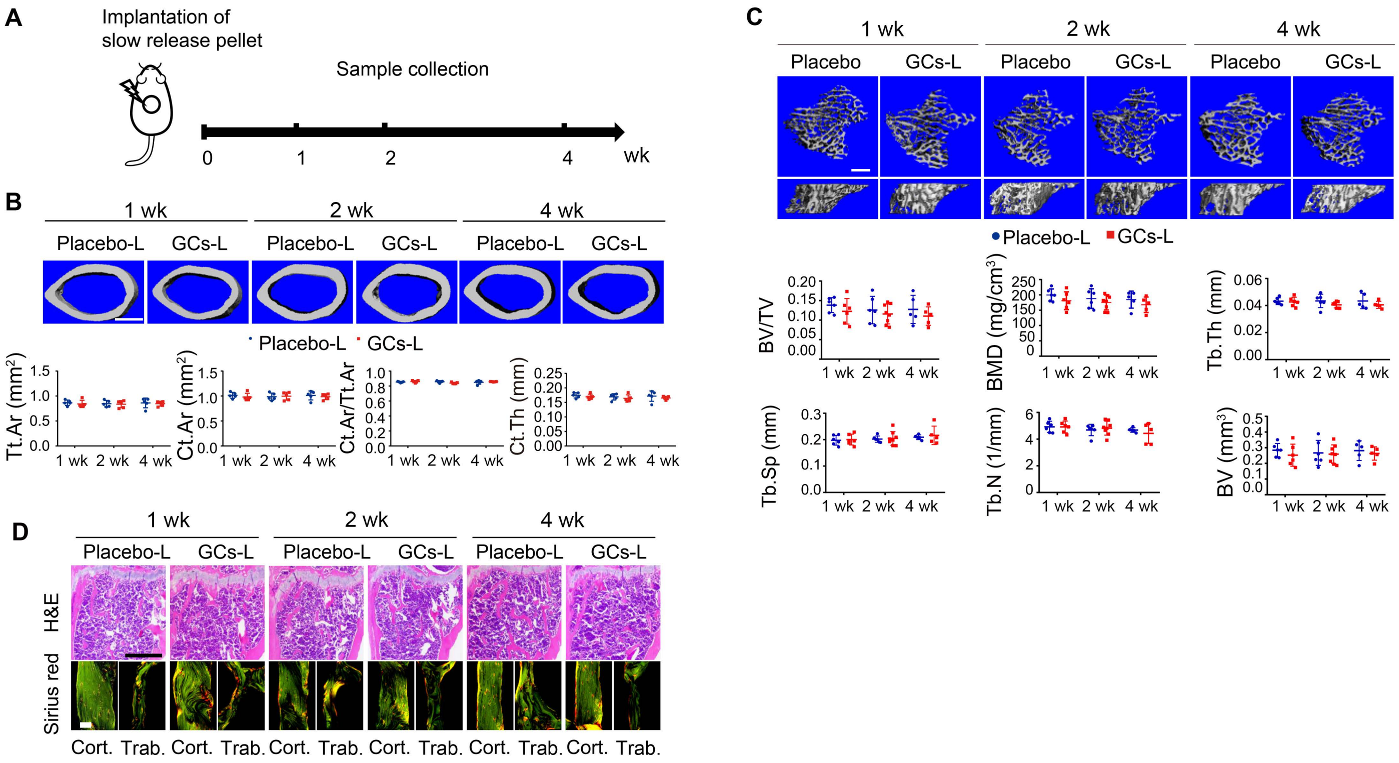
A
Implantation of slow release pellet
Sample collection
0
1
2
4
wk
B
1 wk
2 wk
4 wk
Placebo-L
GCs-L
Placebo-L
GCs-L
Placebo-L
GCs-L
Tt.Ar (mm²)
Ct.Ar (mm²)
Ct.Ar/Tt.Ar
Ct.Th (mm)
C
1 wk
2 wk
4 wk
Placebo
GCs-L
BV/TV
BMD (mg/cm³)
Tb.Th (mm)
Tb.Sp (mm)
Tb.N (1/mm)
BV (mm³)
D
H&E
Sirius red
Cort.
Trab.

Supplemental Figure 1. Bone phenotypes of placebo (Placebo-L) and low-dose (2.1 mg/kg/d) prednisolone (GCs-L)-treated male mice. (A) A schematic diagram illustrating the experimental approach for glucocorticoid-containing slow-release pellet implantation. (B) Representative micro-CT images and the quantification of total cross-sectional area (Tt.Ar), cortical bone area (Ct.Ar), cortical area fraction (Ct.Ar/Tt.Ar), and average cortical thickness (Ct.Th) in cortical bone (n=5/time point; scale bar, 500 μm). (C) Representative micro-CT images and the quantification of bone volume fraction (BV/TV), bone mineral density (BMD), trabecular thickness (Tb.Th), trabecular separation (Tb.Sp), trabecular number (Tb.N) and bone volume (BV) in trabeculae (n=5~8/time point; scale bar, 500 μm). (D) Representative hematoxylin and eosin (H&E) (scale bar, 500 μm) and Sirius red (scale bar, 50 μm) staining of proximal tibia. Data are mean ± SD. No statistical difference as analyzed by two-way ANOVA with Bonferroni *post hoc* test.

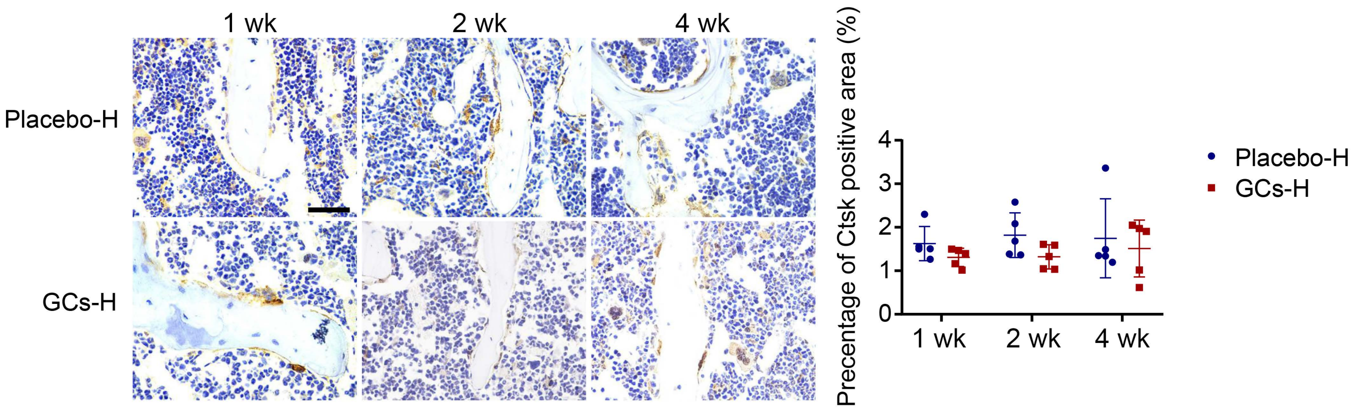
1 wk
2 wk
4 wk
Placebo-H
GCs-H
Precentage of Ctsk positive area (%)
4
3
2
1
0
1 wk
2 wk
4 wk
Placebo-H
GCs-H

Supplemental Figure 2. Representative immunohistochemical image of Ctsk and the quantification of Ctsk positive area in the placebo (Placebo-H) and high-dose (6.25 mg/kg/d) prednisolone (GCs-H)-treated male mice (n=5/time point; scale bar, 50 μm). Data are mean ± SD. No statistical difference as analyzed by two-way ANOVA with Bonferroni *post hoc* test.

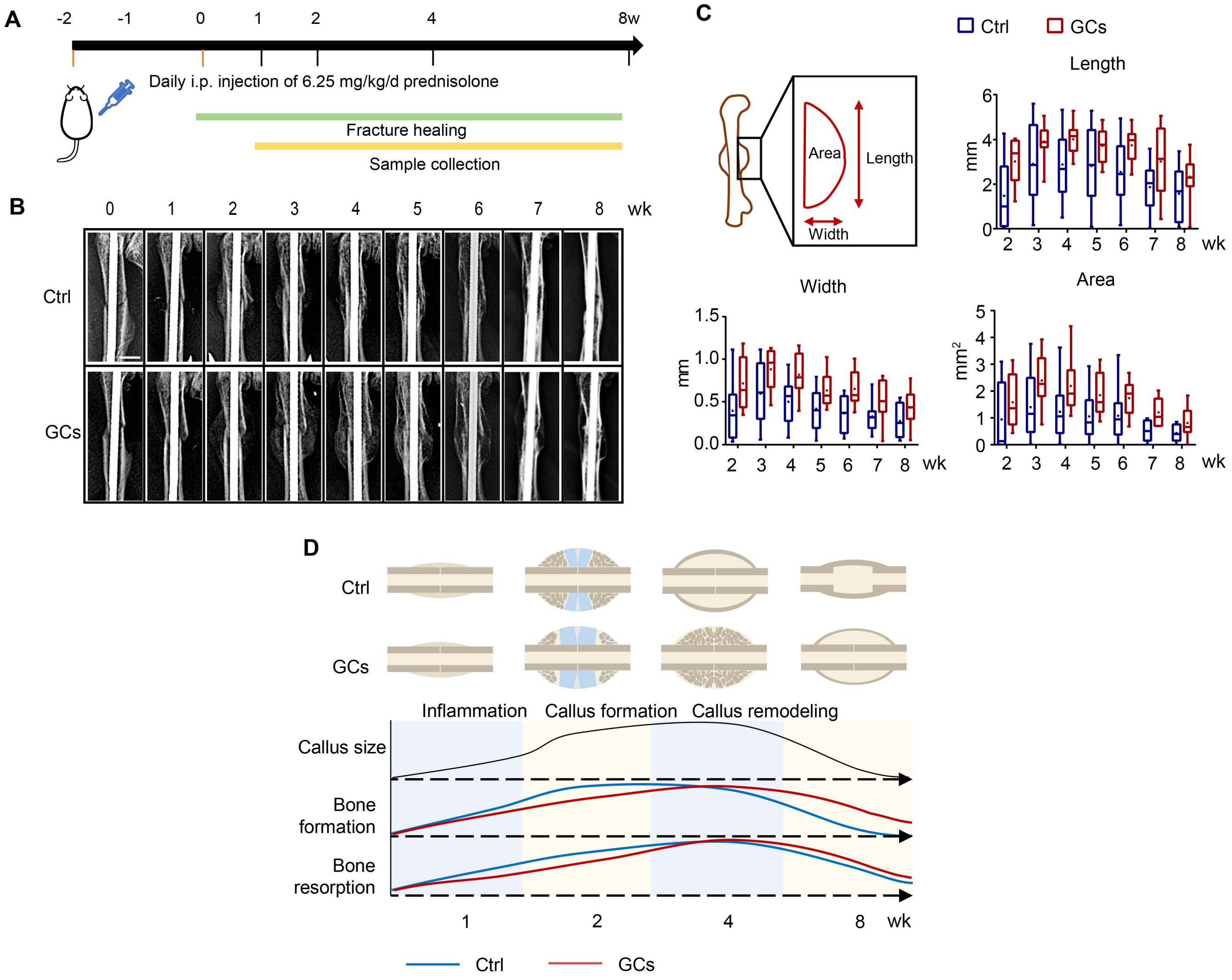
A
-2
-1
0
1
2
4
8w
Daily i.p. injection of 6.25 mg/kg/d prednisolone
Fracture healing
Sample collection
B
0
1
2
3
4
5
6
7
8
wk
Ctrl
GCs
C
Ctrl
GCs
Area
Length
Width
Length
mm
Width
mm
Area
mm^2
wk
D
Ctrl
GCs
Inflammation
Callus formation
Callus remodeling
Callus size
Bone formation
Bone resorption
1
2
4
8
wk
Ctrl
GCs

Supplemental Figure 3. Excess glucocorticoid exposure delays fracture healing in male mice. (A) A schematic diagram illustrating the experimental approach for glucocorticoid delivery and fracture model establishment. 20-week-old C57Bl/6J male mice were injected daily with prednisolone (6.25 mg/kg/d) for two weeks before fracture surgery. The daily injection of glucocorticoids was performed during the *de novo* formation of skeletal tissue. (B-C) Representative radiographs (B) (scale bar, 500 μm) and quantification of the length, width and area (C, n=8~12) of the callus during fracture healing. (D) A schematic diagram illustrating fracture healing under normal conditions and during exposure to glucocorticoids. Glucocorticoid exposure delays fracture healing by reducing bone formation and resorption at the initial phase (week 2 to 4). Data are mean ±SD. No statistical difference as analyzed by two-way ANOVA with Bonferroni *post hoc* test.

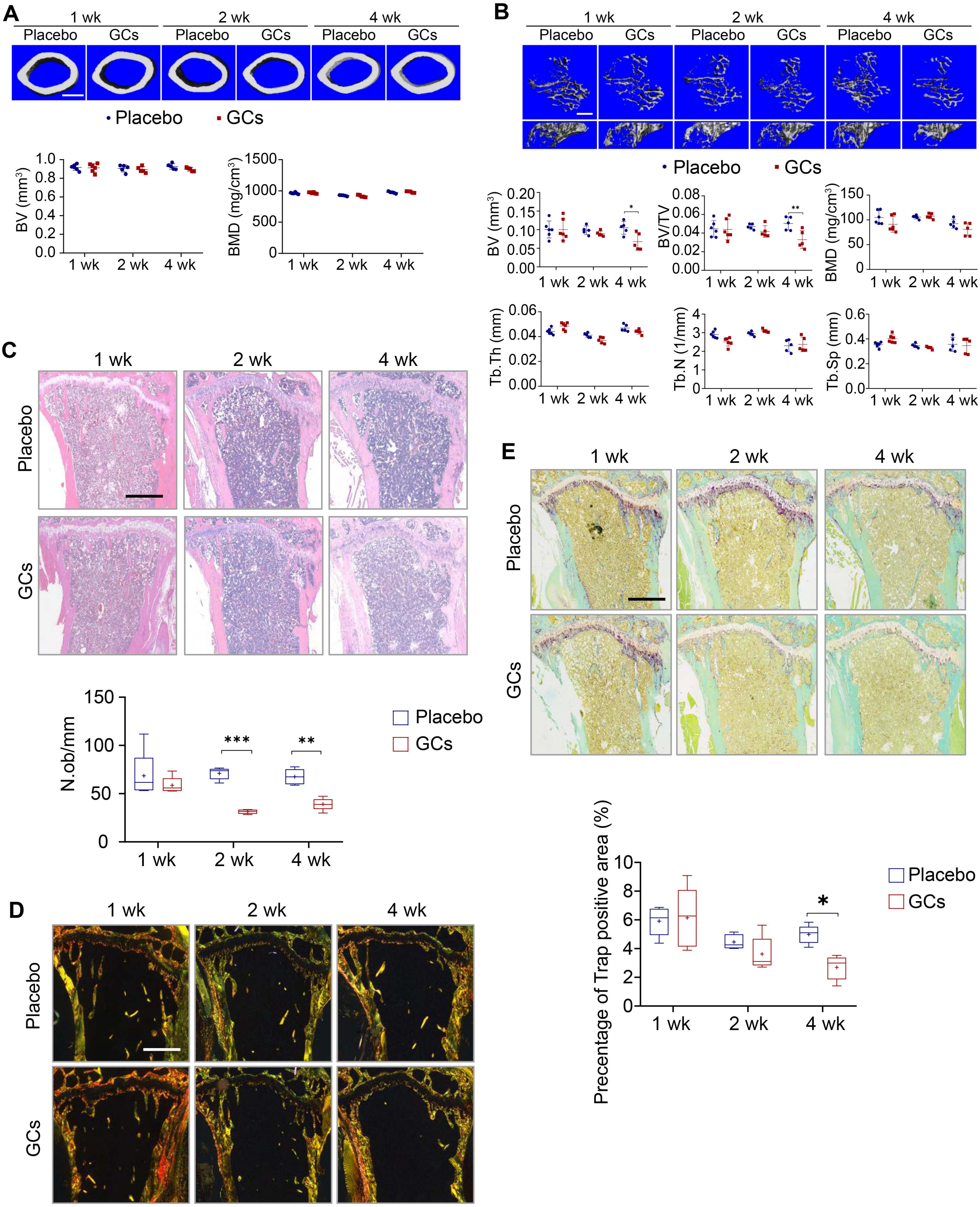

A
1 wk
2 wk
4 wk
Placebo
GCs
Placebo
GCs
BV (mm³)
BMD (mg/cm³)
B
Placebo
GCs
BV (mm³)
BV/TV
BMD (mg/cm³)
Tb.Th (mm)
Tb.N (1/mm)
Tb.Sp (mm)
*
**
C
N.ob/mm
***
**
D
E
Precentage of Trap positive area (%)
*

Supplemental Figure 4. Glucocorticoid-induced bone loss coincides with reduced bone turnover in female mice. (A) Representative micro-CT images and the quantification of BV, and BMD in cortical bone (n= 5~6/time point; scale bar, 500μm). (B) Representative micro-CT images and quantification of bone volume fraction (BV/TV), bone mineral density (BMD), trabecular thickness (Tb.Th), trabecular separation (Tb.Sp), trabecular number (Tb.N) and bone volume (BV) in trabecula (n= 5~6/time point; Scale bar, 500 μm). (C) Representative H&E staining of tibia and the quantification of number of osteoblast per mm (N.ob/mm) (n= 5/time point; scale bar, 500 μm). (D) Sirius red (scale bar, 50 μm) staining of tibia. (E) Representative Trap staining of tibia and the quantification of percentage of Trap positive area (n= 5/time point; scale bar, 500 μm). Data are mean ±SD. *$P < 0.05$, **$P < 0.01$, ***$P < 0.001$ by two-way ANOVA (A, B, C and E) with Bonferroni *post hoc* test.

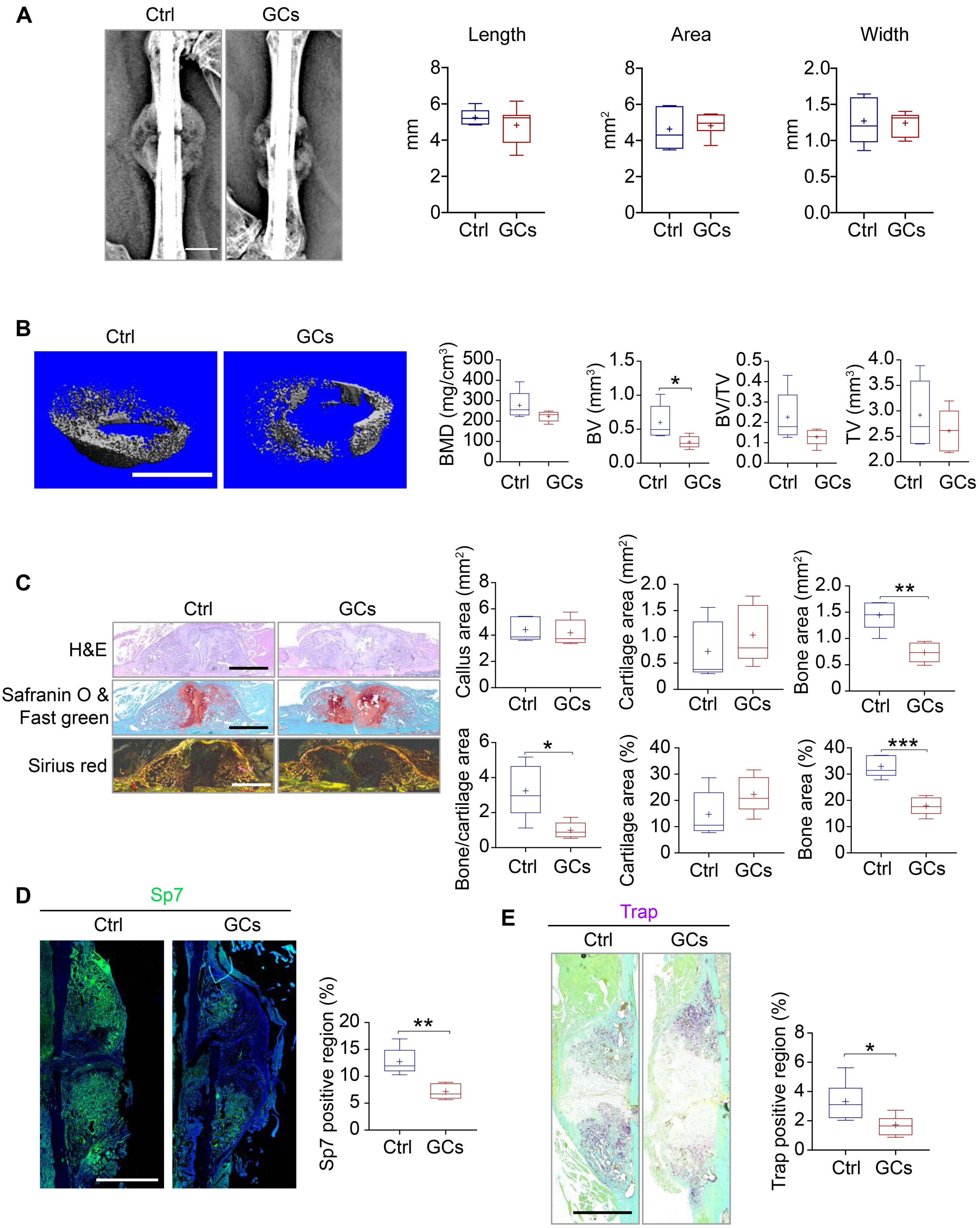

A
Ctrl
GCs
Length
Area
Width
mm
mm2
8
6
4
2
0
2.0
1.5
1.0
0.5
0.0
Ctrl GCs
B
Ctrl
GCs
BMD (mg/cm3)
BV (mm3)
BV/TV
TV (mm3)
500
400
300
200
100
0
1.5
1.0
0.5
0.0
0.5
0.4
0.3
0.2
0.1
0.0
4.0
3.5
3.0
2.5
2.0
*
C
Ctrl
GCs
H&E
Safranin O & Fast green
Sirius red
Callus area (mm2)
Cartilage area (mm2)
Bone area (mm2)
Bone/cartilage area
Cartilage area (%)
Bone area (%)
**
*
***
D
Sp7
Ctrl
GCs
Sp7 positive region (%)
20
15
10
5
0
**
E
Trap
Ctrl
GCs
Trap positive region (%)
8
6
4
2
0
*

Supplemental Figure 5. Glucocorticoids inhibit callus formation and delay fracture healing in female mice. (A) Representative radiographs (scale bar, 500 μm) and quantification of the length, width and area of the callus at week 2 (n=5~7). (B) Representative micro-CT images and quantification of bone volume fraction (BV/TV), bone mineral density (BMD), total volume (TV) and bone volume (BV) (n=5). (C) Representative hematoxylin & eosin (H&E), Safranin O & Fast green, and Sirius red staining, and quantification of week 2 calluses (n=5; scale bar, 1 mm). (D) Representative immunofluorescence images of Sp7 (green) and quantification of Sp7-positive area (Scale bar, 1mm; n=5). (E) Representative Trap staining images and quantification of Trap-positive area (Scale bar, 1mm; n=6~7). Data are mean ±SD. *$P < 0.05$, **$P < 0.01$, ***$P < 0.001$ by two-tailed Student's *t*-test (A, B, C, D and E).

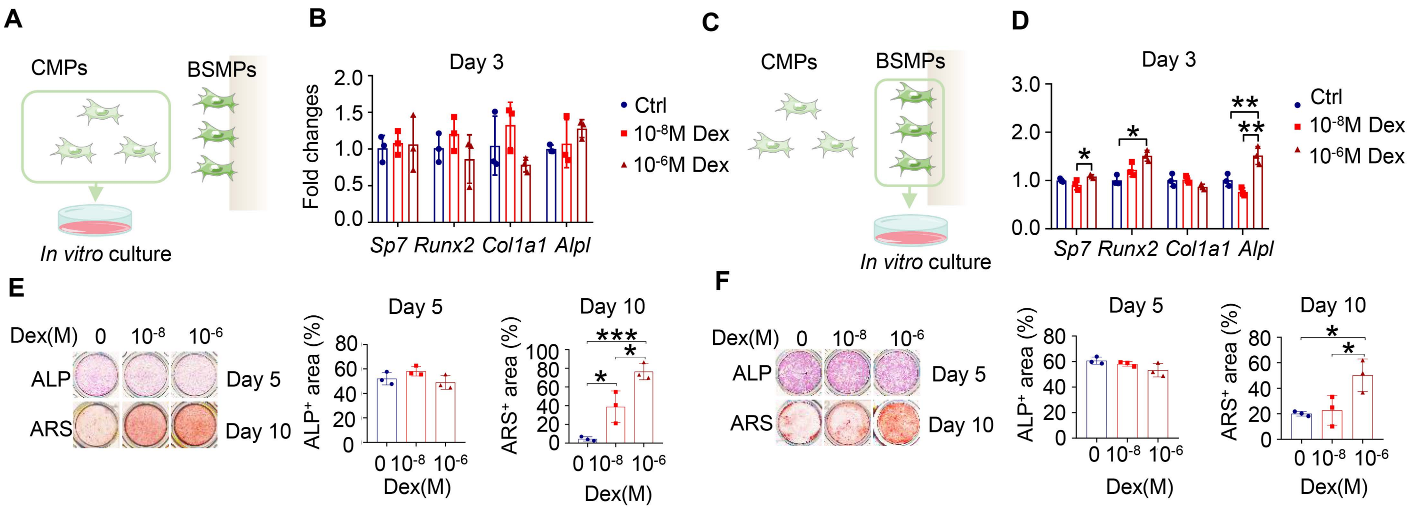
A
CMPs
BSMPs
In vitro culture
B
Day 3
Fold changes
Sp7 Runx2 Col1a1 Alpl
Ctrl
10-8M Dex
10-6M Dex
C
CMPs
BSMPs
In vitro culture
D
Day 3
Sp7 Runx2 Col1a1 Alpl
Ctrl
10-8M Dex
10-6M Dex
E
Dex(M) 0 10-8 10-6
ALP
ARS
Day 5
Day 10
ALP+ area (%)
ARS+ area (%)
Dex(M)
F
Dex(M) 0 10-8 10-6
ALP
ARS
Day 5
Day 10
ALP+ area (%)
ARS+ area (%)
Dex(M)

Supplemental Figure 6. Effects of glucocorticoids on osteogenesis *in vitro*. (A-D) Gene expression (at day 3) of CMPs (A and B) or BSMPs (C and D) during osteogenic induction by Dex (n=3). (E and F) Alkaline phosphatase (ALP) (at day 5) and alizarin red S (ARS) (at day 10) staining analysis of BSMPs (E) or CMPs (F) supplemented with dexamethasone (Dex) (n=3). (D-F) Gene expression (at day 3, D), ALP (at day 5, E) and ARS (at day 10, F) staining analysis of CMPs during osteogenic induction by Dex (n=3). Data are mean ±SD. *$P < 0.05$, **$P < 0.01$, ***$P < 0.001$ by one-way ANOVA (B, D, E, and F) with Bonferroni *post hoc* test.

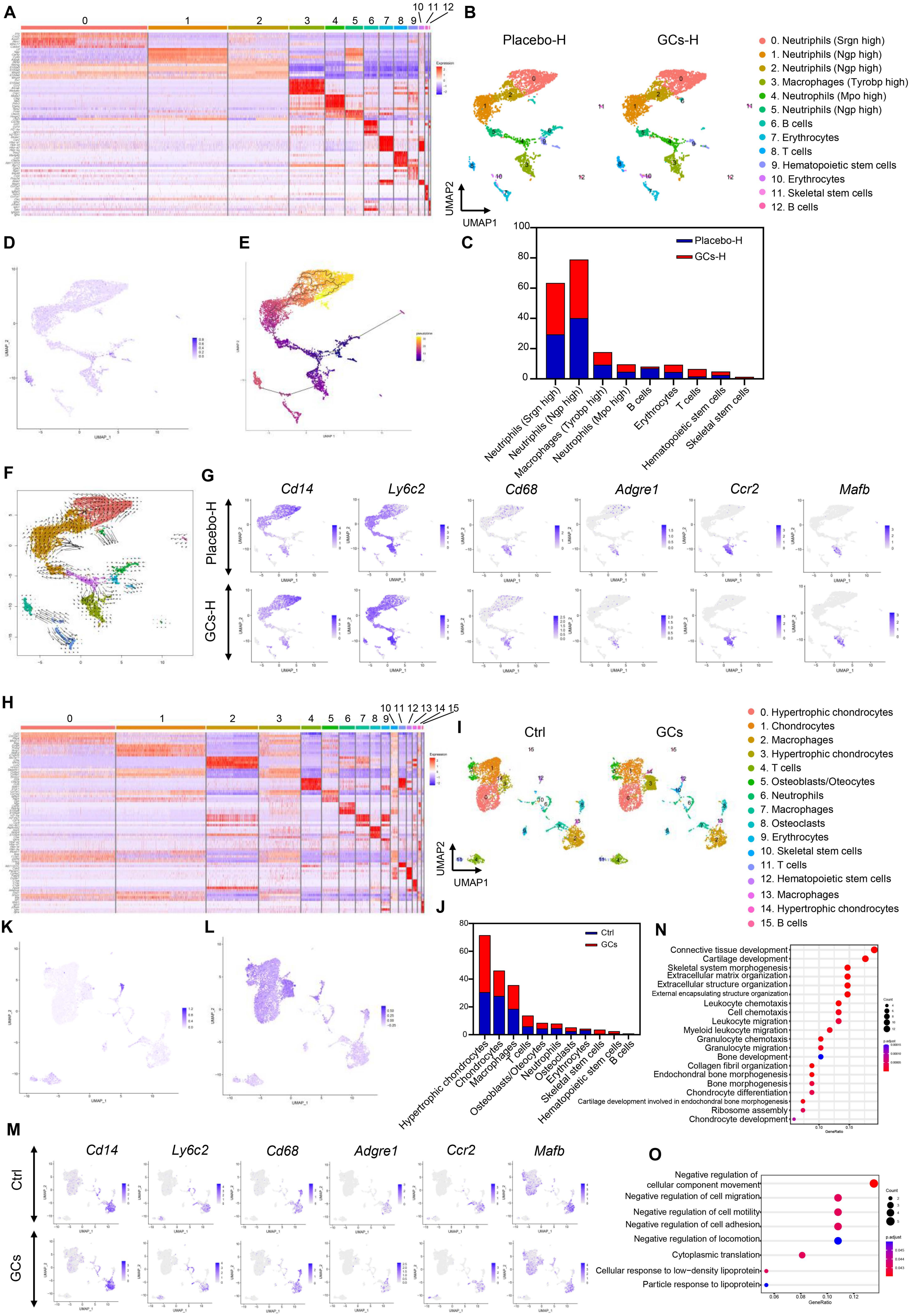

A
B
Placebo-H
GCs-H
0. Neutriphils (Srgn high)
1. Neutriphils (Ngp high)
2. Neutriphils (Ngp high)
3. Macrophages (Tyrobp high)
4. Neutriphils (Mpo high)
5. Neutriphils (Ngp high)
6. B cells
7. Erythrocytes
8. T cells
9. Hematopoietic stem cells
10. Erythrocytes
11. Skeletal stem cells
12. B cells
UMAP2
UMAP1
C
Placebo-H
GCs-H
Neutriphils (Srgn high)
Neutriphils (Ngp high)
Macrophages (Tyrobp high)
Neutriphils (Mpo high)
B cells
Erythrocytes
T cells
Hematopoietic stem cells
Skeletal stem cells
D
E
F
G
Cd14
Ly6c2
Cd68
Adgre1
Ccr2
Mafb
Placebo-H
GCs-H
H
I
Ctrl
GCs
0. Hypertrophic chondrocytes
1. Chondrocytes
2. Macrophages
3. Hypertrophic chondrocytes
4. T cells
5. Osteoblasts/Oteocytes
6. Neutrophils
7. Macrophages
8. Osteoclasts
9. Erythrocytes
10. Skeletal stem cells
11. T cells
12. Hematopoietic stem cells
13. Macrophages
14. Hypertrophic chondrocytes
15. B cells
J
Ctrl
GCs
Hypertrophic chondrocytes
Chondrocytes
Macrophages
T cells
Osteoblasts/Oteocytes
Neutrophils
Osteoclasts
Erythrocytes
Skeletal stem cells
Hematopoietic stem cells
B cells
K
L
N
Connective tissue development
Cartilage development
Skeletal system morphogenesis
Extracellular matrix organization
Extracellular structure organization
External encapsulating structure organization
Leukocyte chemotaxis
Cell chemotaxis
Leukocyte migration
Myeloid leukocyte migration
Granulocyte chemotaxis
Granulocyte migration
Bone development
Collagen fibril organization
Endochondral bone morphogenesis
Bone morphogenesis
Chondrocyte differentiation
Cartilage development involved in endochondral bone morphogenesis
Ribosome assembly
Chondrocyte development
GeneRatio
M
Cd14
Ly6c2
Cd68
Adgre1
Ccr2
Mafb
Ctrl
GCs
O
Negative regulation of cellular component movement
Negative regulation of cell migration
Negative regulation of cell motility
Negative regulation of cell adhesion
Negative regulation of locomotion
Cytoplasmic translation
Cellular response to low−density lipoprotein
Particle response to lipoprotein
Count
p.adjust
GeneRatio

Supplemental Figure 7. Identification of cell populations in glucocorticoids-induced bone loss and in associated fracture healing models using scRNA-seq. (A) Heatmap of top differentially expressed genes (DEGs) within cell populations of the trabecular bone surface in a glucocorticoids-induced bone loss model in mice. (B) UMAP representation of bone surface cells measured with scRNA-seq. Each point represents an individual cell. (C) Relative proportion of each bone surface cell cluster. (D) Stemness analysis of hematopoietic cell lineages. (E) Pseudotime ordering of bone surface cells. (F) RNA velocity field onto the t-SNE plot of bone surface cells. (G) Feature plot of monocytes or macrophages markers expressed in bone surface cells. (H) Heatmap of top differentially expressed genes within cell populations of week 2 fracture callus. (I) UMAP representation of callus cells measured with scRNA-seq. Each point represents an individual cell. (J) Relative proportion of each callus cell cluster. (K-I) Stemness analysis of hematopoietic lineages (K) and osteolineages (I). (M) Feature plot of monocytes or macrophages markers expressed in callus cells. (N and O) Enriched GO functions of downregulated (N) and upregulated (O) genes in macrophages in the fracture model.

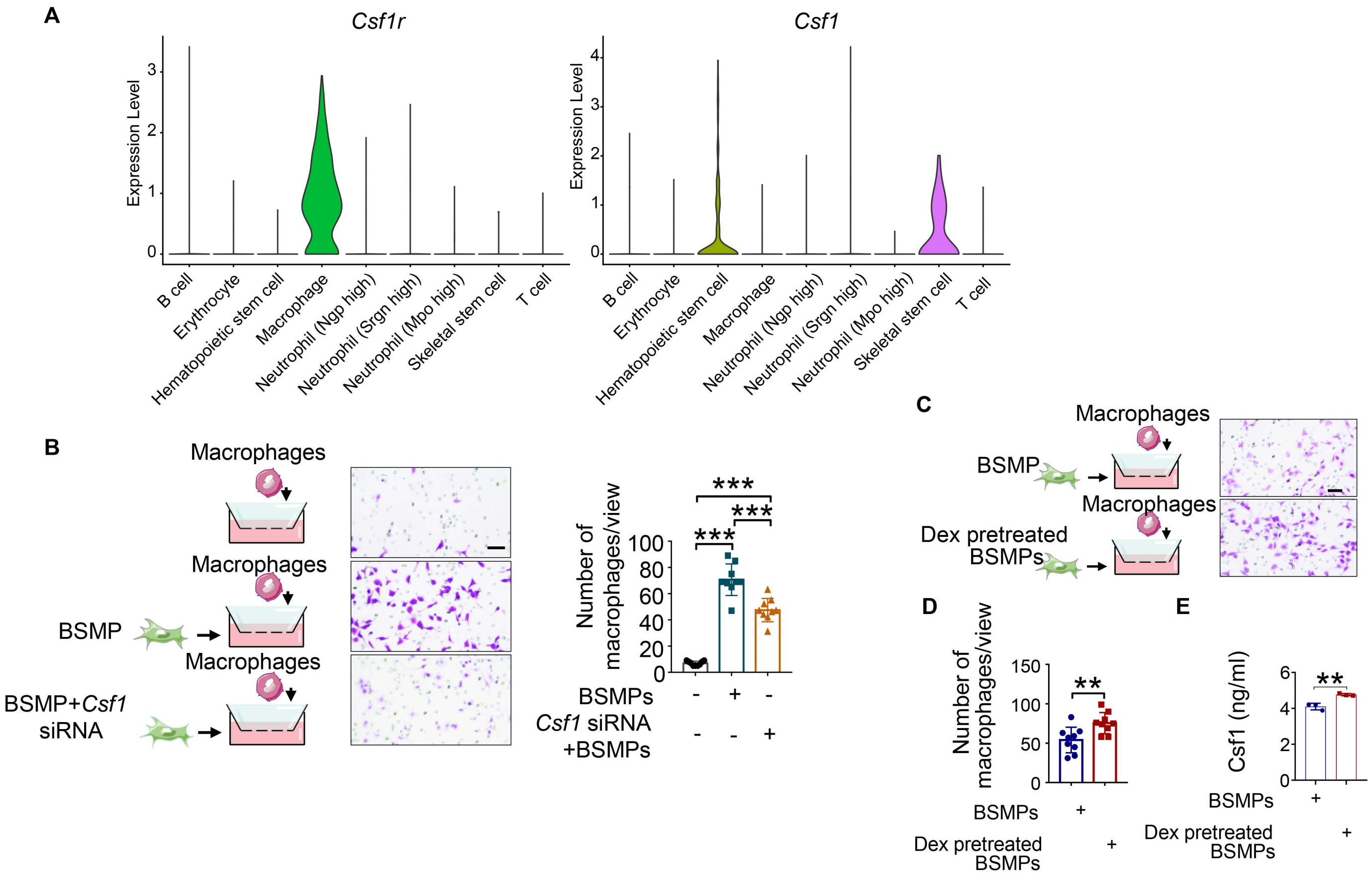
A
Csf1r
Csf1
Expression Level
B cell
Erythrocyte
Hematopoietic stem cell
Macrophage
Neutrophil (Ngp high)
Neutrophil (Srgn high)
Neutrophil (Mpo high)
Skeletal stem cell
T cell
B
Macrophages
BSMP
BSMP+Csf1 siRNA
Number of macrophages/view
***
BSMPs
Csf1 siRNA +BSMPs
C
Dex pretreated BSMPs
D
E
**
Csf1 (ng/ml)
Dex pretreated BSMPs

Supplemental Figure 8. Interactions of bone forming cells and macrophages through Csf1 signaling. (A) Violin plots of normalized expression of Csf1 and Csf1r in bone surface cells. (B) Transwell assays of macrophage migration recruited by BSMPs and *Csf1* siRNA transfected BSMPs (n=9; scale bar, 50μm). (C and D) Transwell assays of macrophage migration recruited by BSMPs or Dex pretreated BSMPs (n=9; scale bar, 50μm). (E) Csf1 production from BSMPs or Dex pretreated BSMPs (n=3). Data are mean ±SD. *$P < 0.05$, **$P < 0.01$, ***$P < 0.001$ by one-way ANOVA (B) with Bonferroni *post hoc* test or two-tailed Student's *t*-test (D and E).

.

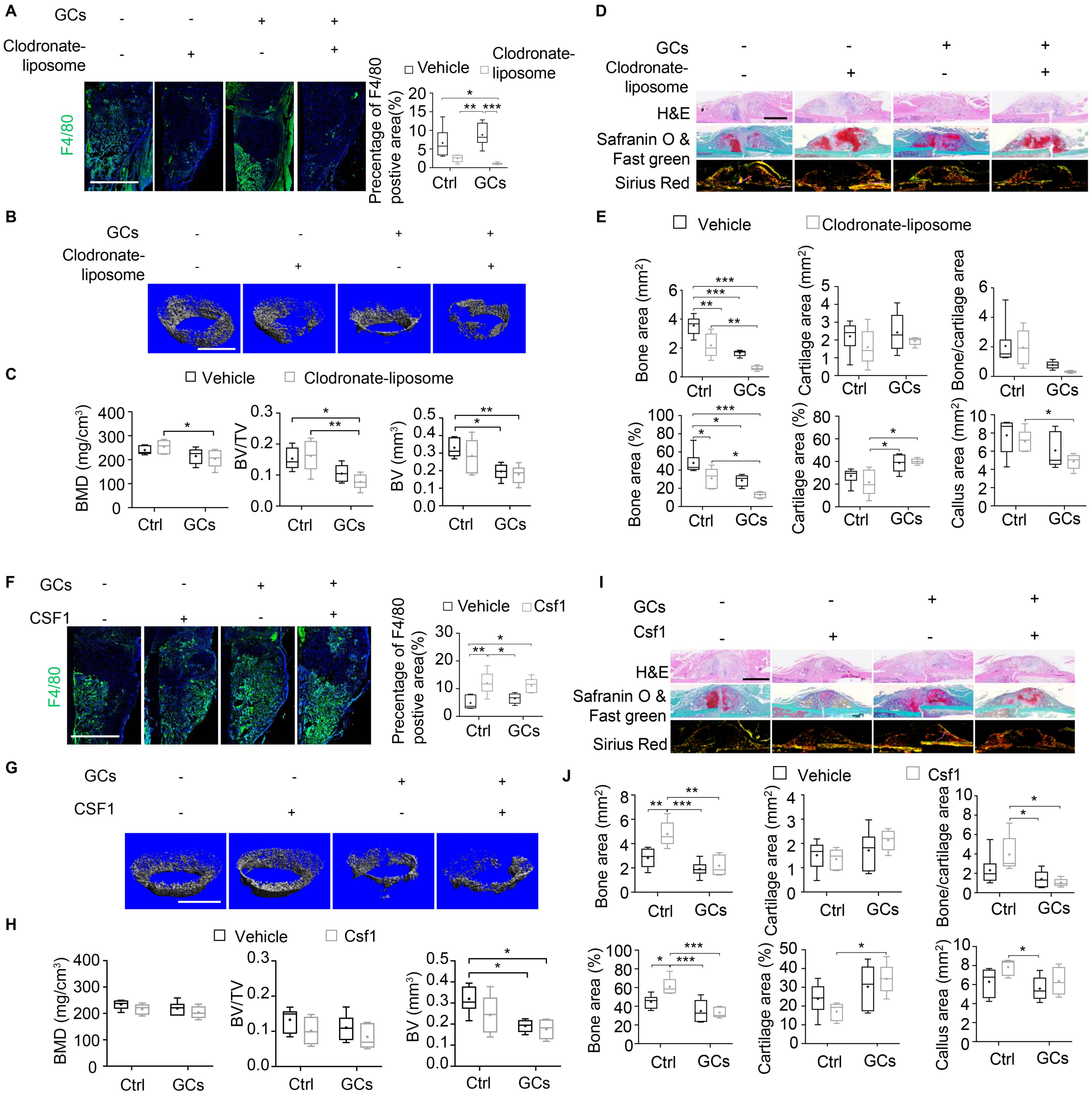

A
GCs
Clodronate-liposome
F4/80
Precentage of F4/80 postive area(%)
Vehicle
Clodronate-liposome
Ctrl
GCs
B
GCs
Clodronate-liposome
C
Vehicle
Clodronate-liposome
BMD (mg/cm3)
BV/TV
BV (mm3)
Ctrl
GCs
D
GCs
Clodronate-liposome
H&E
Safranin O & Fast green
Sirius Red
E
Vehicle
Clodronate-liposome
Bone area (mm2)
Cartilage area (mm2)
Bone/cartilage area
Bone area (%)
Cartilage area (%)
Callus area (mm2)
Ctrl
GCs
F
GCs
CSF1
F4/80
Precentage of F4/80 postive area(%)
Vehicle
Csf1
Ctrl
GCs
G
GCs
CSF1
H
Vehicle
Csf1
BMD (mg/cm3)
BV/TV
BV (mm3)
Ctrl
GCs
I
GCs
Csf1
H&E
Safranin O & Fast green
Sirius Red
J
Vehicle
Csf1
Bone area (mm2)
Cartilage area (mm2)
Bone/cartilage area
Bone area (%)
Cartilage area (%)
Callus area (mm2)
Ctrl
GCs

Supplemental Figure 9. Macrophages modulate bone formation *in vivo*. (A) Representative immunofluorescence images of F4/80 (green) and quantification of F4/80 positive area of week 2 calluses from vehicle control (Ctrl) and 6.25 mg/kg/d of prednisolone (GCs)-treated mice with or without local depletion of macrophages by clodronate liposome (n=5~6; scale bar, 1mm). Representative micro-CT images (B) and quantification (C) of (distal ends) BMD, BV/TV and BV in the callus from vehicle control (ctrl) and GCs-treated mice with or without local depletion of macrophages (n= 5~6/time point; scale bar, 500 μm). Representative H&E, Safranin O & Fast green, and Sirius red staining (D) and quantification (E) of week 2 calluses from Ctrl and GCs-treated mice with or without local depletion of macrophages (n=5~7; scale bar, 1mm). (F) Representative immunofluorescence images of F4/80 (green) and quantification of F4/80 positive area of week 2 calluses from Ctrl and GCs-treated mice with or without local injection of Csf1 (n=5~6; scale bar, 1mm). Representative micro-CT images (G) and quantification (H) of (distal ends) BMD, BV/TV and BV in the callus from Ctrl and GCs-treated mice with or without local injection of Csf1 (n= 5~7/time point; scale bar, 500 μm). Representative H&E, Safranin O & Fast green, and Sirius red staining (I) and quantification (J) of week 2 calluses from Ctrl and GCs-treated mice with or without local injection of Csf1 (n=5~6; scale bar, 1mm). Data are mean ±SD. *$P < 0.05$, **$P < 0.01$, ***$P < 0.001$ by two-way ANOVA (A, C, E, F, H and J) with Bonferroni *post hoc* test.

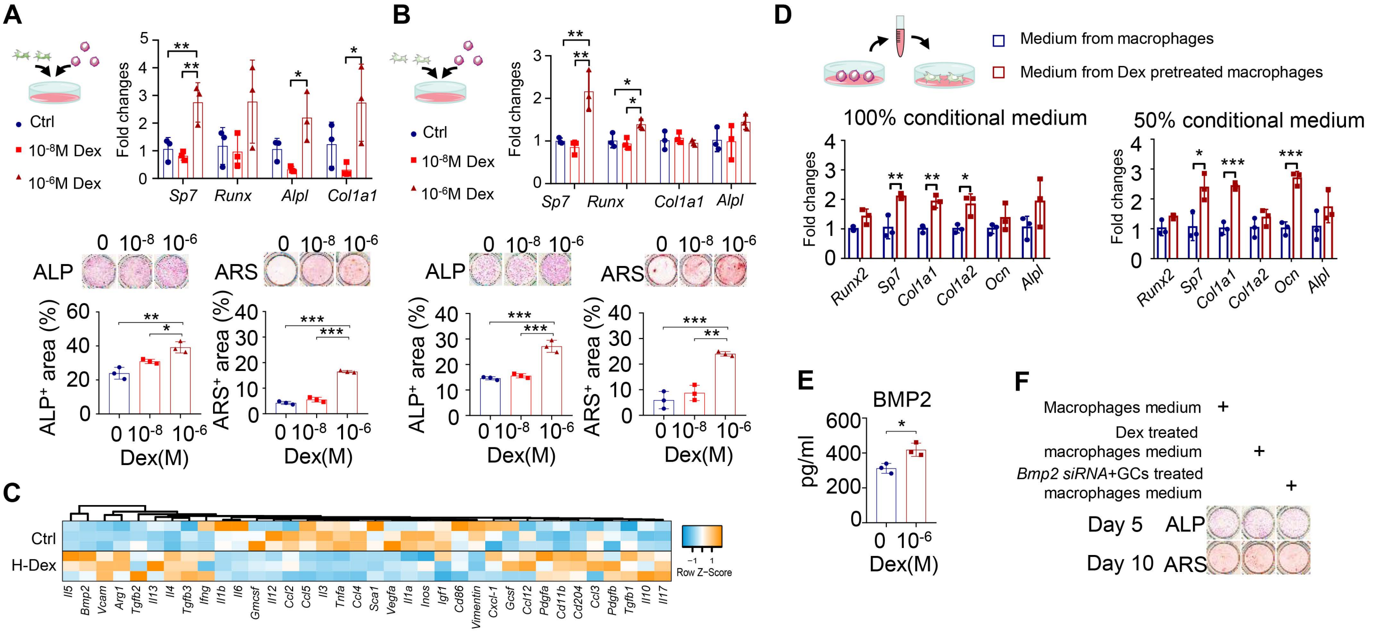

A
Ctrl
10-8M Dex
10-6M Dex
Fold changes
Sp7
Runx
Alpl
Col1a1
ALP
ARS
0 10-8 10-6
ALP+ area (%)
ARS+ area (%)
Dex(M)
B
Ctrl
10-8M Dex
10-6M Dex
Fold changes
Sp7
Runx
Col1a1
Alpl
ALP
ARS
ALP+ area (%)
ARS+ area (%)
Dex(M)
C
Ctrl
H-Dex
Row Z-Score
Il5
Bmp2
Vcam
Arg1
Tgfb2
Il13
Il4
Tgfb3
Ifng
Il1b
Il6
Gmcsf
Il12
Ccl2
Ccl5
Il3
Tnfa
Ccl4
Sca1
Vegfa
Il1a
Inos
Igf1
Cd86
Vimentin
Cxcl-1
Gcsf
Ccl12
Pdgfa
Cd11b
Cd204
Ccl3
Pdgfb
Tgfb1
Il10
Il17
D
Medium from macrophages
Medium from Dex pretreated macrophages
100% conditional medium
50% conditional medium
Fold changes
Runx2
Sp7
Col1a1
Col1a2
Ocn
Alpl
E
BMP2
pg/ml
0 10-6
Dex(M)
F
Macrophages medium
Dex treated macrophages medium
Bmp2 siRNA+GCs treated macrophages medium
Day 5 ALP
Day 10 ARS

Supplemental Figure 10. Macrophages modulate osteogenesis *in vitro*. (A and B) Gene expression (at day 3), ALP (at day 5) and Alizarin Red S (ARS) (at day 10) staining of CMPs (A) or BSMPs (B) cocultured with macrophages during osteogenesis (n=3). (C) Cytokines transcription of macrophages treated with H-Dex. (D) Gene expression of BSMPs cocultured with conditional medium from Dex pretreated macrophages (n=3). (E) Supernatants concentration of BMP2 in BSMPs or Dex treated BSMPs (n=3). (F) ALP (at day 5) and ARS (at day 10) staining of BSMPs cultured with supernatants from macrophages with or without Dex or *BMP2* siRNA pretreatment. Data are mean ±SD. *$P < 0.05$, **$P < 0.01$, ***$P < 0.001$ by one-way ANOVA (A and B) with Bonferroni *post hoc* test or two-tailed Student's *t*-test (D and E).

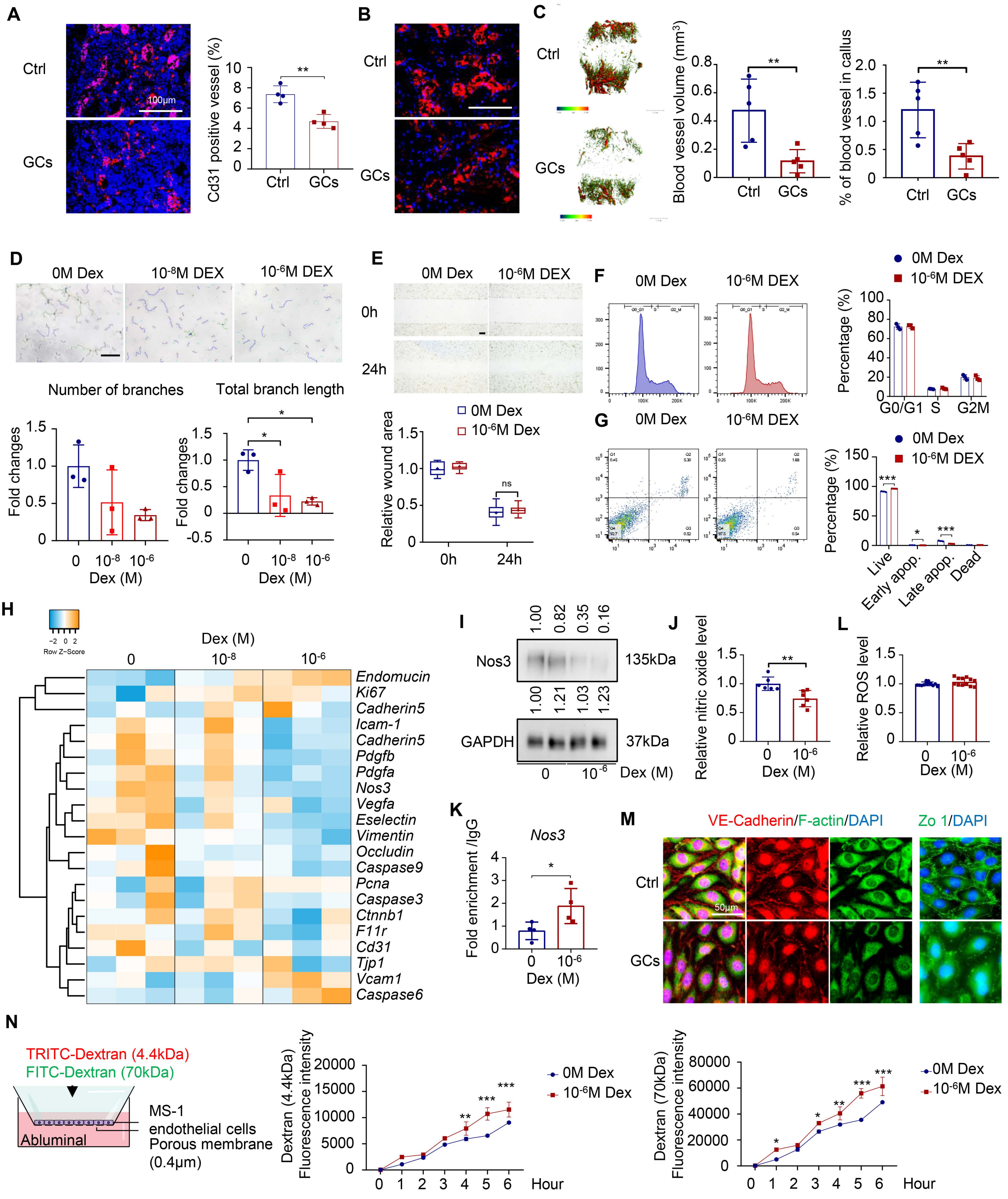

A
Ctrl
GCs
100μm
Cd31 positive vessel (%)
**
B
Ctrl
GCs
C
Ctrl
GCs
Blood vessel volume (mm³)
% of blood vessel in callus
D
0M Dex
10-8M DEX
10-6M DEX
Number of branches
Total branch length
Fold changes
Dex (M)
E
0M Dex
10-6M DEX
0h
24h
Relative wound area
ns
F
0M Dex
10-6M DEX
Percentage (%)
G0/G1
S
G2M
G
Live
Early apop.
Late apop.
Dead
H
Row Z-Score
Dex (M)
0
10-8
10-6
Endomucin
Ki67
Cadherin5
Icam-1
Cadherin5
Pdgfb
Pdgfa
Nos3
Vegfa
Eselectin
Vimentin
Occludin
Caspase9
Pcna
Caspase3
Ctnnb1
F11r
Cd31
Tjp1
Vcam1
Caspase6
I
1.00 0.82 0.35 0.16
Nos3
135kDa
1.00 1.21 1.03 1.23
GAPDH
37kDa
Dex (M)
J
Relative nitric oxide level
L
Relative ROS level
K
Nos3
Fold enrichment /IgG
M
VE-Cadherin/F-actin/DAPI
Zo 1/DAPI
Ctrl
GCs
50μm
N
TRITC-Dextran (4.4kDa)
FITC-Dextran (70kDa)
MS-1
endothelial cells
Porous membrane
(0.4μm)
Abluminal
Dextran (4.4kDa) Fluorescence intensity
Dextran (70kDa) Fluorescence intensity
0M Dex
10-6M Dex
Hour

Supplemental Figure 11. Glucocorticoids directly inhibit blood vessel formation and obstruct vascular invasion. (A) Immunofluorescence analysis and quantification of Cd31-positive blood vessels (red) in the glucocorticoid-induced bone loss model (n=4; scale bar, 100 μm). (B) Immunofluorescence analysis of Cd31-positive blood vessels (red) in the glucocorticoid-associated fracture healing model (Scale bar, 100 μm). (C) Micro-CT-based visualization and quantification of blood vessel in the glucocorticoid-associated fracture healing model (scale bar, 1 mm; n=5). (D) Tube formation assay of MS-1 cells (a pancreatic endothelial cell line) treated with Dex. Top, representative images; bottom, quantification of the number and length of branches (n=3; scale bar, 200 μm). (E) Wound- healing assay of MS-1 cells treated with Dex. Top, representative images; bottom, quantification of wound area (n=16; scale bar, 100 μm). Cell cycle (F) and apoptosis (G) measured by flow cytometry assay in MS-1 cells treated with Dex (n=3). (H) Transcriptional levels of cytokines in MS-1 cells treated with Dex. (I) Representative Western blots of iNOS and GAPDH of MS-1 cells treated Dex. (J) Relative nitric oxide level in MS-1 cells treated with 10^{-6} M Dex (n=6). (K) Occupancy of GR at the *Nos3* promoter of MS-1 cells treated with 10^{-6} M Dex (n=4). (L) Relative reactive oxygen species (ROS) level in MS-1 cells treated with 10^{-6} M Dex (n=12). (M) MS-1 cells treated with 10^{-6} M Dex stained for VE-cadherin, F-actin and Zo-1 and counterstained with DAPI for nuclear labelling (scale bar, 50 μm). (N) Paracellular permeability assay of MS-1 cells treated with 10^{-6} M Dex as measured by diffusion coefficient of the two fluorescent tracers 4.4 kDa TRITC-dextran or 70 kDa FITC-dextran (n=3). Data are mean ± SD. **P* < 0.05, ***P* < 0.01, ****P* < 0.001 by one-way ANOVA (D), two-way ANOVA (F and N) with Bonferroni *post hoc* test or two-tailed Student's *t*-test (A, C, J, K, and L).

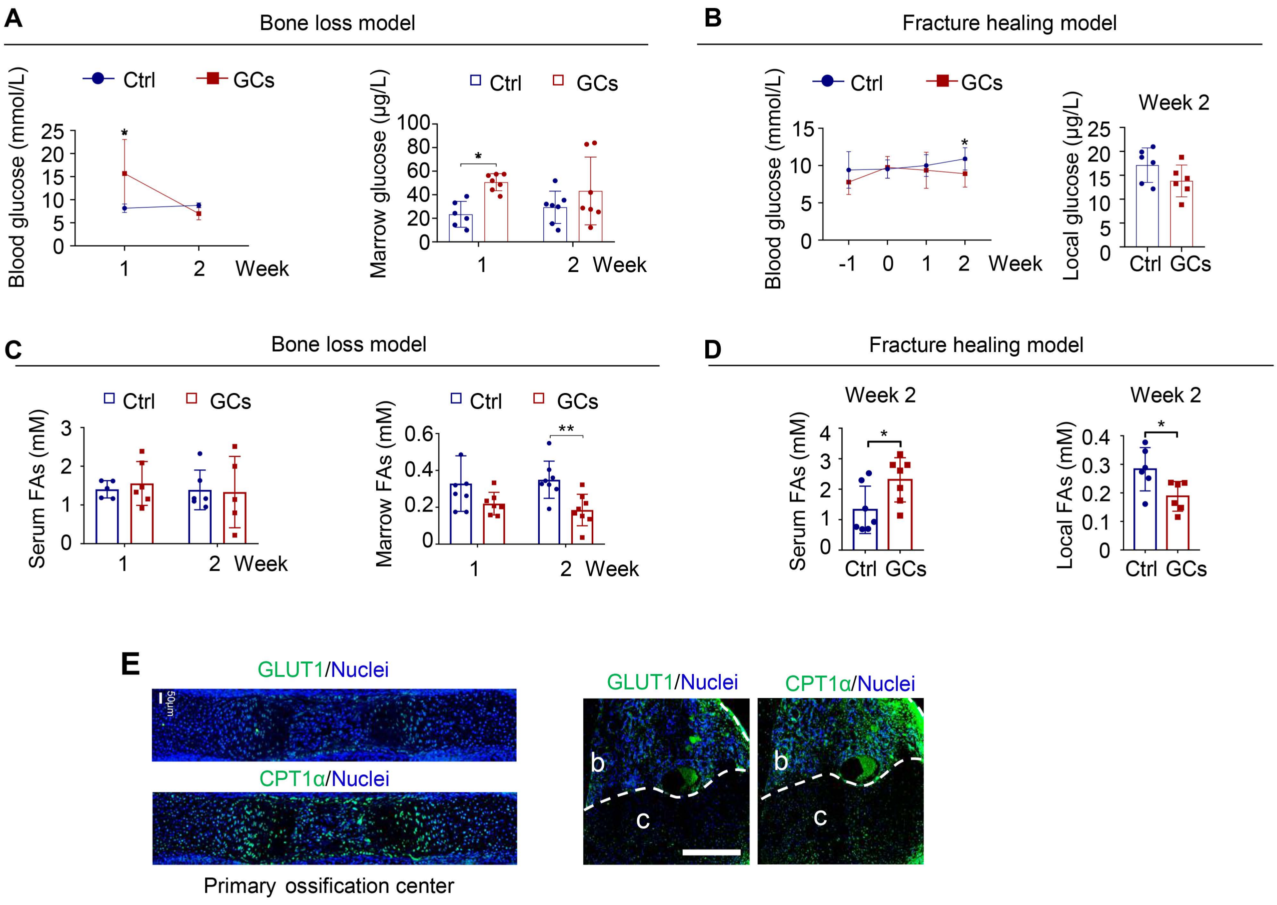
A
Bone loss model
Ctrl
GCs
Blood glucose (mmol/L)
Week
Marrow glucose (μg/L)
B
Fracture healing model
Week 2
Local glucose (μg/L)
C
Bone loss model
Serum FAs (mM)
Marrow FAs (mM)
D
Fracture healing model
Local FAs (mM)
E
GLUT1/Nuclei
CPT1α/Nuclei
50um
Primary ossification center
b
c

Supplemental Figure 12. Fatty acid and glucose levels and the metabolism potentials in bone. (A and B) Blood and marrow glucose at specific time points in the glucocorticoid-induced bone loss model (A, n=5) or fracture healing model (B, n=6~7). (C and D) Blood and marrow fatty acids (FAs) at specific time points in the glucocorticoid-induced bone loss model (E, n=5~8) or fracture healing model (F, n=6~7). (E) Representative immunofluorescent staining of Glut1 and Cpt1a of metatarsals (left) and callus (right). Data are mean ±SD. *$P < 0.05$, **$P < 0.01$, ***$P < 0.001$ by two-way ANOVA (A, B and C) with Bonferroni *post hoc* test or two-tailed Student's *t*-test (B and D).

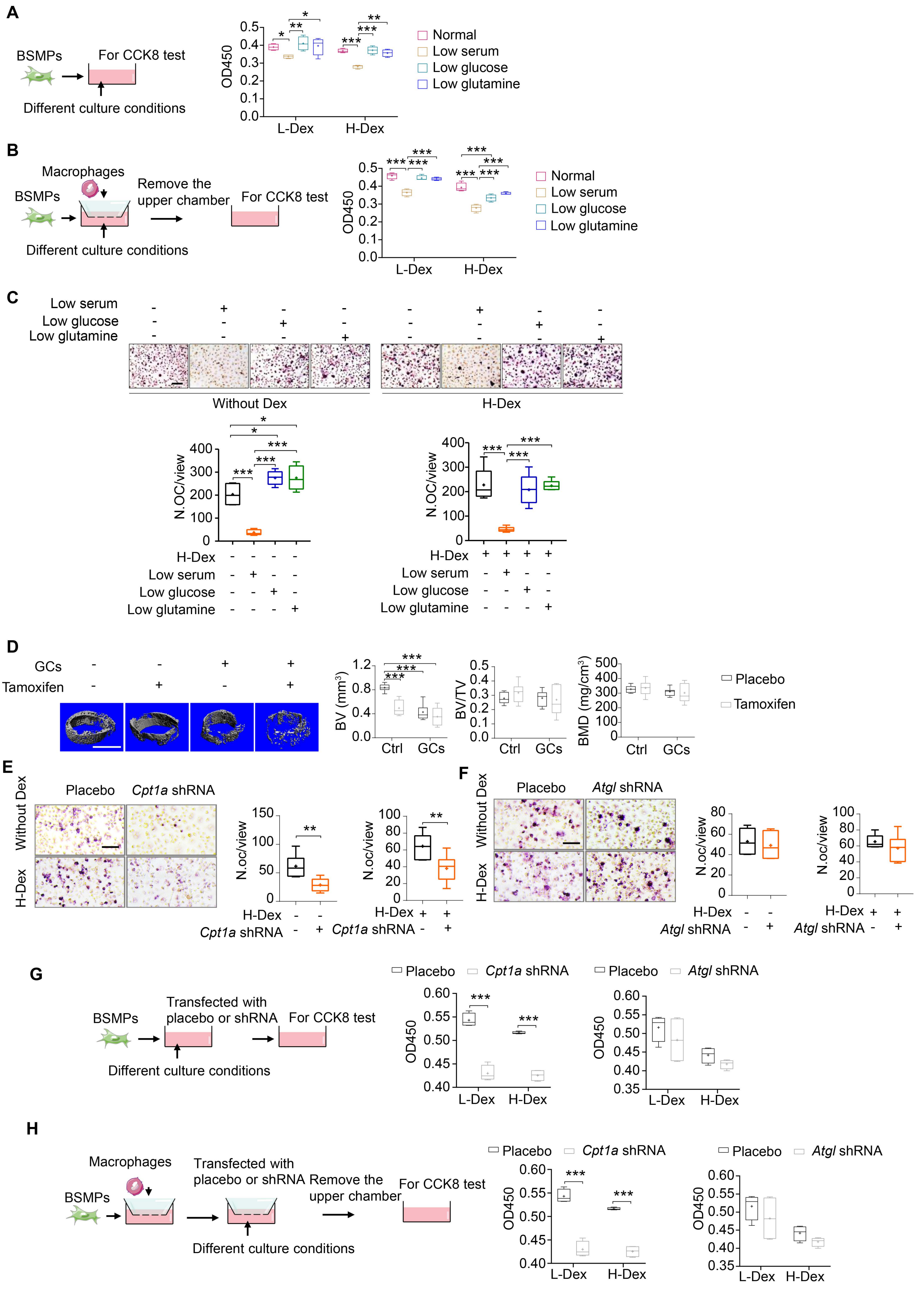

A
BSMPs
For CCK8 test
Different culture conditions
OD450
L-Dex
H-Dex
Normal
Low serum
Low glucose
Low glutamine
B
Macrophages
BSMPs
Remove the upper chamber
For CCK8 test
Different culture conditions
C
Low serum
Low glucose
Low glutamine
Without Dex
H-Dex
N.OC/view
D
GCs
Tamoxifen
BV (mm3)
BV/TV
BMD (mg/cm3)
Ctrl
GCs
Placebo
Tamoxifen
E
Without Dex
H-Dex
Placebo
Cpt1a shRNA
N.oc/view
F
Atgl shRNA
G
Transfected with placebo or shRNA
H

Supplemental Figure 13. Fatty acid oxidation regulates bone formation and resorption. (A) CCK-8 assay of BSMPs under various nutritional conditions (n=4). (B) CCK-8 assay of BSMPs with the co-culture of macrophages under various nutritional conditions (n=4). (C) Osteoclast differentiation of macrophages exposed to control or different nutritional stresses, as assessed by TRAP staining and the quantification of the number of osteoclasts per view (n=5; scale bar, 500 μm). (D) Representative micro-CT images and quantification (callus distal ends) of BMD, BV/TV and BV in the callus from vehicle control (ctrl) and 6.25 mg/kg/d of prednisolone (GCs)-treated mice with or without injection of tamoxifen (for global Cpt1a depletion) (n= 5~7/time point; scale bar, 500 μm). (E-F) Osteoclast differentiation of macrophages transfected with mock-vehicle, *Cpt1a* shRNA (E) or *Atgl* shRNA (F), as assessed by TRAP staining and the quantification of the number of osteoclasts per view (N.oc/view) (n=6~7; scale bar, 500 μm). (G) CCK-8 assay of BSMPs transfected with mock-vehicle, *Cpt1a* shRNA or *Atgl* shRNA (n=4). (H) CCK-8 assay of BSMPs with the co-culture of macrophages transfected with vehicle, *Cpt1a* shRNA or *Atgl* shRNA(n=4). L-Dex, 10^{-8} M Dex; H-Dex, 10^{-6} M Dex. Data are mean ± SD. **P* < 0.05, ***P* < 0.01, ****P* < 0.001 by two-way ANOVA (A, B, D, G and H), one-way ANOVA (C) with Bonferroni *post hoc* test or two-tailed Student's *t*-test (E and F).

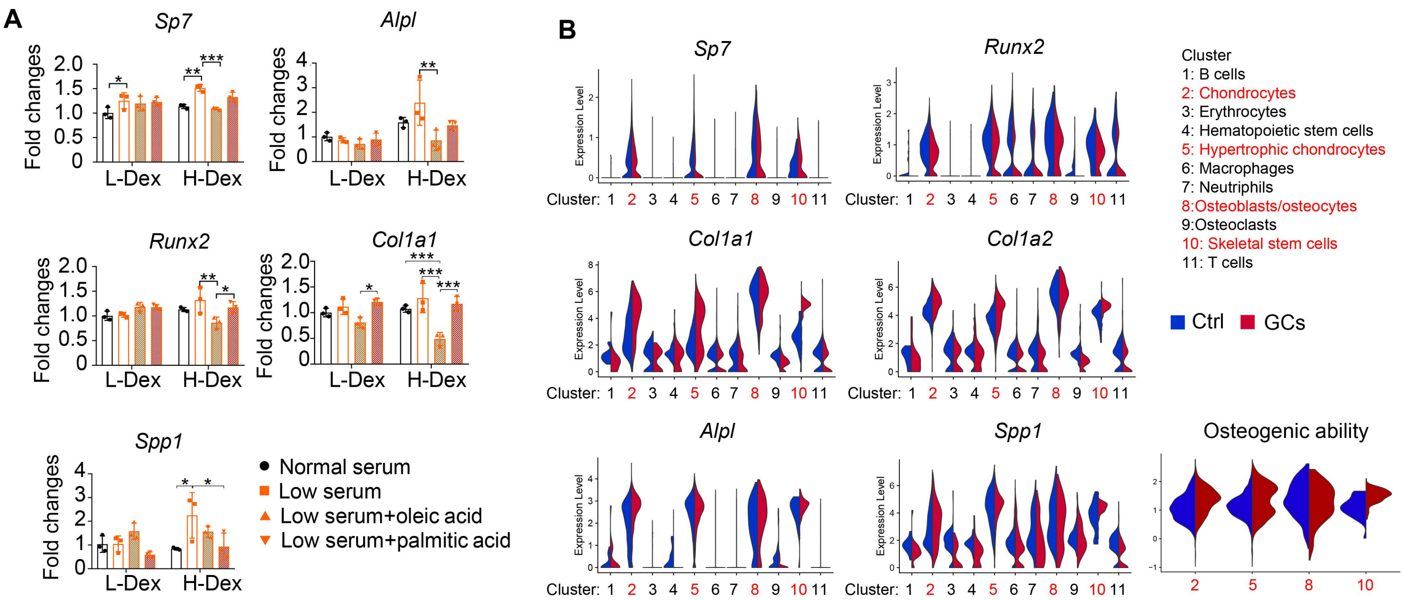
A
Sp7
Alpl
Runx2
Col1a1
Spp1
Fold changes
L-Dex
H-Dex
Normal serum
Low serum
Low serum+oleic acid
Low serum+palmitic acid
B
Sp7
Runx2
Col1a1
Col1a2
Alpl
Spp1
Expression Level
Cluster: 1 2 3 4 5 6 7 8 9 10 11
Cluster
1: B cells
2: Chondrocytes
3: Erythrocytes
4: Hematopoietic stem cells
5: Hypertrophic chondrocytes
6: Macrophages
7: Neutriphils
8:Osteoblasts/osteocytes
9:Osteoclasts
10: Skeletal stem cells
11: T cells
Ctrl
GCs
Osteogenic ability
2
5
8
10

Supplemental Figure 14. Glucocorticoid exposure and serum deprivation jointly determine the transcriptional levels of osteogenic genes. (A) Transcription levels of BSMPs exposed to normal or low serum conditions with or without the supplements of fatty acids (palmitic acid and oleic acid) after 3 days of osteogenic induction (n=3). (B) Normalized expression levels of osteogenic differentiation-associated genes in different clusters of the week 2 fracture calluses as shown by violin plots. L-Dex, 10^{-8} M Dex; H-Dex, 10^{-6} M Dex. Data are mean ±SD (A). *$P < 0.05$, **$P < 0.01$, ***$P < 0.001$ by two-way ANOVA with Bonferroni *post hoc* test.

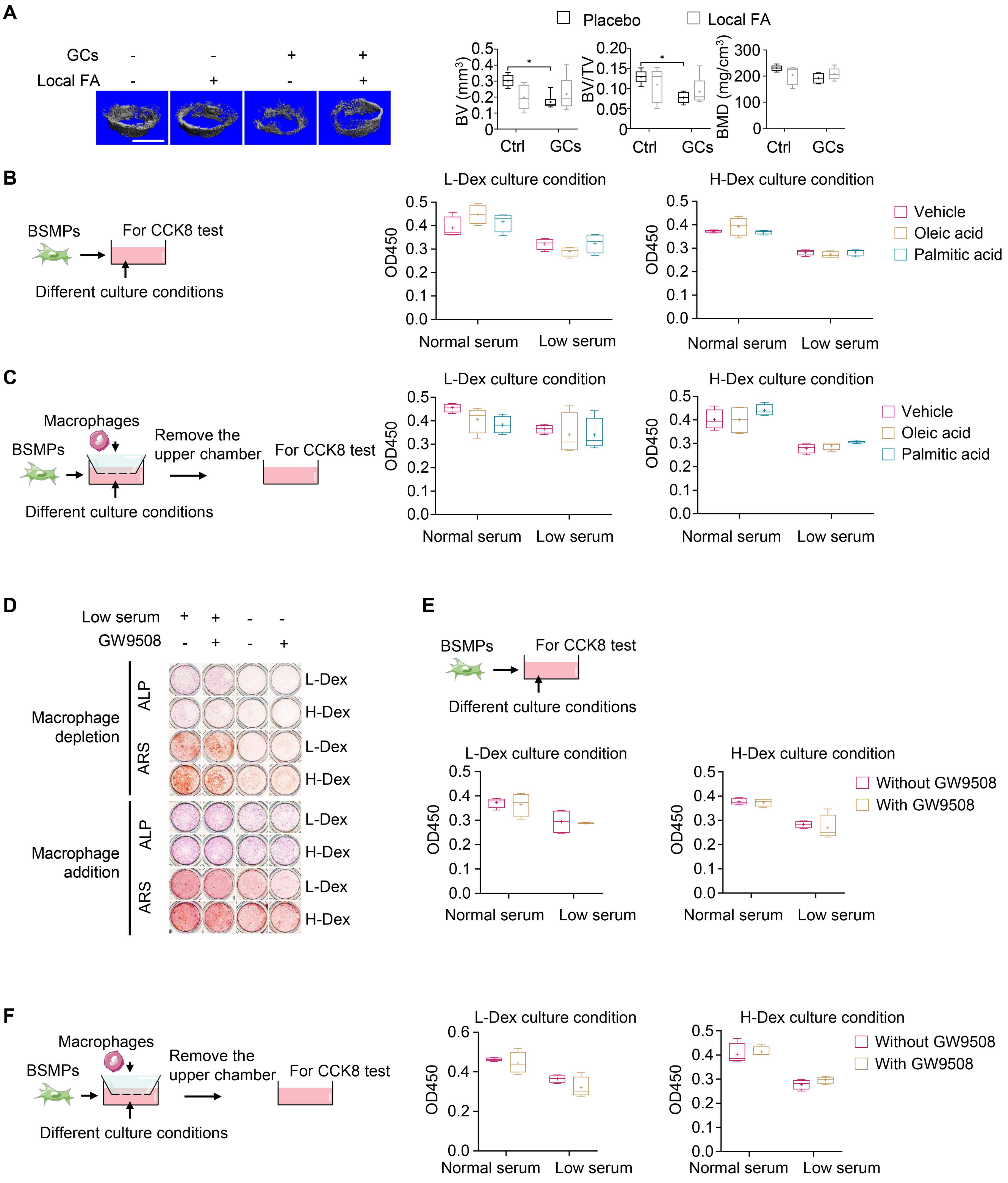

A
GCs
Local FA
Placebo
Local FA
BV (mm3)
BV/TV
BMD (mg/cm3)
Ctrl
GCs
B
BSMPs
For CCK8 test
Different culture conditions
L-Dex culture condition
H-Dex culture condition
OD450
Normal serum
Low serum
Vehicle
Oleic acid
Palmitic acid
C
Macrophages
BSMPs
Remove the upper chamber
For CCK8 test
Different culture conditions
D
Low serum
GW9508
Macrophage depletion
Macrophage addition
ALP
ARS
L-Dex
H-Dex
E
Without GW9508
With GW9508
F

Supplemental Figure 15. Delivery of fatty acids or activation of fatty acid signaling during osteogenesis. (A) Representative micro-CT images and quantification (callus distal ends) of BMD, BV/TV and BV in the callus from vehicle control (Ctrl) and 6.25 mg/kg/d of prednisolone (GCs)-treated mice with or without local injection of fatty acids (FA) (n= 5~6/time point; scale bar, 500 μm). (B) CCK-8 assay of BSMPs exposed to different serum conditions and fatty acids (n=4). (C) CCK-8 assay of BSMPs with the co-culture of macrophages exposed to different serum conditions and fatty acids (n=4). (D) Representative ALP or ARS staining of BSMPs (with or without addition of macrophages) exposed to normal serum or low serum condition, with or without the addition of GW9508 during osteogenic differentiation. (E) CCK-8 assay of BSMPs exposed to different serum conditions and GW9508 (n=4). (F) CCK-8 assay of BSMPs with the co-culture of macrophages exposed to different serum conditions and GW9508 (n=4). L-Dex, 10^{-8} M Dex; H-Dex, 10^{-6} M Dex. Data are mean ±SD. *$P < 0.05$ by two-way ANOVA (A, B, C, E and F) with Bonferroni *post hoc* test.

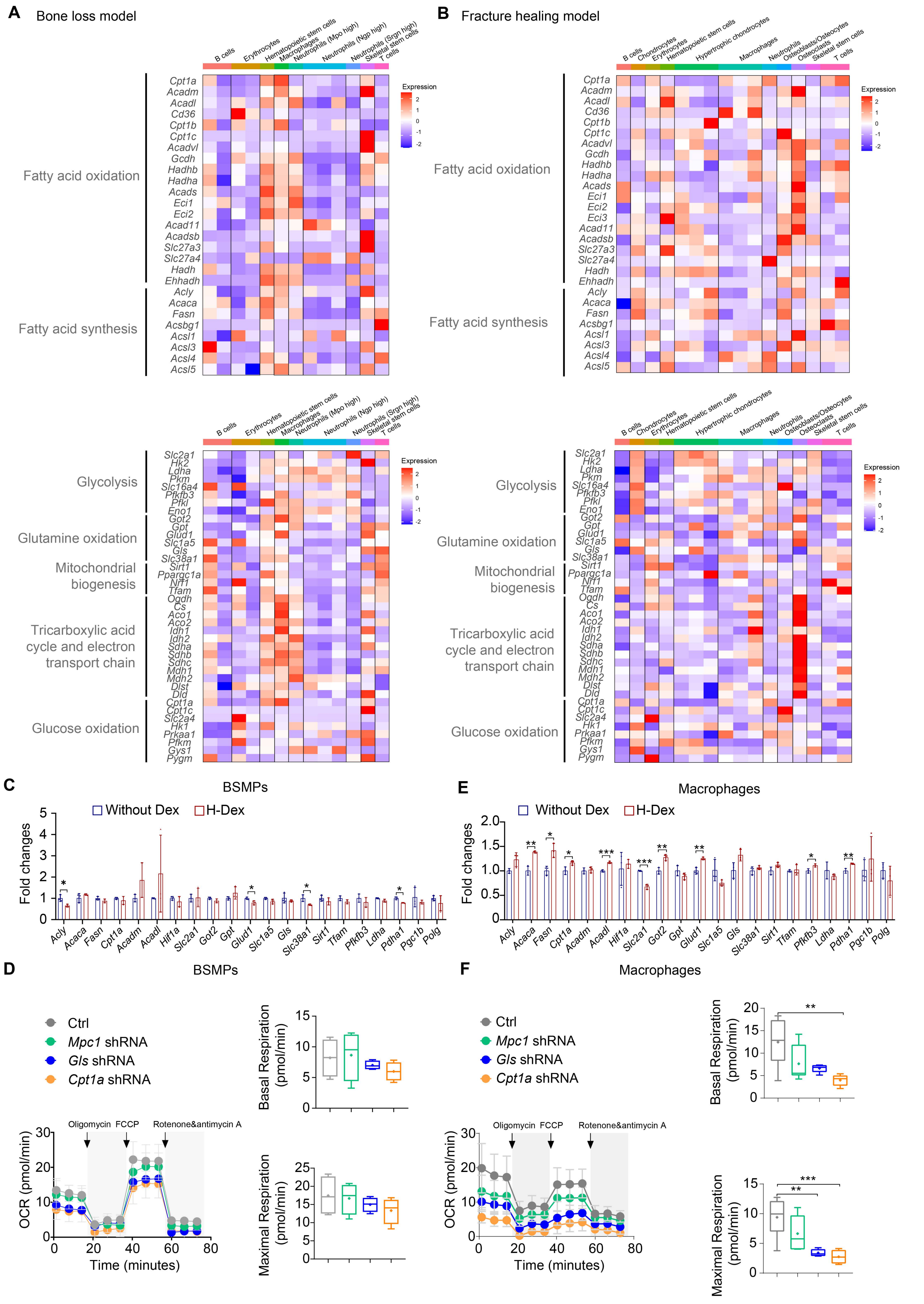

A Bone loss model
B Fracture healing model
Fatty acid oxidation
Fatty acid synthesis
Glycolysis
Glutamine oxidation
Mitochondrial biogenesis
Tricarboxylic acid cycle and electron transport chain
Glucose oxidation
Expression
C BSMPs
Without Dex
H-Dex
Fold changes
E Macrophages
D BSMPs
F Macrophages
Ctrl
Mpc1 shRNA
Gls shRNA
Cpt1a shRNA
Oligomycin FCCP Rotenone&antimycin A
OCR (pmol/min)
Time (minutes)
Basal Respiration (pmol/min)
Maximal Respiration (pmol/min)

Supplemental Figure 16. Energy metabolism patterns of bone forming cells and the associated macrophage pool. (A and B) Expression levels of energy metabolism-associated genes in different clusters of bone surface (A) and week 2 fracture callus (B). (C) Expression levels of energy metabolism-associated genes in BSMPs in culture upon exposure to H-Dex (n=3). (D) Cellular substrate oxidation measurement in BSMPs transfected with vehicle (Ctrl), *Mpc1* shRNA, *Gls* shRNA or *Cpt1a* shRNA (n=4) in culture upon exposure to H-Dex. (E) Expression levels of energy metabolism-associated genes in macrophages in culture upon exposure to H-Dex (n=3). (F) Cellular substrate oxidation measurement in macrophages transfected with vehicle (Ctrl), *Mpc1* shRNA, *Gls* shRNA or *Cpt1a* shRNA (n=5~6) after exposure to 10^{-6} M Dex for 1 day). H-Dex, 10^{-6} M Dex. Data are mean ± SD. *$P < 0.05$, **$P < 0.01$, ***$P < 0.001$ by one-way ANOVA (D and F) with Bonferroni *post hoc* test or two-tailed Student's *t*-test (C and E).

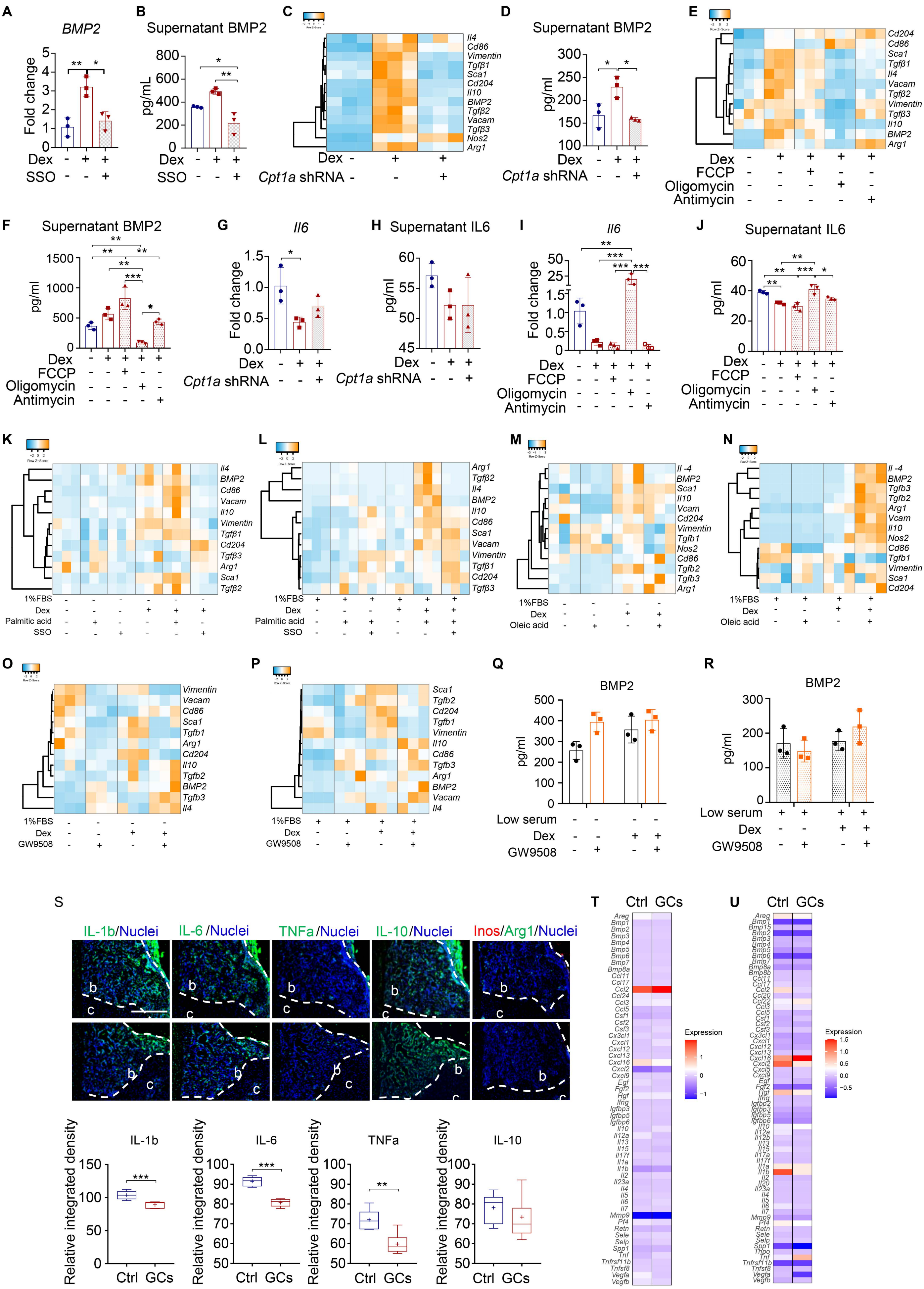

A
B
C
D
E
F
G
H
I
J
K
L
M
N
O
P
Q
R
S
T
U
BMP2
Supernatant BMP2
Supernatant IL6
Il6
Fold change
pg/mL
pg/ml
Dex
SSO
Cpt1a shRNA
FCCP
Oligomycin
Antimycin
1%FBS
Palmitic acid
Oleic acid
GW9508
Low serum
IL-1b/Nuclei
IL-6 /Nuclei
TNFa/Nuclei
IL-10/Nuclei
Inos/Arg1/Nuclei
Relative integrated density
IL-1b
IL-6
TNFa
IL-10
Ctrl
GCs
Expression

Supplemental Figure 17. Fatty acid metabolism regulates glucocorticoid-associated polarization of macrophages. (A-B) Transcription levels (A) and supernatant concentration (B) of BMP2 in macrophages exposed to sulfo-N-succinimidyl oleate (SSO) (n=3). (C) Transcription levels of M2 polarization-associated genes in macrophages under the exposure to Dex transfected with vehicle or *Cpt1a* shRNA. (D)Supernatant concentration of BMP2 in macrophages transfected with Cpt1a shRNA (n=3). (E) Transcription levels of M2 polarization-associated genes in macrophages under the exposure to FCCP, oligomycin and/or antimycin. (F) Supernatant concentration of BMP2 in macrophages exposed to FCCP, oligomycin and/or antimycin (n=3). (G-J) Transcription levels (G, I) and supernatant concentration (H, J) of IL-6 in macrophages exposed to different culture conditions. (G, H) transfected with *Cpt1a* shRNA (n=3). (I, J) with FCCP, oligomycin and/or antimycin (n=3). (K and L) Under normal serum (10% FBS) (K) or low serum condition (1% FBS) (L), transcription levels of M2 polarization-associated genes in macrophages exposed to palmitic acid and/or sulfo-N-succinimidyl oleate (SSO). (M and N) Under normal serum (10% FBS) (M) or low serum condition (1% FBS) (N), transcription levels of M2 polarization-associated genes in macrophages when exposed to oleic acid. (O and P) Under normal serum condition (10% FBS) (O) or low serum condition (1% FBS) (P), transcription levels of M2 polarization-associated genes in macrophages exposed to GW9508. (Q-R) Under normal serum (10% FBS, Q) or low serum condition (1% FBS, R), BMP2 production from macrophages exposed to GW9508. (S) Immunofluorescence analysis and quantification of IL-1b, IL-6, IL-10 and TNFa in the glucocorticoid-associated fracture healing model (Scale bar, 250 μm; n=5~7). (T and U) Expression levels of cytokines in macrophages in the glucocorticoid-induced bone loss (T) and associated fracture healing model (U). Data are mean ±SD. $^{*}P < 0.05$, $^{**}P < 0.01$, $^{***}P < 0.001$ by one-way ANOVA (A, B, D, F, G, H, I and J), two-way ANOVA (Q and R) with Bonferroni *post hoc* test or two-tailed Student's *t*-test (S).

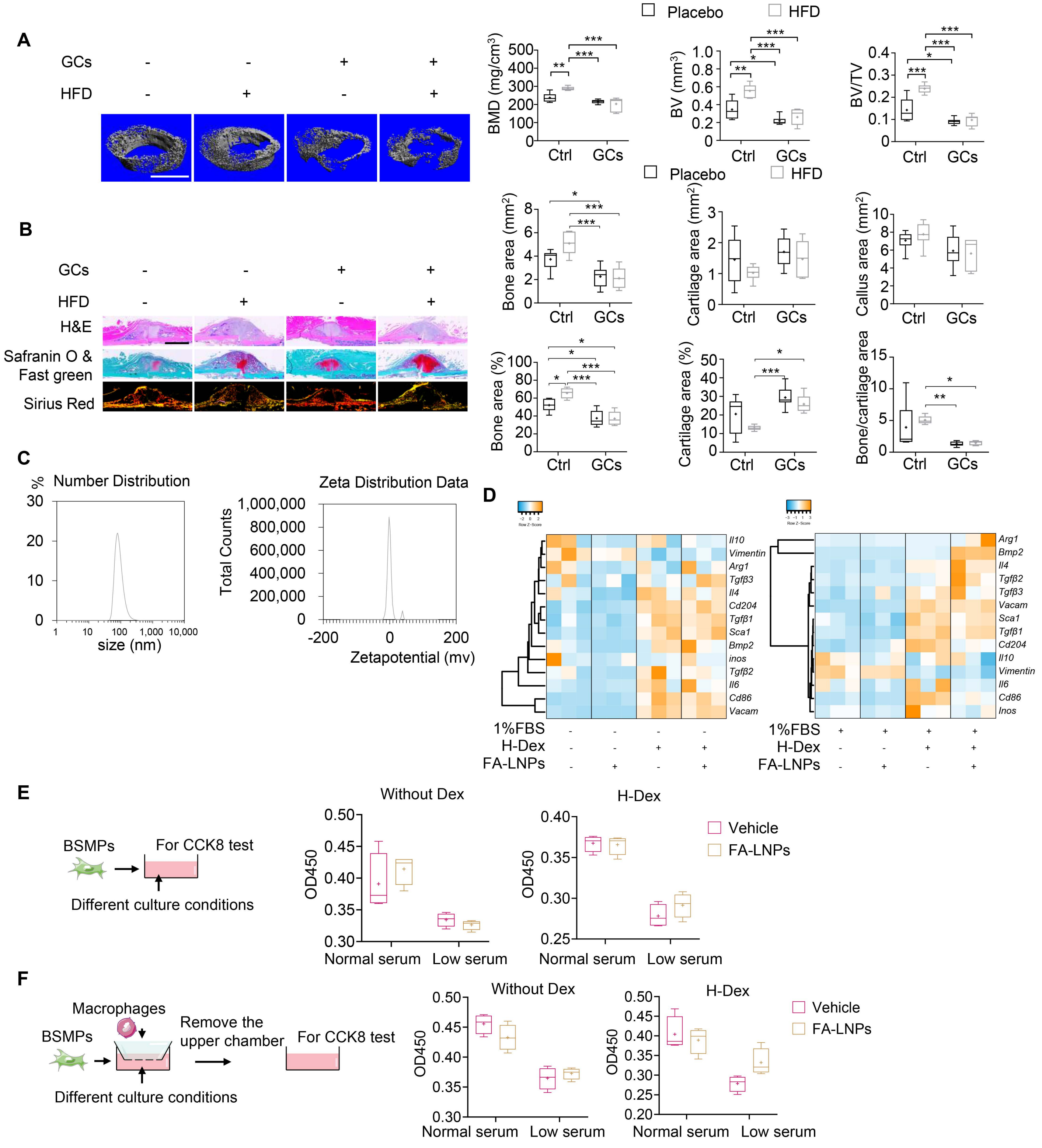

A
GCs
HFD
Placebo
HFD
BMD (mg/cm³)
BV (mm³)
BV/TV
Ctrl
GCs
B
H&E
Safranin O & Fast green
Sirius Red
Bone area (mm²)
Cartilage area (mm²)
Callus area (mm²)
Bone area (%)
Cartilage area (%)
Bone/cartilage area
C
Number Distribution
%
size (nm)
Zeta Distribution Data
Total Counts
Zetapotential (mv)
D
Il10
Vimentin
Arg1
Tgfβ3
Il4
Cd204
Tgfβ1
Sca1
Bmp2
inos
Tgfβ2
Il6
Cd86
Vacam
1%FBS
H-Dex
FA-LNPs
E
BSMPs
For CCK8 test
Different culture conditions
Without Dex
H-Dex
OD450
Normal serum
Low serum
Vehicle
FA-LNPs
F
Macrophages
BSMPs
Remove the upper chamber
For CCK8 test
Different culture conditions

Supplemental Figure 18. Systemic and local fatty acid delivery strategies to improve fracture healing. (A) Representative micro-CT images and quantification (callus distal ends) of BMD, BV/TV and BV in the callus from vehicle control (Ctrl) and 6.25 mg/kg/d of prednisolone (GCs)-treated mice with or without high-fat diet (HFD) feeding (n= 6~8/time point; scale bar, 500 μm). (B) Representative H&E, Safranin O & Fast green, and Sirius red staining and quantification of callus from vehicle control (ctrl) and 6.25 mg/kg/d of prednisolone (GCs)-treated mice with or without HFD feeding (n= 6~8/time point; scale bar, 1mm). (C) Size distribution and zeta potential of FA-LNPs (scale bar, 50 μm). (D) Under normal serum condition (10% FBS) or low serum condition (1% FBS), transcription levels of M2 polarization-associated genes in macrophages with the selective exposure of H-Dex and FA-LNPs. (E) CCK-8 assay of BSMPs exposed to different serum conditions and FA-LNPs (n=4). (F) CCK-8 assay of BSMPs with the co-culture of macrophages exposed to different serum conditions and FA-LNPs (n=4). Data are mean ± SD. *$P < 0.05$, **$P < 0.01$, ***$P < 0.001$ by two-way ANOVA (A, B, E and F) with Bonferroni *post hoc* test.

Table S1

Name	Concentration (µg/ml)
Caprylic acid	N.D
Decanoic acid	N.D
Undecanoic acid	N.D
Dodecanoic acid	N.D
Tridecanoic acid	N.D
Myristic acid	0.921395
Trans-9-myristic acid	N.D
Cis-9-myristic acid	N.D
Pentadecanoic acid	0.839133
Trans-10-pentadecanoic acid	0.25136
Cis-10-pentadecanoic acid	N.D
Palmitic acid	50.09251
Trans-9-palmitoleic acid	N.D
Cis-9-palmitoleic acid	2.108188
Heptadecanoic acid	2.054215
Trans-10-heptadecenoic acid	N.D
Cis-10-heptadecenoic acid	N.D
Stearic acid	46.49769
Trans-6-octadecenoic acid	N.D
Trans-9-octadecenoic acid	0.981813
Trans-11-octadecenoic acid	N.D
Cis-6-octadecenoic acid	N.D
Cis-9-octadecenoic acid	41.77136
Cis-11-octadecenoic acid	13.52534
Trans-9,12, -linoleic acid	N.D
Cis-9,12, -linoleic acid	5.319904
Gamma-linolenic acid	0.414202
Trans-10-decanoenoic acid	N.D
Alpha-linolenic acid	0.272611
Eicosanoic acid	0.859647
Trans-11-eicosenoic acid	N.D
Cis-11-eicosenoic acid	0.948128
Cis-11, 14-eicosadienoic acid	1.636571
Eicosanoic acid	N.D
Cis-8,11, 14-linolenic acid	6.731601
Arachidonic acid	25.83128
Cis-11,14, 17-eicosatrienoic acid	0.403834
Docosanic acid	0.313541
Cis-5,8,11,14, 17-eicosapentaenoic acid	7.494086
Trans-13-docoenoic acid	0.324733
Cis-13-erucic acid	0.445231
Cis-13, 16-docosadienoic acid	N.D
Tricosanoic acid	N.D
Cis-7,10,13, 16-docosatetraenoic acid	4.243116
Cis-4,7,10,13, 16-docosenapentaenoic acid	1.894671

Lignoceric acid	0.294571
Cis-7,10,13,16, 19-docosapentaenoic acid	12.45476
Cis-15-neuric acid	N.D
Cis-4,7,10,13,16, 19-docosahexaenoic acid	19.5975
Methyl caprylate	N.D
Methyl caprate	N.D
Methyl undecanoate	N.D
Methyl laurate	N.D
Methyl tridecanoate	N.D
Methyl myristate	3.82048
Trans-9-methyl myristate	N.D
Methyl cis-9-myristate	N.D
Methyl pentadecanoate	3.84944
Methyl trans-10-pentadecanoate	1.99512
Methyl cis-10-pentadecanoate	N.D
Methyl palmitate	5.82012
Methyl trans-9-palmitate	N.D
Methyl cis-9-palmitate	4.10156
Methyl heptadecanoate	5.833
Methyl trans-10-heptadecenoate	N.D
Methyl cis-10-heptadecenoate	N.D
Methyl stearate	3.95508
Methyl trans-6-octadecenoate	N.D
Methyl trans-9-octadecenoate	6.02308
Methyl trans-11-octadecenoate	N.D
Methyl cis-6-octadecenoate	N.D
Methyl cis-9-octadecenoate	4.13248
Methyl cis-11-octadecenoate	4.00588
Trans-9, 12-methyl linoleate	N.D
Methyl cis-9,12, -linoleate	6.03204
Gamma-linolenic acid methyl ester	6.00192
Methyl trans-10-decanoenoate	N.D
Methyl alpha-linolenic acid	5.98032
Methyl eicosanoate	4.07912
Trans-11-eicosanoate methyl ester	N.D
Methyl cis-11-eicosanoate	3.97112
Methyl cis-11, 14-eicosadienoate	6.0908
Methyl eicosanoate	N.D
Methyl cis-8,11, 14-linolenic acid	3.98604
Methyl arachidonic acid	5.98688
Methyl cis-11,14, 17-eicosatrienoate	4.04964
Methyl docosanate	2.0124
Methyl cis-5,8,11,14, 17-eicosapentaenoate	4.01208
Methyl trans-13-docoenoate	2.02564
Methyl cis-13-erucate	4.17604
Methyl cis-13, 16-docosadienoate	N.D
Methyl tricosanoate	N.D

Methyl cis-7,10,13, 16-docosatetraenoate	4.13824
Methyl cis-4,7,10,13, 16-docosapentaenoate	3.95592
Methyl lignocerate	2.05884
Methyl cis-7,10,13,16, 19-docosapentaenoate	4.04304
Methyl cis-15-neurate	N.D
Methyl cis-4,7,10,13,16, 19-docosahexaenoate	4.0668

N.D, not detectable.

Table S2. Primers.

Gene name	Oligonucleotide primers (5'->3')
Col1a1-F	GCTCCTCTTAGGGGCCACT
Col1a1-R	CCACGTCTCACCATTGGGG
Col1a2-F	AAGGGTGCTACTGGACTCCC
Col1a2-R	TTGTTACCGGATTCTCCTTTGG
Ocn-F	CTTGGCCCAGACCTAGCAG
Ocn-R	CTGGGCTTGGCATCTGTGA
BMP2-F	GGGACCCGCTGTCTTCTAGT
BMP2-R	TCAACTCAAATTCGCTGAGGAC
Csf1-F	GTGTCAGAACACTGTAGCCAC
Csf1-R	TCAAAGGCAATCTGGCATGAAG
Cd36-F	AGATGACGTGGCAAAGAACAG
Cd36-R	CCTTGGCTAGATAACGAACTCTG
Il6-F	TAGTCCTTCCTACCCCAATTTC
Il6-R	TTGGTCCTTAGCCACTCCTTC
Col2a1-F	CAGGATGCCCGAAAATTAGGG
Col2a1-R	ACCACGATCACCTCTGGGT
Caspase3-F	TGGTGATGAAGGGGTCATTTATG
Caspase3-R	TTCGGCTTTCCAGTCAGACTC
Caspase6-F	GGAAGTGTTCGATCCAGCCG
Caspase6-R	GGAGGGTCAGGTGCCAAAAG
Il17-F	TTTAACTCCCTTGGCGCAAAA
Il17-R	CTTTCCCTCCGCATTGACAC
Tgfb1-F	CTCCCGTGGCTTCTAGTGC
Tgfb1-R	GCCTTAGTTTGGACAGGATCTG
Pdgfb-F	TCCGGCTGCTGCAATAACC
Pdgfb-R	GGCTTCTTTCGCACAATCTCAAT
Pdgfa-F	TGGCTCGAAGTCAGATCCACA
Pdgfa-R	TTCTCGGGCACATGGTTAATG
Igf1-F	GTGGATGCTCTTCAGTTCGTGTG
Igf1-R	TCCAGTCTCCTCAGATCACAGC
Inos-F	GGAGTGACGGCAAACATGACT
Inos-R	TCGATGCACAACTGGGTGAAC
Vegfa-F	CTGCTGTAACGATGAAGCCCTG
VegfA-R	GCTGTAGGAAGCTCATCTCTCC
Tgfb3-F	GGACTTCGGCCACATCAAGAA
Tgfb3-R	TAGGGGACGTGGGTCATCAC
Tgfb2-F	CTTCGACGTGACAGACGCT
Tgfb2-R	TTCGCTTTTATTCGGGATGATGT
Endomucin-F	GCACACACCATGTCACTGCTTC
Endomucin-R	CCAGCGCGATAACCACAGGC
Cadherin5-F	CACTGCTTTGGGAGCCTTC

Cadherin5-R	GGGGCAGCGATTCATTTTTCT
Icam1-F	TGCCTCTGAAGCTCGGATATAC
Icam1-R	TCTGTCGAACTCCTCAGTCAC
Nos3-F	GGCTGGGTTTAGGGCTGTG
Nos3-R	CTGAGGGTGTCGTAGGTGATG
Eselectin-F	ATGAAGCCAGTGCATACTGTC
Eselectin-R	CGGTGAATGTTTCAGATTGGAGT
Occludin-F	TACTGGTCTCTACGTGGATCAAT
Occludin-R	TTCTTCGGGTTTTCACAGCAA
Caspase9-F	TCCTGGTACATCGAGACCTTG
Caspase9-R	AAGTCCCTTTCGCAGAAACAG
Ctnnb1-F	CCCAGTCCTTCACGCAAGAG
Ctnnb1-R	CATCTAGCGTCTCAGGGAACA
F11r-F	TCTCTTCACGTCTATGATCCTGG
F11r-R	TTTGATGGACTCGTTCTCGGG
Cd31-F	CTGCCAGTCCGAAAATGGAAC
Cd31-R	CTTCATCCACCGGGGCTATC
Tjp1-F	GCCGCTAAGAGCACAGCAA
Tjp1-R	TCCCCACTCTGAAAATGAGGA
Spp1-F	AGCAAGAAACTCTTCCAAGCAA
Spp1-R	GTGAGATTCGTCAGATTCATCCG
Acly-F	AATCCTGGCTAAAACCTCGCC
Acly-R	GCATAGATGCACACGTAGAACT
Acaca-F	ATGGGCGGAATGGTCTCTTTC
Acaca-R	TGGGGACCTTGTCTTCATCAT
Fasn-F	AGGTGGTGATAGCCGGTATGT
Fasn-R	TGGGTAATCCATAGAGCCCAG
Cpt1a-F	CTATGCGCTACTCGCTGAAGG
Cpt1a-R	GGCTTTCGACCCGAGAAGA
Acadm-F	AGGGTTTAGTTTTGAGTTGACGG
Acadm-R	CCCCGCTTTTGTCATATTCCG
Acadl-F	TCTTTTCCTCGGAGCATGACA
Acadl-R	GACCTCTCTACTCACTTCTCCAG
Hif1a-F	AGCTTCTGTTATGAGGCTCACC
Hif1a-R	TGACTTGATGTTCATCGTCCTC
Slc2a1-F	CAGTTCGGCTATAACACTGGTG
Slc2a1-R	GCCCCCGACAGAGAAGATG
Got2-F	TGGGCGAGAACAATGAAGTGT
Got2-R	CCCAGGATGGTTTGGGCAG
Gpt-F	AGCCTTTTACTGAGGTTATCCGT
Gpt-R	TCAGAAGATTGGGGTAGACACA
Glud1-F	CCCAACTTCTTCAAGATGGTGG
Glud1-R	AGAGGCTCAACACATGGTTGC

Slc1a5-F	GGTCTCCTGGATTATGTGGTACG
Slc1a5-R	AGCACAGAATGTATTTGCCGAG
Gls-F	GGGAATTCACTTTTGTCACGA
Gls-R	GACTTCACCCTTTGATCACC
Slc38a1-F	CCTTCACAAGTACCAGAGCAC
Slc38a1-R	GGCCAGCTCAAATAACGATGAT
Sirt1-F	GCTGACGACTTCGACGACG
Sirt1-R	TCGGTCAACAGGAGGTTGTCT
Tfam-F	ATTCCGAAGTGTTTTTCCAGCA
Tfam-R	TCTGAAAGTTTTGCATCTGGGT
Pfkfb3-F	CAACTCCCCAACCGTGATTGT
Pfkfb3-R	TGAGGTAGCGAGTCAGCTTCT
Ldha-F	TTCAGCGCGGTTCCGTTAC
Ldha-R	CCGGCAACATTCACACCAC
Pdha1-F	TGTGACCTTCATCGGCTAGAA
Pdha1-R	TGATCCGCCTTTAGCTCCATC
Pgc1b-F	TGACGTGGACGAGCTTTCAC
Pgc1b-R	GGGTCTTCTTATCCTGGGTGC
Polg-F	GCAGGAACAGTTAGTGGTGGG
Polg-R	GCTCATAGTATCGAGAAAACGCA
Cd86-F	TGTTTCCGTGGAGACGCAAG
Cd86-R	TTGAGCCTTTGTAAATGGGCA
Cd11b-F	CAGATCAACAATGTGACCGTATGGG
Cd11b-R	CATCATGTCCTTGTACTGCCGCTTG
Cd204-F	TGGAGGAGAGAATCGAAAGCA
Cd204-R	CTGGACTGACGAAATCAAGGAA
Arg1-F	CTCCAAGCCAAAGTCCTTAGAG
Arg1-R	AGGAGCTGTCATTAGGGACATC
Sca1-F	CTCCCAAGAAACGTGAGATCC
Sca1-R	CCATTCCTTGTAAACCATGCTCC
Vimentin-f	TCCACACGCACCTACAGTCT
Vimentin-R	CCGAGGACCGGGTCACATA
Vcam-1-f	TTCGGTTGTTCTGACGTGTG
Vcam-1-R	TACCACCCCATTGAGGGGAC
Ki67-F	ATCATTGACCGCTCCTTTAGGT
Ki67-R	GCTCGCCTTGATGGTTCCT
Gapdh-F	AACGACCCCTTCATTGAC
Gapdh-R	TCCACGACATACTCAGCAC
Pcna -F	TTTGAGGCACGCCTGATCC
Pcna -R	GGAGACGTGAGACGAGTCCAT
Il-6-F	TAGTCCTTCCTACCCCAATTTC
Il-6-R	TTGGTCCTTAGCCACTCCTTC
Atp6v0d2-F	AGCCAGCCTAACTCAGC

Atp6v0d2-R	GCTTCTTCCTCATCTCCGTGTC
Alpl-F	CCAACTCTTTTGTGCCAGAGA
Alpl-R	GGCTACATTGGTGTTGAGCTTTT
Sp7-F	ATGGCGTCCTCTCTGCTTG
Sp7-R	TGAAAGGTCAGCGTATGGCTT
Runx2-F	TTCAACGATCTGAGATTTGTGGG
Runx2-R	GGATGAGGAATGCGCCCTA
Nfatc1-F	GACCCGGAGTTCGACTTCG
Nfatc1-R	TGACACTAGGGGACACATAACTG
Ctsk-F	GAAGAAGACTCACCAGAAGCAG
Ctsk-R	TCCAGGTTATGGGCAGAGATT
Il-1b-F	TTCAGGCAGGCAGTATCACTC
Il-1b-R	GAAGGTCCACGGGAAAGACAC
Il-10-F	GCTCTTACTGACTGGCATGAG
Il-10-R	CGCAGCTCTAGGAGCATGTG
Tnfa-F	CCTGTAGCCCACGTCGTAG
Tnfa-R	GGGAGTAGACAAGGTACAACCC
Mmp9-F	CTGGACAGCCAGACACTAAAG
Mmp9-R	CTCGCGGCAAGTCTTCAGAG
Bcl2-F	ATGCCTTTGTGGAACTATATGGC
Bcl2-R	GGTATGCACCCAGAGTGATGC
Il1a-F	CGAAGACTACAGTTCTGCCATT
Il1a-R	GACGTTTCAGAGGTTCTCAGAG
Il-3-F	TTTCGGAGAGTAAACCTGTCCA
Il-3-R	AGGCAGGCAACAGTTAAGTTTC
Il-4-F	GGTCTCAACCCCCAGCTAGT
Il-4-R	GCCGATGATCTCTCTCAAGTGAT
Il-5-F	CTCTGTTGACAAGCAATGAGACG
Il-5-R	TCTTCAGTATGTCTAGCCCCTG
Il-12 (p40)-F	GGAAGCACGGCAGCAGAATAA
Il-12 (p40)-R	CTTGAGGGAGAAGTAGGAATG
Il13-F	CCTGGCTCTTGCTTGCCTT
Il13-R	GGTCTTGTGTGATGTTGCTCA
Ccl12-F	ATTTCCACACTTCTATGCCTCCT
Ccl12-R	ATCCAGTATGGTCCTGAAGATCA
Gcsf-F	ATGGCTCAACTTTCTGCCCAG
Gcsf-R	CTGACAGTGACCAGGGGAAC
Gmcsf-F	GGCCTTGGAAGCATGTAGAGG
Gmcsf-R	GGAGAACTCGTTAGAGACGACTT
Ifnγ-F	ACAGCAAGGCGAAAAAGGATG
Ifnγ-G	TGGTGGACCACTCGGATGA
Cxcl1-F	ACTGCACCCAAACCGAAGTC
Cxcl1-R	TGGGGACACCTTTTAGCATCTT

Ccl2-F	TTAAAAACCTGGATCGGAACCAA
Ccl2-R	GCATTAGCTTCAGATTTACGGGT
Ccl3-F	TTCTCTGTACCATGACACTCTGC
Ccl3-R	CGTGGAATCTTCCGGCTGTAG
Ccl4-F	TTCCTGCTGTTTCTCTTACACCT
Ccl4-R	CTGTCTGCCTCTTTTGGTCAG
Ccl5-F	GCTGCTTTGCCTACCTCTCC
Ccl5-R	TCGAGTGACAAACACGACTGC
