# Supplementary figures and images for: Excess glucocorticoids inhibit murine bone turnover via modulating the immunometabolism of the skeletal microenvironment
Source: J Clin Invest. 2024 Mar 21;134(10):e166795. doi: 10.1172/JCI166795 (PMC11093612; doi:10.1172/JCI166795)

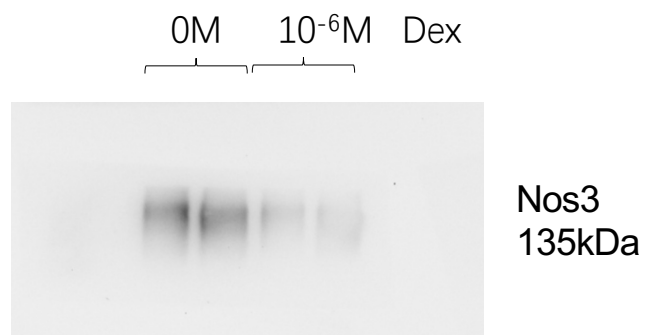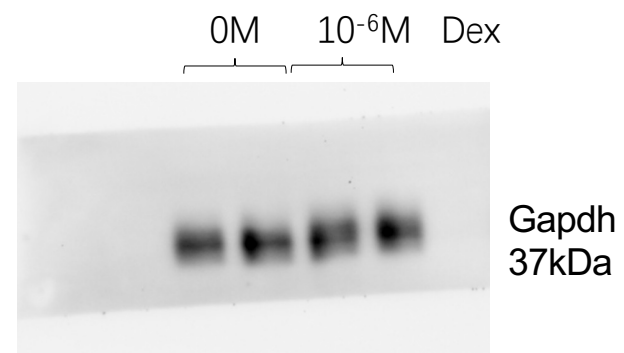

Supplement: Unedited blot and gel images [file jci-134-166795-s233.pdf]
